# iPLA_2_β: A novel store-operated calcium entry modulator contributing to muscle dysfunction during denervation

**DOI:** 10.1126/sciadv.aed1646

**Published:** 2026-07-17

**Authors:** Hongyang Xu, Shylesh Bhaskaran, Jacob Brown, Luis Gustavo Oliveira de Sousa, Constantin Georgescu, Elizabeth Duggan, Kara Kneuper, Ashley Bell, Jessica Thomason, Andy Gamez-Rico, Bo Hagy, Victoria Tyrrell, Valerie B. O’Donnell, Kenneth Humphries, Holly Van Remmen

**Affiliations:** ^1^ Aging and Metabolism Research Program, Oklahoma Medical Research Foundation, Oklahoma City, OK, USA.; ^2^ Oklahoma City VA Medical Center, Oklahoma City, OK, USA.; ^3^ Department of Health, Nutrition, and Food Sciences, Florida State University, Tallahassee, FL, USA.; ^4^ Systems Immunity Research Institute, School of Medicine, Cardiff University, Cardiff CF14 4XN, United Kingdom.

## Abstract

Sarcopenia, the age-related loss of skeletal muscle mass and strength, is a major cause of frailty and disability, with neuromuscular denervation as a key contributor. Bioactive lipid mediators, including lipid hydroperoxides and oxylipins, contribute to denervation-induced muscle atrophy and dysfunction. Here, we identify calcium-independent phospholipase A_2_β (iPLA_2_β) as a novel regulator of store-operated calcium ion (Ca^2+^) entry (SOCE), a critical process for maintaining Ca^2+^ homeostasis via stromal interaction molecule 1 (STIM1) and Orai1 coupling in skeletal muscle. Using muscle-specific iPLA_2_β knockout (miPLA_2_βKO) mice, we show that iPLA_2_β interacts with STIM1-Orai1 coupling to modulate SOCE. Denervation elevates iPLA_2_β, hyperactivating SOCE and causing Ca^2+^ overload through oxidative impairment of regulators such as SERCA. iPLA_2_β deletion normalizes SOCE, preserves Ca^2+^ homeostasis, and protects against denervation-induced muscle mass (5%) and strength loss (50%). These findings reveal that iPLA_2_β may be a critical link between oxidative stress and Ca^2+^ dysregulation and a promising target for mitigating muscle dysfunction during denervation.

## Teaser

Targeting iPLA_2_β enzyme restores muscle calcium homeostasis and helps preserve muscle function after loss of innervation.

## INTRODUCTION

Sarcopenia, the age-related loss of skeletal muscle mass and strength, is a major contributor to reduced mobility, frailty, and diminished quality of life in the elderly. This progressive condition substantially compromises both health span and life span, placing a growing burden on health care systems worldwide. One of the key drivers of sarcopenia is the gradual loss of neuromuscular innervation, which precipitates muscle atrophy and weakness through multiple molecular and cellular mechanisms, such as impaired mitochondrial function, elevated oxidative stress, and dysregulated Ca^2+^ homeostasis (*1*–*3*). Our recent studies have highlighted the role of bioactive oxidized lipid mediators (oxylipins), including lipid hydroperoxides (LOOHs), in mediating the deleterious effects during muscle denervation (*4*–*6*). Oxylipins are generated primarily through the action of phospholipase A_2_ (PLA_2_) enzymes, which catalyze the release of polyunsaturated fatty acids from membrane phospholipids, the precursor of a range of proinflammatory lipid species (*7*, *8*). Among PLA_2_ isoforms, calcium-independent PLA_2_β (iPLA_2_β), whose enzymatic activity is Ca^2+^ independent, has emerged as a key player not only in lipid metabolism but also in the regulation of intracellular calcium (Ca^2+^) signaling through modulating store-operated Ca^2+^ entry (SOCE) (*9*–*12*).

SOCE, the central focus of this study, is a critical mechanism for dynamic maintenance of cellular Ca^2+^ homeostasis through replenishing intracellular Ca^2+^ stores and sustaining Ca^2+^-dependent functions. SOCE is known to be mediated by the interaction between stromal interaction molecule 1 (STIM1), a sarcoplasmic reticulum (SR) Ca^2+^ sensor, and Orai1, a plasma membrane Ca^2+^ channel, when the SR is depleted of Ca^2+^ (*13*–*15*). In the past two decades, iPLA_2_β and its downstream lysophospholipids (LPLs) has been proposed to interfere with the STIM1-Orai1 coupling through direct activation of Orai1, thereby allowing rapid Ca^2+^ entry independent of STIM1 (*11*, *12*). The study by Várnai *et al.* (*16*) further elucidated that the formation of a direct interaction between STIM1 and Orai1 typically necessitates a large junctional space (12 to 14 nm). Their findings suggest that the narrow gap (4 to 6 nm) that normally exists between the endoplasmic reticulum (ER) and the plasma membrane is generally insufficient for STIM1-Orai1 complex formation. Consequently, they proposed that iPLA_2_β-mediated Orai1 activation might provide the primary pathway for SOCE in the majority of cell types (*16*). Studies examining the triad junction in skeletal muscle have revealed a consistent space of ∼12 to 14 nm between the SR terminal cisternae and the transverse tubular (t-tubular) membrane (*17*, *18*). This dimension is theoretically sufficient for the direct interaction and complex formation of STIM1 and Orai1, at least at the triad junction. However, the specific pathways governing SOCE activation in skeletal muscle remain elusive. Furthermore, the precise role of iPLA_2_β in mediating SOCE activity within skeletal muscle is currently unknown and will be investigated within the scope of this study.

Denervation is an important mediator of age-related muscle atrophy and weakness, and our group has previously shown that the denervation model approximates many of the phenotypes present in aging skeletal muscle with respect to loss of mass and strength, particularly the induction of PLA_2_ and elevated generation of LOOH (*4*). We hypothesize that denervation increases both the expression and activity of iPLA_2_β, which in turn drives aberrant SOCE through dysregulated Orai1 activation, thereby exacerbating mitochondrial dysfunction, oxidative stress, and Ca^2+^ imbalance in skeletal muscle. Moreover, we predict that genetic deletion of iPLA_2_β will mitigate these deleterious effects of denervation. In this study, we used a denervation mouse model to investigate the impact of altered iPLA_2_β levels on SOCE activity and overall Ca^2+^ homeostasis. Furthermore, we used a mouse model with muscle-specific deletion of iPLA_2_β (miPLA_2_βKO) to evaluate the role of iPLA_2_β in the formation of STIM1-Orai1 complex and the consequences of iPLA_2_β ablation on SOCE activity, Ca^2+^ regulation, and, ultimately, the muscle force production.

Our findings demonstrate that SOCE activity in skeletal muscle is mediated by STIM1 and suggest that iPLA_2_β is acting as a regulator that modulates Orai1-dependent Ca^2+^ entry. Notably, our results clearly demonstrate that the presence of iPLA_2_β impedes the formation of the STIM1-Orai1 complex. We further observed that increased iPLA_2_β levels following denervation hyperactivate SOCE, subsequently leading to cytosolic Ca^2+^ accumulation due to denervation-induced oxidative damages of intracellular Ca^2+^ regulators, such as SERCA. Consequently, the deletion of iPLA_2_β, which normalized SOCE in denervated muscles, preserved muscle mass and strength. Collectively, these findings identify a previously unrecognized role for lipid mediators in regulating Ca^2+^ homeostasis, muscle mass, and contractile function in skeletal muscle.

## RESULTS

### Validation of muscle-specific iPLA_2_β deletion and its effect on muscle mass and strength during denervation

**Fig. 1.**
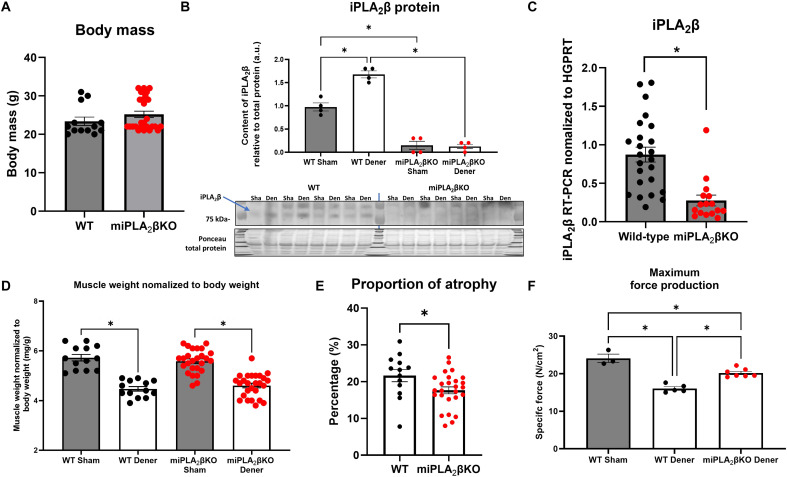
miPLA_2_βKO mouse model validation and muscle parameters. Validation of iPLA_2_β knockout in GTN muscle and assessment of the effects of denervation on iPLA_2_β expression, body and muscle mass, and muscle strength. (**A**) Body mass from WT and miPLA_2_βKO mice. (**B**) Representative Western blot images with pooled data for the relative amount of iPLA_2_β protein in sham (Sham, Sha) and denervated (Dener, Den) muscles from WT and miPLA_2_βKO mice. (**C**) RT-PCR analysis of iPLA_2_β expression levels in GTN muscles from WT and miPLA_2_βKO mice. (**D**) Muscle weight in sham and denervated muscles from WT and miPLA_2_βKO mice normalized to body weight. (**E**) Percentage of muscle loss in denervated GTN muscles relative to sham muscle in WT and miPLA_2_βKO mice. (**F**) Specific maximum force production in sham muscles from WT mice, and denervated muscles from WT and miPLA_2_βKO mice. * denotes significant difference between labeled groups [*P* < 0.05, Kruskal-Wallis tests, two-way analysis of variance (ANOVA) with Tukey’s post hoc correction]. *n* = 3 to 26 mice per group. Data are presented as means ± SEM. a.u., arbitrary unit.

The deletion of iPLA_2_β specifically in skeletal muscle was confirmed at both the mRNA and protein level using reverse transcription polymerase chain reaction (RT-PCR) and Western blot analysis, respectively, in whole gastrocnemius (GTN) muscle homogenates across all experimental groups. Deletion of iPLA_2_β did not change the body mass (Fig. 1A). As expected, iPLA_2_β expression was very low in muscle tissue in miPLA_2_β knockout (miPLA_2_βKO) mice, irrespective of denervation status (Fig. 1, B and C). Moreover, iPLA_2_β levels in other organs remained unaffected as confirmed by the tissue panel analysis by Western blot (fig. S1). Notably, iPLA_2_β protein levels were significantly elevated in denervated muscles compared to sham muscles in wild-type (WT) mice (Fig. 1B). Consistent with our previous findings, denervation in WT mice resulted in a significant decline in absolute GTN muscle mass (∼22%; Fig. 1, D and E) as compared to sham controls. A similar reduction in muscle mass was also observed in miPLA_2_βKO mice following denervation; however, the degree of atrophy (∼17%; Fig. 1, D and E) was significantly attenuated compared to WT counterparts. To assess muscle function, we measured the maximum specific force using in vitro electrical stimulation with extensor digitorum longus (EDL) muscle. In WT mice, denervation led to a substantial decline in muscle force generation (16 N/cm^2^) relative to sham muscles (24 N/cm^2^), aligning with our previous findings (*2*, *4*, *19*). Notably, deletion of iPLA_2_β significantly preserved muscle force in denervated muscles of miPLA_2_βKO mice (20 N/cm^2^), representing a ∼50% increase in force generation in miPLA_2_βKO compared to WT denervated muscles (Fig. 1F).

### Denervation leads to reduced cross-sectional area mainly in fast-twitch glycolytic fibers regardless of genotype

**Fig. 2.**
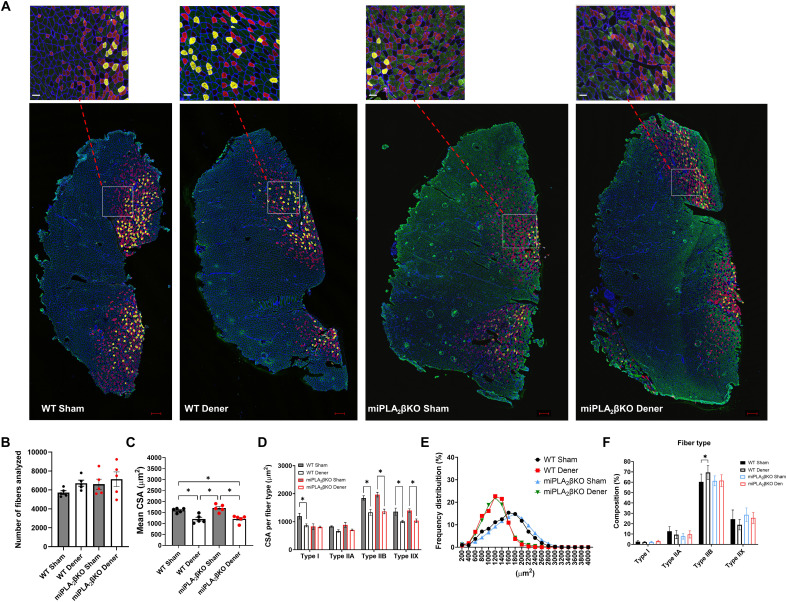
Denervation-induced changes in mean fiber type and CSA in WT and miPLA_2_βKO mice. Immunofluorescence staining was performed on whole cross-sections of GTN muscle to identify all four muscle fiber types: type I (yellow), type IIA (red), type IIB (green), type IIX (unstained/black), and laminin (blue) outlining sarcolemma. (**A**) Representative immunofluorescence images of muscle sections from WT and miPLA_2_βKO mice under sham and denervated conditions. (**B**) Total number of muscle fibers across different groups. (**C**) Average CSA of all fibers in each group. (**D**) CSA of individual fiber types across groups. (**E**) Fiber size distribution expressed as a percentage of total fibers in each group. (**F**) Fiber type composition shown as percentage of total fibers for each of the four fiber types in each group. Data are analyzed based on the whole muscle section. * denotes significant difference between labeled groups (*P* < 0.05, two-way ANOVA with Tukey’s post hoc correction). *n* = 5. Data are presented as means ± SEM. Red scale bar (main panels), 200 μm; white scale bar (zoomed-in insets), 50 μm.

To investigate the impact of denervation and the role of miPLA_2_β on muscle fiber composition, we performed whole-muscle immunofluorescence staining on GTN muscle sections from WT and miPLA_2_βKO mice under sham and denervated conditions (Fig. 2A). The four major muscle fiber types—type I, IIA, IIB, and IIX—were identified by specific labeling of distinct myosin heavy chain (MHC) isoforms. Quantification at the whole muscle cross-section level revealed no significant differences in total fiber number across groups (Fig. 2B), suggesting that denervation did not cause gross fiber loss within the time frame examined. However, denervation significantly reduced the average cross-sectional area (CSA) in both WT and miPLA_2_βKO mice (Fig. 2C), primarily due to pronounced atrophy of type IIB and IIX fibers (Fig. 2D). Knockout of iPLA_2_βKO did not exhibit a protective effect against this fiber-type specific atrophy. A significant reduction in type I fiber size was also observed in WT mice, which was not evident in the miPLA_2_βKO group (Fig. 2D). In both genotypes, denervation induced a visible shift in fiber size distribution toward smaller fibers (Fig. 2E), reflecting the generalized atrophic response. The distribution of muscle fiber types did not show major changes across groups. However, type IIB fibers represent a higher percentage of the total in WT muscles following denervation, whereas the other fiber types remained largely unchanged (Fig. 2F). Notably, this increase was not observed in iPLA_2_βKO muscles (Fig. 2F).

### Deletion of iPLA_2_β attenuates the hyperactivated SOCE following denervation and enhances STIM1-Orai1 interaction in GTN muscles

**Fig. 3.**
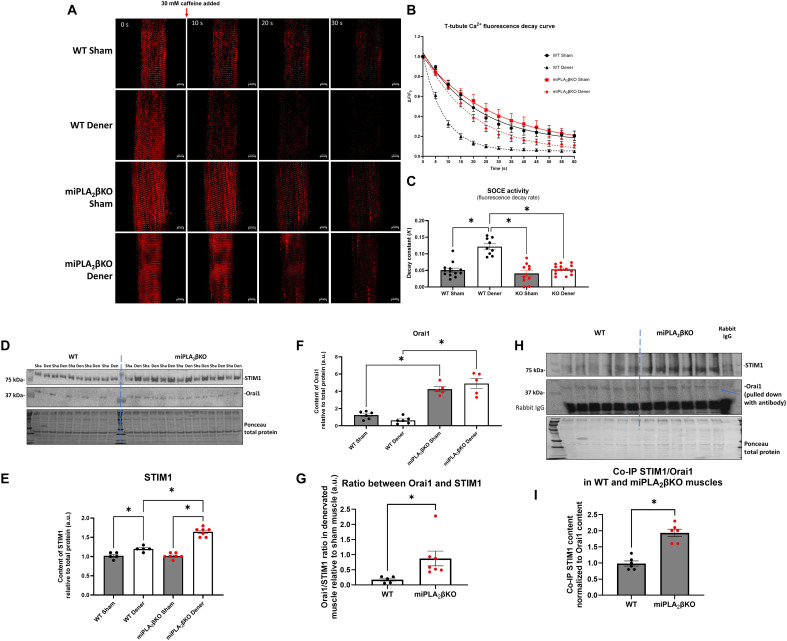
Measurement of SOCE activity in sham and denervated muscles in WT and miPLA_2_βKO mice and evaluation of the role of iPLA_2_β in modulating the interaction between STIM1 and Orai1. (**A**) SOCE activity is measured using confocal microscopy in skinned single fibers from sham and denervated muscles of WT and miPLA_2_βKO mice. Representative images captured at four time points (0, 10, 20, and 30 s) following the addition of 30 mM caffeine, illustrating SOCE-induced Ca^2+^ influx from the t-tubules into the cytosol over time. (**B**) Pooled data show the decay curve of the stained Ca^2+^ in t-tubules, indicating the influx of Ca^2+^ induced by SOCE, a faster fluorescence decay indicating a higher SOCE activity in the respective fiber. (**C**) SOCE activity in individual fibers was assessed using the decay constant (*K*) derived from the best-fit decay curve for each group. (**D**) Representative Western blot images show STIM1 and Orai1 protein levels in sham and denervated muscles from WT and miPLA_2_βKO mice. (**E** to **G**) Quantified data show the content of STIM1 and Orai1 relative to total protein, and the ratio of Orai1/STIM1 in denervated muscles relative to sham muscles from WT and miPLA_2_βKO mice. (**H** and **I**) Representative Western blot image and quantified data show immunoprecipitation of Orai1 using a specific antibody, followed by probing for STIM1 to assess the STIM1-Orai1 complex levels in GTN muscles from WT and miPLA_2_βKO mice. * denotes significant difference between labeled groups (*P* < 0.05, two-way ANOVA with Tukey’s post hoc correction). For SOCE measurements, data represent *n* = 9 to 14 fibers analyzed and *N* = 4 for the number of animals from which the fibers were obtained. For the Western blots, data represent results from *n* = 5 to 7 mice. Data are presented as means ± SEM. Co-IP, coimmunoprecipitation.

SOCE was assessed in single fast-twitch muscle fibers isolated from EDL muscles using confocal microscopy. T-tubules were loaded with a membrane-impermeable Ca^2+^ dye Rhod-5N, which was subsequently retained within the t-tubular system following fiber skinning. SOCE activity was activated by applying 30 mM caffeine to deplete SR Ca^2+^ storage. Upon SOCE activation, Ca^2+^ influx from t-tubules into the cytosol leads to a reduction in dye fluorescence within the t-tubules. The rate of fluorescence decline directly correlates with SOCE activity, i.e., a faster rate of t-tubule fading correlates with higher SOCE activity within that muscle fiber (Fig. 3A). To show the decay curve, we normalized Δ*F* (change in fluorescence) to *F*_0_ (initial fluorescence intensity before caffeine addition) at each time point (Fig. 3B), and the decay constant (*K*) derived from the best-fit decay curve for each group was used to indicate the rate of SOCE. In WT mice, we observed a significant increase in SOCE activity in denervated muscles compared to sham controls (∼2.4-fold faster), indicating a hyperactivation of Ca^2+^ influx following denervation (Fig. 3, A to C). An elevation in SOCE activity did not occur in miPLA_2_βKO mice, as denervated muscles have a level of SOCE activity comparable to that measured in WT sham muscles (Fig. 3, A to C). Consistently, pharmacological inhibition of iPLA_2_β with bromoenol lactone (BEL) attenuated the denervation-evoked SOCE hyperactivation (fig. S4), further supporting a critical role of iPLA_2_β in mediating this response. Western blot analysis revealed a significant increase in STIM1 protein levels in both WT and miPLA_2_βKO muscles following denervation (Fig. 3, D and E). Notably, Orai1 expression was markedly elevated in miPLA_2_βKO muscles compared to WT, under both sham and denervated conditions (Fig. 3F). As a result, the Orai1/STIM1 ratio was significantly increased in miPLA_2_βKO muscles, suggesting an improved availability of STIM1-Orai1 interactions during SOCE activity with the difference being most pronounced in denervated muscles (Fig. 3G). To further assess the functional interaction between STIM1 and Orai1, we performed coimmunoprecipitation assays using GTN muscle homogenates. Orai1 was immunoprecipitated followed by probing for STIM1 (Fig. 3H). The result demonstrated a significantly enhanced association between STIM1 and Orai1 (approximately twofold higher) in iPLA_2_βKO muscles compared to WT (Fig. 3E).

### SERCA activity remains impaired, but the overall Ca^2+^ homeostasis is improved in muscles from miPLA_2_βKO mice

**Fig. 4.**
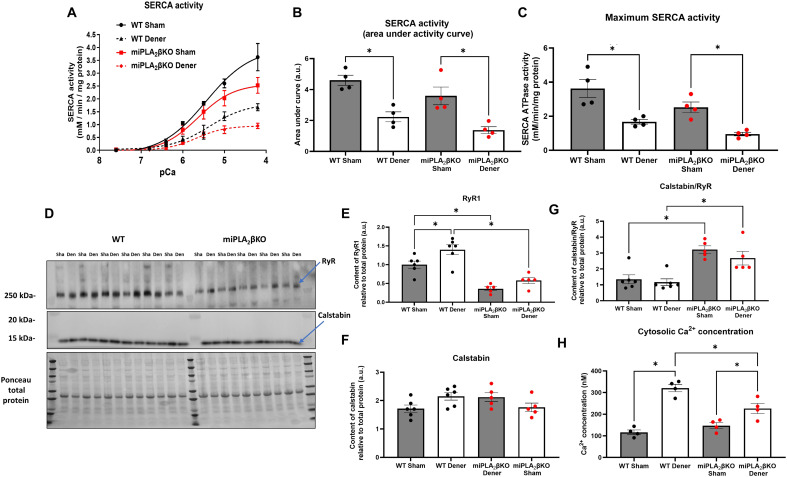
Activity and expression levels of key proteins involved in intracellular Ca^2+^ homeostasis, and detection of cytosolic Ca^2+^ accumulation. (**A**) SERCA activity assay measured at different Ca^2+^ concentrations (pCa) and (**B**) pooled data of the AUC and (**C**) the maximum SERCA activity. (**D** to **F**) Representative Western blot images with pooled data for the relative amount of RyR1 and its stabilizing protein calstabin in sham and denervated muscles from WT and miPLA_2_βKO mice. (**G**) Quantified relative amount of calstabin to the content of RyR across different groups. (**H**) Detection of cytosolic Ca^2+^ concentration in muscle fiber bundles in sham and denervated muscles from WT and miPLA_2_βKO mice. * denotes significant difference between labeled groups (*P* < 0.05, two-way ANOVA with Tukey’s post hoc correction). Data represent results from *n* = 4 to 6 mice. Data are presented as means ± SEM.

The SERCA pump in skeletal muscle is responsible for returning cytosolic Ca^2+^ back into the SR following muscle contraction, thereby maintaining low resting cytosolic Ca^2+^ levels. We measured SERCA activity in GTN muscle in a calcium concentration–dependent manner by generating a pCa-SERCA activity curve to assess activity across a range of Ca^2+^ levels (Fig. 4A). To quantify overall SERCA function, we calculated the area under the curve (AUC) (Fig. 4B) and additionally determined the maximum SERCA activity in each group using the activity value at the highest Ca^2+^ concentration (pCa 4.2) (Fig. 4C). In WT mice, SERCA activity was significantly reduced (∼50%) in denervated GTN muscles compared to sham controls, indicating compromised Ca^2+^ reuptake capacity. Notably, deletion of iPLA_2_β did not rescue this impairment, as SERCA activity remained similarly reduced in miPLA_2_βKO denervated muscles (Fig. 4, A to C). Western blot analysis confirmed that SERCA protein levels were unchanged across all groups (fig. S3A). We also measured total calsequestrin (CSQ) content and found that basal CSQ levels were elevated in iPLA_2_βKO muscles (fig. S3B). Moreover, with denervation, CSQ abundance was significantly higher in iPLA_2_βKO muscles compared with WT muscles, suggesting an enhanced SR Ca^2+^ storage capacity in iPLA_2_βKO muscles (fig. S3B). To gain further insight into cytosolic Ca^2+^ regulation, we also evaluated the amount of ryanodine receptor (RyR), the principal Ca^2+^ release channel on the SR membrane, and its stabilizing protein cofactor calstabin by Western blot (Fig 4D). RyR protein levels were markedly reduced 64% in sham muscles and 59% in denervated muscles in miPLA_2_βKO mice compared to WT mice (Fig. 4E). In contrast, calstabin content remained unchanged across all groups (Fig. 4F). As a result, the calstabin-to-RyR ratio was significantly increased in miPLA_2_βKO muscles (a 2.4-fold increase in sham and 2.3-fold increase in denervated muscles), suggesting enhanced RyR stabilization and a potentially reduced likelihood of SR Ca^2+^ leak (Fig. 4G). To directly assess cytosolic Ca^2+^ levels, we also measured Ca^2+^ concentrations in GTN muscle bundles using Fura-2 dye with a spectrofluorometer. Denervation induced a significant increase in cytosolic Ca^2+^ in both WT and miPLA_2_βKO muscles relative to their sham controls (Fig. 4H). However, cytosolic Ca^2+^ levels in denervated miPLA_2_βKO muscles were markedly lower than those in WT denervated muscles (Fig. 4H), suggesting a reduced Ca^2+^ accumulation. This improvement is likely attributable to normalized SOCE activity observed in miPLA_2_βKO mice.

### Membrane excitability is severely impaired in fast-twitch muscle fibers following denervation in WT mice but is partially preserved by iPLA_2_β deletion

**Fig. 5.**
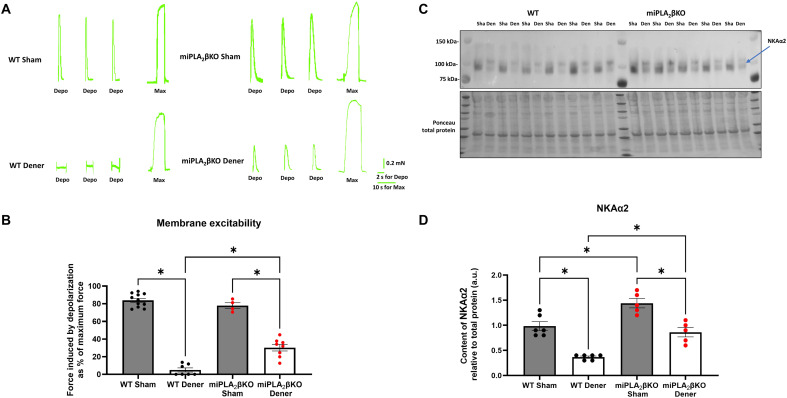
Membrane excitability in fast-twitch fibers from different groups and the amount of NKAα2. Membrane excitability in a skinned fiber was measured as the force response to depolarization induced by ionic substitution (referred to as “Depo” in the figure), expressed relative to the maximum Ca^2+^-activated force (referred to as “Max” in the figure) in that fiber. (**A**) Representative force trace of depolarization-induced force responses in single skinned fibers (upon exchange of K^+^-HDTA with Na^+^-HDTA solution), along with the maximum force production in that given fiber from sham and denervated muscles in WT and miPLA_2_βKO mice. (**B**) Pooled data of the depolarization-induced force responses relative to the maximum force production as a proportion. (**C** and **D**) Representative Western blot images with pooled data for the relative amount of NKAα2 in different groups. * denotes significant difference between labeled groups (*P* < 0.05, two-way ANOVA with Tukey’s post hoc correction). For membrane excitability measurements, data represent *n* = 4 to 11 fibers analyzed and *N* = 3 for the number of animals from which the fibers were obtained. For the Western blots, *n* = 5 to 6 animals. Data are presented as means ± SEM.

Membrane excitability was evaluated in individual skinned fast-twitch muscle fibers by measuring the force response elicited by transverse (t-system) depolarization through ionic substitution. Specifically, potassium-hexa-methylene-diamine-tetraacetate (K^+^-HDTA) was replaced with sodium-HDTA (Na^+^-HDTA), and the resulting depolarization-induced force response was expressed as a percentage of the maximum Ca^2+^-activated force produced by the same fiber. Under physiological conditions, t-system depolarization in fast-twitch fibers typically elicits a force response approximating 80% of the fiber’s maximum Ca^2+^-activated force. This level of excitability was observed in sham muscle fibers from both WT and miPLA_2_βKO mice (Fig. 5, A and B). In contrast, membrane excitability was profoundly impaired in WT denervated fibers, with depolarization-induced force responses reduced to <5% of the maximum force (Fig. 5, A and B). Notably, deletion of iPLA_2_β significantly preserved excitability in denervated muscle fibers, maintaining the depolarization-induced force response to ∼30% of maximum Ca^2+^-activated force (Fig. 5, A and B). To investigate the mechanisms underlying this partial preservation of excitability, we analyzed the protein content of Na^+^/K^+^–adenosine triphosphatase (ATPase) (NKA), the primary regulator of sarcolemmal membrane potential. The NKA α-subunit exists in two major isoforms: NKAα1, which is ubiquitously expressed in all tissues, and NKAα2, which is skeletal muscle–specific and the predominant isoform in muscle tissue. Western blot analysis revealed no significant change in NKAα1 levels across all experimental groups (fig. S2). However, the amount of NKAα2, the most abundant NKA α-subunit in skeletal muscle, was significantly reduced in denervated muscles of both WT (∼64% decrease) and miPLA_2_βKO (∼40% decrease) mice (Fig. 5, C and D). The reduction in NKAα2 expression was significantly attenuated in miPLA_2_βKO muscles compared to WT, suggesting that preservation of NKAα2 expression may underlie the improved membrane excitability observed in miPLA_2_βKO denervated fibers.

### iPLA_2_β deletion modulates the expression of calcium-dependent signaling proteins altered by denervation

**Fig. 6.**
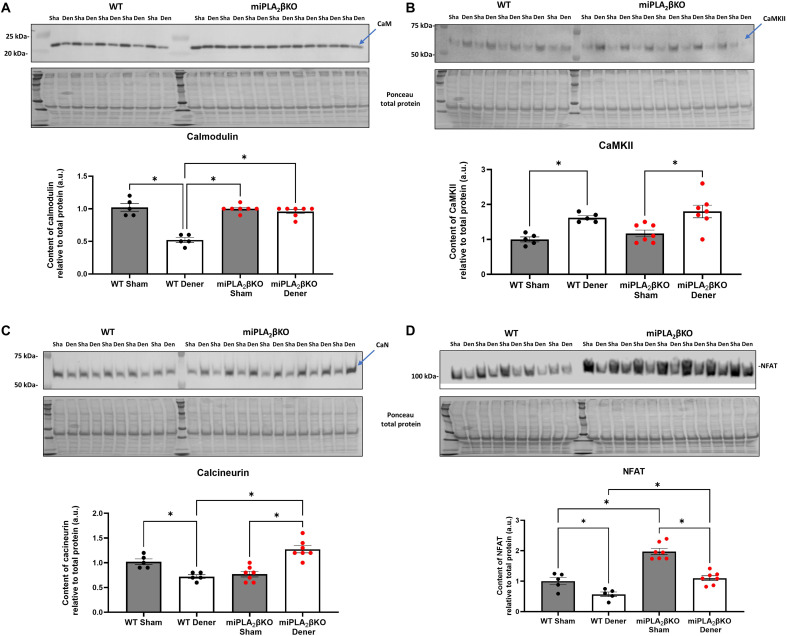
Detection of reactive proteins related to calcium homeostasis. Representative Western blot images and pooled data for (**A**) CaM, (**B**) CaMKII, (**C**) CaN, and (**D**) NFAT. * denotes significant difference between labeled groups (*P* < 0.05, two-way ANOVA with Tukey’s post hoc correction). *n* = 5 to 7 animals. Data are presented as mean values ± SEM from *n* = 5 to 7 mice.

The sensitivity to hyperactivation of SOCE by denervation may also reflect broader changes in calcium signaling pathways that are critical for muscle mass regulation. To investigate this possibility and the role of iPLA_2_β, we analyzed the expression of key Ca^2+^-related proteins in whole GTN muscle homogenates by Western blot analysis. Specifically, we measured levels of calmodulin (CaM), Ca^2+^/calmodulin-dependent protein kinase II (CaMKII), calcineurin (CaN), and nuclear factor of activated T cells (NFAT), all of which are directly or indirectly regulated by intracellular Ca^2+^ dynamics and play critical roles in muscle remodeling. CaM, a ubiquitous calcium-binding messenger protein, showed a 48% reduction in WT denervated muscles compared to sham (Fig. 6A). This reduction was fully rescued by iPLA_2_β deletion, which maintained CaM levels to those seen in sham muscles, indicating improved maintenance of Ca^2+^ signaling under denervation stress (Fig. 6A). CaMKII, a serine/threonine kinase activated by the Ca^2+^-CaM complex and involved in Ca^2+^ reuptake and signaling, was up-regulated in denervated muscles from both WT and miPLA_2_βKO mice (Fig. 6B). This suggests a compensatory response to altered Ca^2+^ homeostasis that is not directly modulated by iPLA_2_β. CaN, a Ca^2+^-CaM–dependent phosphatase that promotes muscle growth via NFAT dephosphorylation, was significantly reduced by 28% in WT denervated muscles relative to sham controls (Fig. 6C). However, in miPLA_2_βKO mice, CaN expression was not only preserved but was significantly elevated in denervated muscles by 65% compared to miPLA_2_βKO sham controls (Fig. 6C). This finding indicates enhanced anabolic signaling potential following iPLA_2_β deletion. We also measured the expression of NFAT, a transcription factor involved in muscle development and growth, and a downstream target of CaN. NFAT levels were significantly suppressed by denervation in both WT (∼43% decrease) and miPLA_2_βKO (∼57% decrease) muscles relative to their respective sham controls (Fig. 6D). However, the overall NFAT expression was markedly higher in both sham and denervated miPLA_2_βKO muscles compared to WT counterparts. As a result, NFAT expression in miPLA_2_βKO denervated muscles was ∼2.2-fold higher than in WT denervated muscles (Fig. 6D), suggesting enhanced transcriptional activity associated with muscle maintenance and resistance to atrophy.

### iPLA_2_β deletion modulates key muscle mass regulatory proteins following denervation

**Fig. 7.**
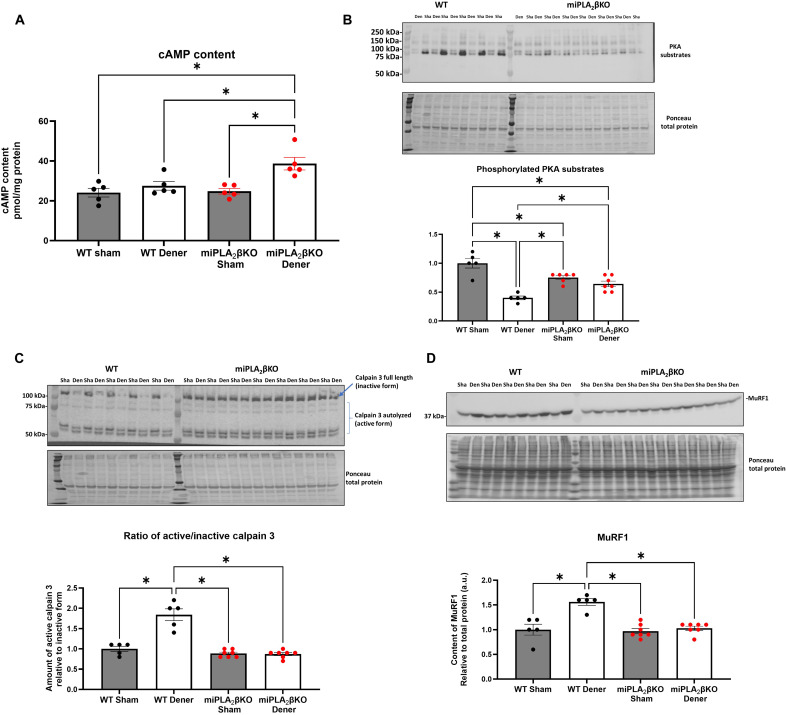
Expression of proteins related to muscle mass regulation. (**A**) ELISA analysis of cAMP content in different groups. Representative Western blot images and pooled data for (**B**) phosphorylated PKA substrates, (**C**) calpain 3 (both inactive and active forms), and pooled data for the ratio of active calpain 3 relative to its inactive form, and (**D**) MuRF1. * denotes significant difference between labeled groups (*P* < 0.05, two-way ANOVA with Tukey’s post hoc correction). *n* = 5 to 7 mice. Data are presented as mean values ± SEM.

To elucidate the molecular mechanisms underlying the preservation of muscle mass in miPLA_2_βKO mice following denervation, we examined the expression of several proteins known to regulate muscle growth and atrophy using GTN muscle homogenates. First, we quantified intracellular adenosine 3′,5′-monophosphate (cAMP) levels using an enzyme-linked immunosorbent assay (ELISA) assay. cAMP and calcium signaling are interconnected through direct cross-talk mechanisms (*20*), and the activity of cAMP-dependent protein kinase (PKA) regulates calcium channel activities, promotes muscle growth, and suppresses atrophy (*21*, *22*). Notably, cAMP levels were significantly elevated (∼56%) in denervated muscles of miPLA_2_βKO mice compared to all other groups (Fig. 7A), suggesting enhanced activation of cAMP-mediated signaling in the absence of iPLA_2_β. Next, we assessed the phosphorylation of PKA substrates. PKA substrate phosphorylation levels were markedly reduced (∼60%) in denervated muscles of WT mice relative to their sham counterparts (Fig. 7B). While PKA activity in miPLA_2_βKO denervated muscles remained lower (∼36%) than in WT sham controls, it was significantly higher (∼52%) than in WT denervated muscles and comparable to levels in miPLA_2_βKO sham muscles (Fig. 7B). These data indicate that iPLA_2_β deletion preserves cAMP-PKA signaling under denervated conditions.

We next quantified calpain 3, a muscle-specific Ca^2+^-activated cysteine protease that plays a key role in proteolytic pathways and is involved in protein degradation and muscle damage. The full-length (∼94 kDa) form of calpain 3 represents its inactive state, and upon activation by sufficient Ca^2+^, calpain 3 undergoes autolysis, producing smaller fragments. This autolytic process can be detected by Western blotting as a decrease in the 94-kDa full-length protein and the increase in the appearance of autolytic products ranging from 55 to 80 kDa. In WT denervated muscles, the level of active calpain 3 was significantly elevated by ∼84% compared to sham controls (Fig. 7C). This increase is consistent with enhanced proteolytic activity and muscle atrophy seen in denervated muscles. Notably, iPLA_2_β deletion normalized active calpain 3 levels in denervated muscles, preserving them to levels comparable to sham controls (Fig. 7C), suggesting a suppression of proteolytic signaling in denervated muscles from miPLA_2_βKO mice.

To further evaluate the impact of iPLA_2_β deletion on muscle atrophy pathways, we examined the expression of Muscle Ring-Finger Protein-1 (MuRF1), a well-established E3 ubiquitin ligase involved in the proteasome-mediated degradation of MHC and a key marker of muscle atrophy. MuRF1 levels were significantly increased (∼56%) in WT denervated muscles compared to sham but were sustained to near-baseline levels in miPLA_2_βKO denervated muscles (Fig. 7D), suggesting a protective effect against muscle protein degradation.

### iPLA_2_β deletion alters the muscle oxylipin profile

**Fig. 8.**
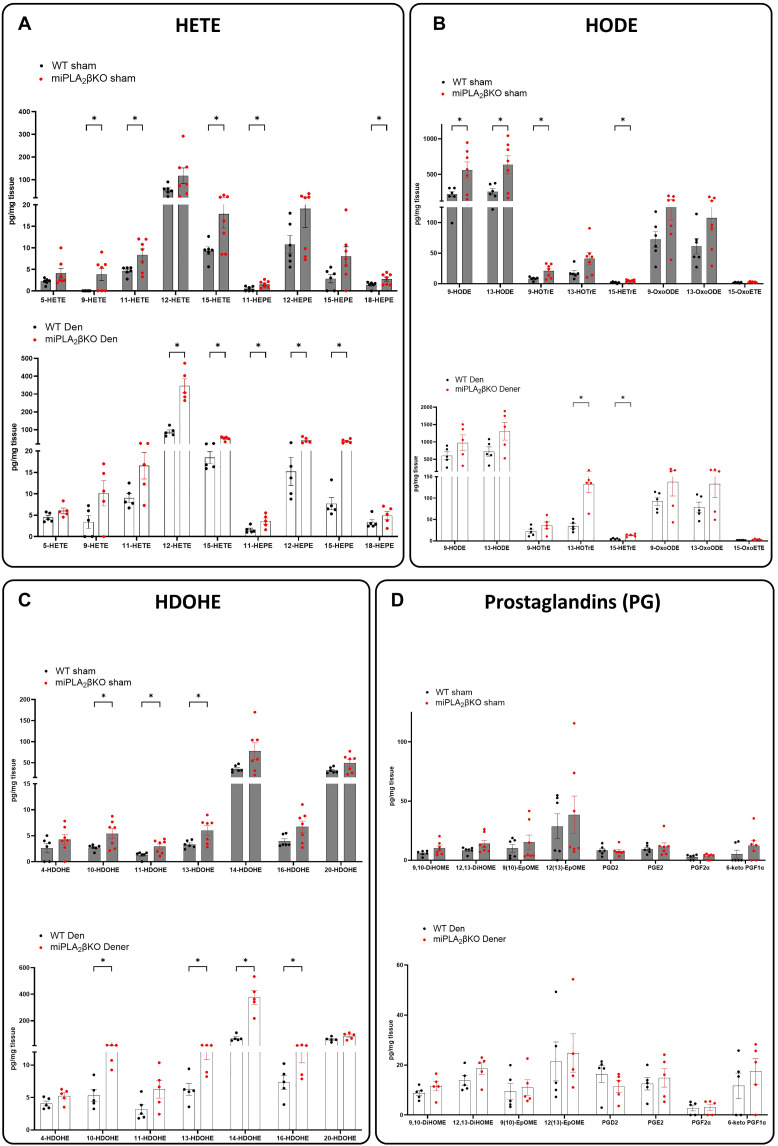
Lipidomic analysis of oxylipin contents. Quantification of oxylipin species in sham (shaded bar) and denervated (open bar) GTN muscle from WT (black symbols) and iPLA_2_βKO (red symbols) mice. Oxylipin classes analyzed include (**A**) HETEs, (**B**) HODEs, (**C**) HDOHEs, and (**D**) PGs. * denotes significant difference between labeled groups (*P* < 0.05, unpaired *t* test). *n* = 5 to 7 mice. Data are presented as mean values ± SEM.

Because loss of iPLA_2_β is expected to influence membrane phospholipid remodeling and downstream lipid signaling, we quantified the muscle oxylipin profile in sham and denervated muscles to determine the impact of iPLA_2_β deletion. Oxylipin species were grouped into four groups, hydroxyeicosatetraenoic acids (HETEs; Fig. 8A), hydroxyoctadecadienoic acids (HODEs; Fig. 8B), hydroxydocosahexaenoic acids (HDOHEs; Fig. 8C), and prostaglandins (PGs; Fig. 8D). Denervation significantly increased the abundance of nearly all measured oxylipin species compared to sham muscles in both WT and iPLA_2_βKO muscles (fig. S12). In sham muscles, iPLA_2_β deletion significantly elevated multiple oxylipins, including 9-, 11-, and 15-HETEs; 11- and 18-HEPEs (hydroxy-eicosapentaenoic acid); 9- and 13-HODEs; 9-HOTrE (hydroxyoctadecatrienoic acid); 15-HETrE (hydroxy-eicosatrienoic acid); and 10-, 11-, and 13-HDOHEs (Fig. 8, A to C, top). Notably, iPLA_2_β deletion did not attenuate the denervation-associated accumulation of oxylipins, and oxylipin abundance remained markedly elevated in iPLA_2_βKO denervated muscles, including 12- and15-HETEs; 11-, 12-, and 15-HEPEs; 13-HOTrE; 15-HETrE; and 10-, 13-, 14-, and 16-HDOHEs (Fig. 8, A to C, bottom). Collectively, these findings demonstrate that iPLA_2_β deletion significantly remodels the muscle oxylipin profile under basal conditions, while denervation induces a broad and robust increase in oxylipin production that occurs largely independent of iPLA_2_β signaling pathway.

### Transcriptomic profiling reveals altered gene regulation and the impact of iPLA_2_β deletion on cAMP and calcium signaling pathways in denervated muscle

**Fig. 9.**
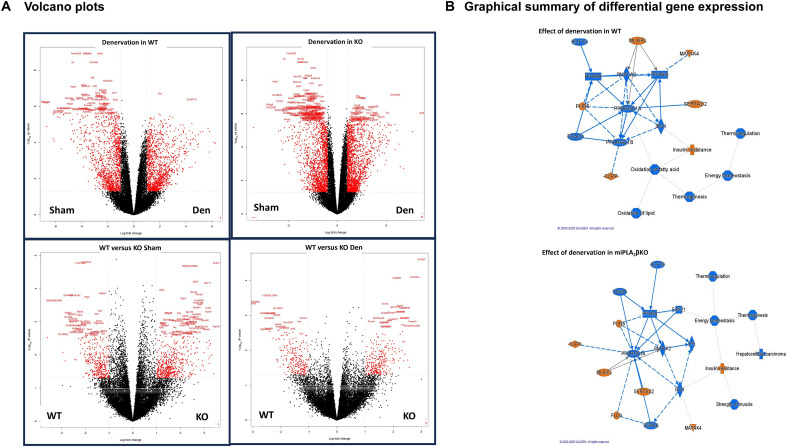
Differential gene expression after denervation between groups. (**A**) Volcano plots show genes differentially expressed by denervation in WT and KO mice (top) and by KO in sham and denervated legs (bottom). Each point represents a single gene. The *x* axis indicates the log_2_ fold change (log_2_FC) in gene expression, and the *y* axis shows the –log_10_ of the adjusted *P* value (FDR-corrected). Genes with significant expression changes are plotted in red, whereas nonsignificant genes are shown in gray. Labeled points mark the most significantly differentially expressed genes based on both fold change and statistical significance. These plots illustrate the distinct transcriptional profiles among groups and highlight key genes potentially contributing to the observed differences. Denervation has a strong effect, resulting in over 1000 differentially expressed genes, whereas the KO effect is more subtle. The denervation response is skewed toward reduced expression, with the strongest statistical significance observed for down-regulated genes. (**B**) Graphical summaries illustrate the key biological functions, canonical pathways, and upstream regulators inferred from genes differentially expressed after denervation in WT and KO mice, analyzed using IPA (QIAGEN Inc.). Orange shapes indicate predicted activation, and blue shapes indicate predicted inhibition, based on IPA *z* scores, with color intensity reflecting the predicted degree of regulation. Gray elements represent entities with no prediction. Solid lines denote direct relationships, while dashed lines indicate indirect interactions. Predictions are derived from the QIAGEN Knowledge Graph database using machine learning algorithms to prioritize and connect entities [data were analyzed through the use of QIAGEN IPA; (*80*)].

To shed further light on the potential molecular mechanisms underlying the protective effect of iPLA_2_β deletion in denervation-induced muscle atrophy, we conducted bulk RNA sequencing (RNA-seq) analysis comparing GTN muscle from WT and miPLA_2_βKO sham and denervated legs. In addition, based on our findings showing changes in cAMP and calcium signaling pathways, we performed a focused transcriptomic screen of genes involved in cAMP and calcium signaling using a targeted gene array. RNA-seq pathway analysis was performed using Ingenuity Pathway Analysis (IPA) software, and genes with fold change >2 or <−2 and false discovery rate (FDR) < 0.05 were considered significantly regulated. This analysis revealed 637 genes with altered expression in response to denervation in WT mice, while almost twice as many genes were altered in iPLA_2_βKO mice compared to the WT mice after denervation (1035 genes). The volcano plot in Fig. 9A illustrates the relative gene expression changes among groups. Both WT and miPLA_2_βKO muscle have more genes with reduced expression than elevated expression. To reveal the impact of iPLA_2_β deletion in muscle before denervation, we compared gene expression in the sham leg of WT and iPLA_2_βKO mice. A total of 383 genes showed differential expression in the sham leg of miPLA_2_βKO mice compared to WT mice. A total of 228 genes were differentially expressed between WT and miPLA_2_βKO mice in the denervated leg. The IPA-generated graphical summary analysis shown in Fig. 9B indicates a similar response in general to denervation in WT and miPLA_2_βKO mice that reveals inhibition of peroxisome proliferator–activated receptor α (PPARα), PPARGC1a (PGC1α), PNPLA2, IRS1, TEAD1, and KLF15 and activation of several key modulators including MLXIPL, MAP4K4, PLIN5, CLPP, and SERTAD2 (SERTA domain containing 2). However, there are also a few notable differences. In particular, there is an inhibition of FGF21 and ESRRA expression and activation of folliculin (FLCN) that only occurs in the iPLA_2_βKO muscle following denervation.

We also performed differential expression analysis from the bulk RNA-seq data. Figure S9A shows genes that are up-regulated threefold or more in both WT and miPLA_2_βKO muscle after denervation. There is a tendency for the up-regulation to be higher in the miPLA_2_βKO group for several of these genes. Similarly, fig. S9B shows 20 genes that are down-regulated at least fourfold in both WT and miPLA_2_βKO mice. Figure S9 (C and D) show genes that are up-regulated at least threefold or down-regulated at least fourfold in either WT or miPLA_2_βKO alone. Of particular note, the number of genes up-regulated in the miPLA_2_βKO alone after denervation is much greater than in the WT mice.

**Fig. 10.**
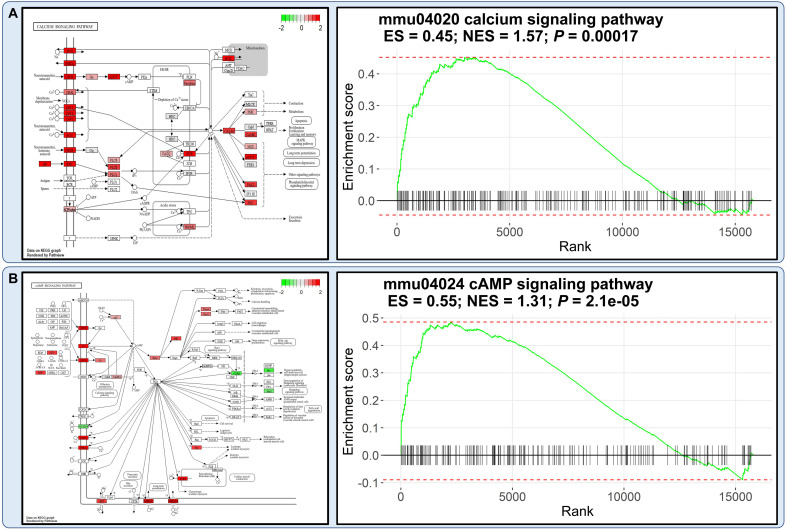
Bulk RNA-seq GSEA analysis reveals that calcium signaling and cAMP pathways are significantly altered by iPLA_2_βKO. KEGG pathway maps (left) and enrichment plots (right) for two pathways significantly enriched in differentially expressed genes following iPLA_2_β knockout, as identified using Gene Set Enrichment Analysis (GSEA) testing method. (**A**) Calcium signaling pathway and (**B**) cAMP signaling pathway. KEGG pathway maps highlighting differentially expressed genes after KO. The pathway map was retrieved from the KEGG database and annotated with gene expression data. Differentially expressed genes from the GSEA pathway leading-edge subset are overlaid on the map, with up-regulated genes shown in red and down-regulated genes in green. Color intensity reflects the magnitude of differential expression. GSEA enrichment plot for each pathway shows the enrichment of the pathway gene set in the ranked list of genes differentially expressed following iPLA_2_β knockout. The green curve represents the running enrichment score (ES) calculated as the analysis walks down the ranked gene list. The vertical black lines indicate the positions of genes from the gene set within the ranked list. The peak of the ES curve reflects the point of maximum enrichment. The normalized enrichment score, the nominal *P* value, and FDR *q* value reported on the graph assess enrichment statistical significance. Positive Nestin (NES) scores indicate that both pathways show increased activation following the knockout (KO), with their leading-edge subsets predominantly composed of overexpressed genes. The two pathway-related visualizations were generated using Ingenuity (left) and the Bioconductor package (right), respectively.

Kyoto Encyclopedia of Genes and Genomes (KEGG) pathway analysis based on differentially expressed genes are shown in Fig. 10 (A and B) for WT versus miPLA_2_βKO sham muscles. As shown in red, a number of genes are elevated in expression including RyR, inositol 1,4,5-trisphosphate receptor (IP3R; an intracellular Ca^2+^ channel on the SR membrane that releases Ca^2+^ in response to IP3 signaling), plasma membrane Ca^2+^-ATPase (PMCA; a membrane pump expelling Ca^2+^ from the cytoplasm to the extracellular space), and Na^+^-Ca^2+^ exchanger (NCX; a plasma membrane transporter that moves Ca^2+^ out of the cell in exchange for Na^+^). These changes support our findings that iPLA_2_β deletion enhances Ca^2+^ regulatory mechanisms and contributes to improved Ca^2+^ homeostasis following denervation. To further assess and independently confirm these pathway-level changes, we also performed a targeted gene expression analysis for these two pathways using a PCR Gene Array (QIAGEN). The main results of this screen are presented in fig. S10. Over 85 targeted genes were analyzed by real-time PCR. As shown in fig. S10A, four genes (Amd, Calm1, Slc18a1, and Per1) were up-regulated in sham muscle from miPLA_2_βKO mice compared with sham WT muscle. Figure S10B shows two genes (Egr1 and Calb1) that are up-regulated in denervated muscle from miPLA_2_βKO mice. Figure S10 (C and D) shows the effect of denervation in WT (fig. S10C) and miPLA_2_βKO (fig. S10D) mice. It is notable that the impact on gene expression is higher in the miPLA_2_β KO mice and that more genes are up-regulated than down-regulated.

## DISCUSSION

**Fig. 11.**
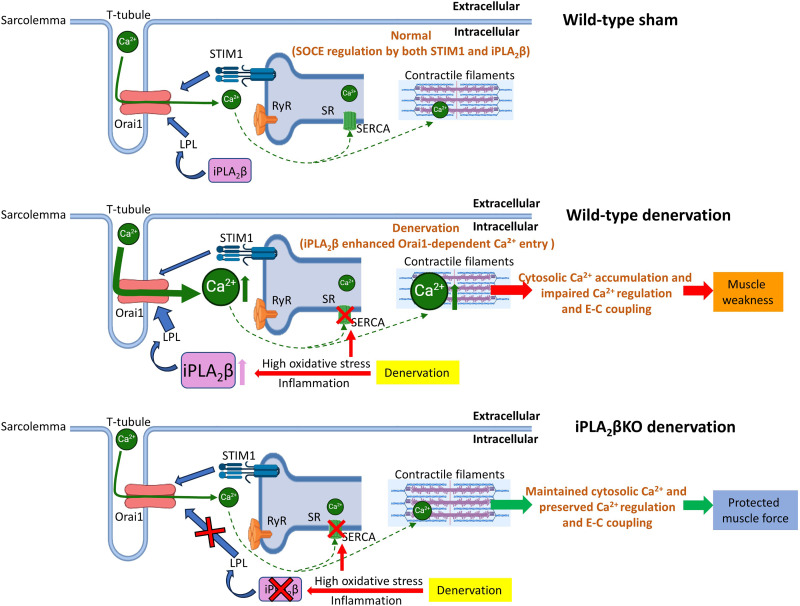
Schematic summary illustrating the role of iPLA_2_β in regulating SOCE activity and the overall Ca^2+^ homeostasis. In WT sham muscles, SOCE is normally activated through canonical STIM1 and Orai1 interaction and also regulated by iPLA_2_β activity. Following denervation, iPLA_2_β expression is up-regulated, leading to increased production of LPLs, which in turn hyperactivate Orai1 channels and enhance overall SOCE activity. This heightened calcium influx contributes to cytosolic Ca^2+^ accumulation and disrupts excitation-contraction (E-C) coupling, primarily due to impaired SERCA function under conditions of elevated oxidative stress. Knockout of iPLA_2_β attenuates SOCE activity, thereby reducing cytosolic Ca^2+^ accumulation and preserving E-C coupling in denervated muscles. Although SERCA activity remains impaired, the reduction in calcium overload ultimately leads to improved muscle force production in denervated muscles. Created in BioRender: K. Humphries (2026); https://BioRender.com/0e2jrsx.

Our study identifies a previously unrecognized role for iPLA_2_β in modulating SOCE and calcium homeostasis in skeletal muscle, particularly under denervation conditions that mimic aspects of sarcopenia. Using a miPLA_2_βKO model, we demonstrate that iPLA_2_β negatively regulates canonical STIM1-Orai1 coupling, thereby altering the mode of SOCE activation. While STIM1-dependent SOCE is the primary mechanism for Ca^2+^ influx following SR Ca^2+^ depletion (*13*–*15*), elevated iPLA_2_β expression after denervation is associated with enhanced SOCE kinetics and altered regulation of the STIM1-Orai1 pathway. In the setting of impaired Ca^2+^ handling caused by denervation-associated oxidative stress (*3*, *23*), this aberrant SOCE promotes cytosolic Ca^2+^ overload and muscle dysfunction. Deletion of iPLA_2_β restores STIM1-Orai1 complex formation, normalizes SOCE, and significantly improves muscle mass and contractile force during denervation (Fig. 11), establishing iPLA_2_β as a key link between lipid signaling and Ca^2+^ dysregulation in muscle and a potential target for mitigating sarcopenia. Consistent with our previous work (*2*, *4*), denervation in WT mice reduced muscle mass and strength while markedly increasing iPLA_2_β expression, suggesting a stress responsive to nerve injury, similar to cPLA_2_ up-regulation (*4*). iPLA_2_β deletion preserved muscle mass and, more notably, maintained force generation by ∼50% despite modest effects on mass (∼5%), underscoring the functional relevance of iPLA_2_β-mediated Ca^2+^ dysregulation and highlighting its potential as an intervention target for denervation-induced muscle weakness.

Fiber typing analyses confirm that denervation induces significant atrophy at the fiber level, particularly in fast-twitch glycolytic fibers (types IIB and IIX), consistent with their known susceptibility to neurogenic atrophy compared to oxidative fibers (*1*, *24*). This response was not prevented by miPLA_2_β deletion, as both WT and miPLA_2_βKO mice exhibited marked reductions in type IIB and IIX fiber CSA, while total fiber number was preserved, indicating atrophy rather than fiber loss (*25*). Denervated WT muscles showed an increased proportion of type IIB fibers, likely reflecting a relative shift in fiber composition rather than true hyperplasia, potentially driven by selective atrophy or fiber-type transitions of non-IIB fibers. The absence of this shift in miPLA_2_βKO muscles, together with the selective reduction in type I fiber CSA observed only in WT mice, suggests a modest role for iPLA_2_β in regulating oxidative fiber remodeling after denervation. Given that PLA_2_-dependent mitochondrial reactive oxygen species (ROS) production contributes to muscle oxidative stress and atrophy (*4*, *26*–*28*) and that type I fibers have higher mitochondrial content (*24*, *29*), elevated iPLA_2_β expression following denervation may preferentially exacerbate oxidative stress in slow-twitch fibers, whereas iPLA_2_β deletion may help preserve redox homeostasis and limit oxidative fiber atrophy.

SOCE is a fundamental mechanism for maintaining intracellular Ca^2+^ homeostasis across eukaryotic cells (*15*) and plays a critical role in skeletal muscle by supporting cytosolic and SR Ca^2+^ balance, excitation-contraction coupling, mitochondrial Ca^2+^ handling, and energy production (*30*–*35*). In muscle, SOCE is rapidly activated by localized depletion of junctional SR Ca^2+^ rather than global SR depletion, enabling immediate Ca^2+^ replenishment during sustained or repeated contractions to avoid net loss of Ca^2+^ from the fiber (*36*–*38*). In this study, SOCE activity was markedly elevated in denervated muscle fibers. While enhanced SOCE is not inherently pathological, its impact depends on the integrity of intracellular Ca^2+^ handling. Consistent with prior reports (*1*, *3*, *39*), denervation impaired SERCA function in both WT and miPLA_2_βKO muscles, which would be expected to reduce SR Ca^2+^ loading rather than directly elevate resting cytosolic Ca^2+^, as resting [Ca^2+^] is primarily governed by plasma membrane Ca^2+^ fluxes (*40*). The observed Ca^2+^ accumulation is more likely driven by sustained SOCE activity or Oria1 activation due to elevated iPLA_2_β activity. In this setting, reduced SERCA activity may exacerbate Ca^2+^ dysregulation by limiting SR Ca^2+^ sequestration during ongoing Ca^2+^ entry. Supporting this interpretation, denervated iPLA_2_βKO muscles exhibited increased CSQ content (fig. S3B), suggesting an enhanced SR Ca^2+^ storage capacity that may partially compensate for SERCA dysfunction. Meanwhile, largely unchanged PMCA activity measured in this study (fig. S5, B and C) suggests that Ca^2+^ extrusion is insufficient to counterbalance increased influx. We also observed a trend toward a reduced Ca^2+^ reloading rate in miPLA_2_βKO fibers; however, this difference did not reach statistical significance, likely due to the limited number of fibers analyzed. Despite the lack of a significant change in measured activity, PMCA protein content was significantly reduced in denervated muscle (fig. S5D), indicating a potential impairment of Ca^2+^ extrusion capacity at the level of protein abundance. Notably, deletion of iPLA_2_β restored PMCA protein levels in denervated muscle, suggesting that iPLA_2_β contributes to denervation-induced alterations in PMCA expression and Ca^2+^ handling. Our findings establish, for the first time, a functional role for iPLA_2_β in regulating SOCE activation in skeletal muscle. iPLA_2_β promotes SOCE via LPL-mediated activation of Orai1, bypassing the requirement for STIM1 and enabling rapid Ca^2+^ entry to occur in cell types with narrower ER–plasma membrane junctions (typically 4 to 6 nm in most cell types) as reported previously (*10*, *11*). Although skeletal muscle has sufficient SR–t-tubule junctional spacing (12 to 14 nm) to support canonical STIM1-Orai1 coupling as demonstrated here and reported by others (*16*), we demonstrate that iPLA_2_β negatively regulates this interaction, as deletion of iPLA_2_β markedly enhances STIM1-Orai1 complex formation. STIM1 and Orai1 proteins are not exclusively localized to the t-tubule system. Recent studies indicate that STIM1 can also reside in nonjunctional membranes, including the nuclear envelope, where it can influence nuclear-cytosolic signaling (*41*). These nontubular pools of STIM1/Orai1 may contribute to the global interactions detected in our binding data without directly participating in SOCE at t-tubule–SR junctions. This limitation underscores the need for more spatially resolved approaches to more precisely define STIM1-Orai1 interactions in specific membrane domains. Last, iPLA_2_β deletion normalized the denervation-induced hyperactivation of SOCE and significantly reduced cytosolic Ca^2+^ accumulation, despite persistent SERCA dysfunction, identifying iPLA_2_β as a critical modulator of pathological Ca^2+^ influx in denervated skeletal muscle.

Given the central role of SOCE in intracellular Ca^2+^ homeostasis, we extended our analysis to additional SR and sarcolemmal Ca^2+^-handling and signaling proteins. RyR1, which regulates both excitation-contraction coupling and SOCE, promotes cytosolic Ca^2+^ accumulation when destabilized and leaky (*15*, *30*, *32*, *33*). iPLA_2_β deletion reduced RyR1 abundance while increasing calstabin association, resulting in an elevated calstabin-to-RyR1 ratio consistent with enhanced RyR stabilization and a reduced likelihood of SR Ca^2+^ leak, which likely contributes to normalized SOCE in denervated miPLA_2_βKO muscle. In addition, following denervation, increased RyR-mediated SR Ca^2+^ leak may reduce SR terminal Ca^2+^ content and activate canonical STIM1-dependent SOCE (*32*). In this context, our data suggest that the role of iPLA_2_β is likely to represent a modulatory mechanism that enhances the canonical STIM1-dependent SOCE activity. Our findings that STIM1 inhibition suppresses SOCE in both WT and KO fibers, whereas iPLA_2_β inhibition selectively reduces the augmented SOCE in denervated WT fibers, support this interpretation. Nevertheless, we cannot exclude the possibility that altered SR Ca^2+^ leak contributes to the observed SOCE activation at rest. Future studies combining RyR inhibition with simultaneous assessment of SOCE within the same fiber will be important to further dissect the relative contributions of SR Ca^2+^ leak and iPLA_2_β-dependent regulation.

Excess cytosolic Ca^2+^ disrupts membrane excitability by altering the function of ion transporters and channels, including NCX, NKA, and Ca^2+^-activated K^+^ channels (*42*–*47*). Accordingly, denervated WT fast-twitch fibers exhibited a near-complete loss of depolarization-induced force, whereas miPLA_2_β deletion partially sustained excitability and force generation, coinciding with preservation of the muscle-specific NKAα2 subunit. A minimum fiber diameter may be required for optimal depolarization, and atrophied fiber size following denervation could influence excitability (*44*). However, because denervation induced a similar reduction in fiber size in both WT and KO muscles, the enhanced depolarization-induced force in KO fibers cannot be attributed to fiber diameter. Consistently, whole-muscle measurements under electrical stimulation showed greater specific force in denervated KO muscle, supporting a functional improvement independent of fiber size. Beyond excitability, elevated Ca^2+^ activates proteolytic and signaling pathways that regulate muscle mass. Denervated WT muscle showed increased calpain 3 autolysis, indicative of heightened Ca^2+^-dependent proteolysis (*48**–**50*), whereas miPLA_2_βKO muscle maintained inactive calpain 3, consistent with improved Ca^2+^ handling. We further identified dysregulation of CaM, which inhibits iPLA_2_β in its Ca^2+^-bound form (*10*, *11*). Although CaM protein expression was altered, the functional implications are complex, as total CaM levels do not necessarily reflect the amount of Ca^2+^-bound CaM (Ca-CaM), the active signaling form. Given the elevated resting cytosolic Ca^2+^ in denervated fibers, Ca-CaM formation may be maintained or even enhanced despite reduced total CaM expression. Notably, Ca-CaM inhibits iPLA_2_ activity (*10*, *11*), suggesting a potential interaction between these pathways. In denervated WT muscle, the combination of reduced CaM expression and increased iPLA_2_β content would be expected to favor enhanced iPLA_2_β activity by reducing inhibitory regulation. Although elevated Ca^2+^ may partially offset this effect, the relative decrease in CaM abundance compared to increased iPLA_2_β levels likely shifts the balance toward greater iPLA_2_β activation, providing a plausible mechanism for the enhanced SOCE observed under denervation conditions (*10*, *11*, *51*, *52*). Nevertheless, we did not directly assess Ca-CaM levels or downstream CaM-dependent targets such as RyR or PMCA function in a comprehensive manner. Downstream Ca^2+^-dependent signaling pathways were also differentially regulated. CaMKII, a stress-responsive kinase implicated in muscle wasting and Ca^2+^ dysregulation (*53*–*55*), was up-regulated following denervation regardless of genotype. In contrast, CaN, a prohypertrophic regulator of muscle growth and regeneration (*56*, *57*), was reduced in WT denervated muscle but preserved and even elevated in miPLA_2_βKO muscle, potentially contributing to the partial protection against denervation-induced atrophy.

In addition to Ca^2+^-dependent mechanisms, we examined the cAMP-PKA signaling pathway, another key regulator of muscle mass. cAMP and its downstream effector PKA are known to promote muscle hypertrophy, metabolism, and regeneration (*21*). In WT mice, denervation did not alter cAMP content but significantly reduced PKA activity, consistent with muscle atrophy. Although iPLA_2_β deletion did not change basal cAMP levels, RNA-seq revealed up-regulation of cAMP signaling–related genes, indicating functional modulation downstream of cAMP. Notably, denervated miPLA_2_βKO muscles exhibited elevated cAMP levels and maintained higher PKA activity compared with WT denervated muscles, which may contribute to partial muscle preservation. Because PKA-dependent phosphorylation can inhibit calpain activity (*22*), increased PKA signaling may underlie the suppression of calpain 3 and reduced proteolysis observed in miPLA_2_βKO muscles. However, blunting proteolytic pathways alone produced only modest protection against muscle loss. We further assessed denervation-induced atrophy markers. MuRF1, a muscle-specific E3 ubiquitin ligase central to proteasomal degradation (*58*, *59*), was robustly up-regulated in WT denervated muscle but restored toward baseline in miPLA_2_βKO muscles, suggesting reduced proteasomal activity. In parallel, NFAT, a CaN-dependent transcription factor involved in muscle growth (*58*, *59*), remained significantly higher in miPLA_2_βKO muscles under both sham and denervated conditions. NFAT levels in miPLA_2_βKO denervated muscle were more than twofold higher than in WT, consistent with elevated CaN expression and sustained CaN-NFAT signaling. Together, these data suggest that iPLA_2_β deletion partially preserves muscle mass after denervation through coordinated modulation of cAMP-PKA signaling and CaN-NFAT–dependent transcription.

Despite the established contribution of mitochondrial dysfunction and oxidative stress to denervation-induced sarcopenia, iPLA_2_β deletion did not rescue impaired mitochondrial function. Consistent with our previous findings, denervation markedly increased mitochondrial ROS production (*2*, *4*), and this elevation persisted in miPLA_2_βKO muscle, indicating sustained oxidative stress (fig. S8). In line with this, lipidomic analyses revealed a broad accumulation of oxylipins following denervation that was not attenuated by iPLA_2_β deletion, suggesting that denervation-driven lipid oxidation occurs largely independent of iPLA_2_β signaling. Supporting ongoing lipid peroxidation at the protein level and similar to what we reported before (*19*), denervation also increased the abundance of 4-hydroxynonenal (4HNE)–modified proteins in both WT and miPLA_2_βKO muscles (fig. S11), indicating persistent oxidative damage despite genetic deletion of iPLA_2_β. Notably, although mitochondrial ROS and oxylipin abundance remained elevated, miPLA_2_βKO muscles exhibited preserved mitochondrial OXPHOS complex content (fig. S8, C and D), indicating maintained mitochondrial protein stability or structural integrity without functional recovery. Together, these findings highlight a highly complex and multifactorial regulation of mitochondrial homeostasis and oxidative stress during denervation.

Bulk RNA-seq revealed extensive transcriptional remodeling in both WT and miPLA_2_βKO muscles following denervation. As summarized in Fig. 9B, denervation similarly suppressed key regulators of metabolism, mitochondrial biogenesis, and muscle regeneration in both genotypes, including PPARα, TEAD1, IRS1, KLF15, PGC1α, and PNPLA2, consistent with impaired energy homeostasis. In parallel, denervation activated stress- and metabolism-related pathways in both groups, including genes involved in lipid handling, inflammation, and mitochondrial protein quality control (e.g., MLXIPL, MAP4K4, PLIN5, CLPP, and SERTAD2). Despite these shared responses, miPLA_2_βKO muscles exhibited distinct transcriptional features following denervation. In particular, inhibition of the metabolic regulator FGF21 and ESRRA (estrogen-related receptor alpha), along with selective activation of the metabolic regulator FLCN, occurred only in miPLA_2_βKO muscle, suggesting genotype-specific metabolic reprogramming. Differential expression analysis (fig. S9) not only identified a core set of denervation-responsive genes common to both genotypes, many of which have been previously reported (*4*, *39*, *60*–*62*), but also revealed a broader and more robust gene induction unique in miPLA_2_βKO muscle. This expanded transcriptional response may contribute to the partial resistance of miPLA_2_βKO muscles to denervation-induced atrophy. Pathway enrichment further demonstrated significant overrepresentation of calcium and cAMP signaling pathways in miPLA_2_βKO muscle. Quantitative PCR (qPCR) validation (fig. S10) confirmed that while denervation up-regulated calcium- and cAMP-related genes in WT muscle, miPLA_2_βKO muscles displayed a distinct reprogrammed expression profile, consistent with enhanced calcium homeostatic control. Collectively, these findings identify iPLA_2_β as a key mediator of denervation-induced muscle dysfunction by linking SOCE-driven calcium overload to impaired excitation-contraction coupling and maladaptive transcriptional reprogramming, positioning iPLA_2_β as a potential intervention target for denervation-associated muscle weakness.

In conclusion, this study identifies iPLA_2_β as a previously unrecognized regulator of skeletal muscle physiology through its modulation of SOCE. We demonstrate that muscle SOCE is controlled by a dual mechanism involving STIM1 and iPLA_2_β, with elevated iPLA_2_β impairing canonical STIM1-Orai1 signaling. During denervation, increased iPLA_2_β drives SOCE hyperactivation and cytosolic Ca^2+^ overload, contributing to muscle atrophy and weakness. Muscle-specific deletion of iPLA_2_β normalizes SOCE, preserves Ca^2+^ homeostasis, and partially protects muscle mass and contractile function. Collectively, these findings establish iPLA_2_β as a key mediator of the muscle response to denervation and highlight iPLA_2_β-dependent signaling as a potential intervention target for modulating muscle dysfunction associated with loss of innervation, with potential implications to aging-related muscle decline.

It is necessary to acknowledge that the t-system Rhod-5N signal was not calibrated to absolute [Ca^2+^]t-system for individual fibers. Because Rhod-5N fluorescence intensity can vary with dye loading and local optical conditions, comparisons of absolute fluorescence values across fibers must be interpreted with caution. Our analysis focused on the kinetics of SOCE-related Ca^2+^ movements rather than absolute t-system [Ca^2+^]. Nevertheless, we cannot exclude the possibility that differences in baseline t-system Ca^2+^ levels between groups may influence the driving force for Ca^2+^ flux and thereby contribute to the observed differences. Future studies incorporating in situ calibration of Rhod-5N signals would further strengthen quantitative interpretation of t-system Ca^2+^ dynamics. Another limitation of this study lies in the use of the denervation mouse model. Although we used this model to induce elevated lipid oxidation in a short term, it may not fully recapitulate the complex features of natural aging. As we reported previously (*2*, *19*), surgical denervation represents a severe condition in which skeletal muscle undergoes nearly complete loss of innervation and acute injury responses. This differs substantially from aging, where only ∼30% of fibers experience partial denervation accompanied by mild and chronic inflammation (*1*, *3*, *5*). Consequently, the status of SOCE, as well as the role of iPLA_2_β, may not be identical between denervated and naturally aged muscle. To address this, we have initiated ongoing studies to investigate the function of iPLA_2_β and the regulation of SOCE in skeletal muscle from naturally aged mice.

## MATERIALS AND METHODS

### Animals and the generation of the muscle-specific iPLA_2_β knockout mice

The mice used in this study were maintained on a C57BL/6J background. iPLA_2_βflox mice were purchased from the Jackson Laboratory (strain no. 027806) and crossed with mice expressing the Cre recombinase gene driven by the human alpha-skeletal actin (HSA or *ACTA1*) promoter (strain no. 006149) to generate miPLA_2_βKO mice (fig. S1). All mice were caged in a pathogen-free environment with free access to standard chow and water. Mice were group-housed and maintained on a 14-hour light/dark cycle (light from 06:00 to 20:00). Both male and female mice were used in this study, and no differences were observed between sexes. Denervation surgery and measurements were performed at 6 months of age in both WT (homozygous flox/Cre negative) and (homozygous flox/Cre positive) miPLA_2_βKO mice. The Institutional Animal Care and Use Committee at Oklahoma Medical Research Foundation (Oklahoma City, OK, USA) approved all procedures (protocol no. 24-40).

### Sciatic nerve transection surgeries (denervation surgery)

Sciatic nerve transection and sham surgeries were performed on 6- to 8-month-old WT and miPLA_2_βKO mice as previously described (*4*). Mice were anesthetized in an induction chamber with isoflurane (2 to 3%) and maintained under anesthesia via a nose cone. The skin from the sciatic notch to the knee on the lateral thigh and buttock was shaved and disinfected with 100% ethanol followed by 2% chlorhexidine. A small incision (∼1 cm) was made from the sciatic notch to expose the sciatic nerve. For the denervation procedure, the nerve was transected and a 5-mm segment was removed. To prevent nerve regrowth, the cut ends were folded back and sutured. On the contralateral limb, the sciatic nerve was similarly exposed but left intact to serve as a sham-operated control. Incisions on each leg were closed using 5-0 polydioxanone sutures and surgical glue. Mice were placed on a warming pad until fully recovered from anesthesia. Postoperative pain was managed with ketoprofen (9.5 mg/kg, in saline) administered once daily for 3 days. Mice were euthanized, and tissues were collected 7 days after surgery.

### Assessment of muscle contractile properties

Contractile properties were measured in the EDL muscle as previously described (*6*). Briefly, the EDL muscle was excised from tendon to tendon and suspended on a dual-mode muscle lever system (300C-LR, Aurora Scientific Inc., Aurora, ON, Canada) using a hook in Krebs buffer (in millimolar: 137 NaCl, 5 KCl, 1 MgSO_4_, 1 NaH_2_PO_4_, 24 NaHCO_3_, and 2 CaCl_2_). The hind limb EDL muscle was chosen for analysis due to the well-documented response of this muscle to oxidative stress related to aging and other muscle diseases (*63*). The muscle was placed at optimal length and allowed 20 min for thermoequilibration at 32°C. A supramaximal current (600 to 800 mA) of 0.2-ms pulse duration was delivered through a stimulator (701C, Aurora Scientific Inc.) for twitch force production. Maximum tetanic force was measured with 300-ms duration train of 200-Hz stimuli. We recorded and analyzed data using commercial software (DMC and DMA, Aurora Scientific). Specific force (in newtons per square centimeter) of EDL muscles was calculated using the ratio of fiber length to muscle length as published previously (*63*). EDL muscle was used for contractility assays, whereas GTN muscle was used for other biochemical assays to avoid any effect from stimulation.

### Single muscle fiber dissection and buffers used in single fiber manipulations

After euthanasia, the EDL muscle was dissected from each mouse and used to isolate single, skinned fast-twitch muscle fiber segments. As described previously (*43*), following dissection, the EDL muscle was pinned at its resting length on a Sylgard layer under paraffin oil at room temperature (RT) and maintained on an ice pack (∼10°C) to keep it cool. Single muscle fiber segments were dissected under a light microscope, and the sarcolemma was removed by mechanically skinning the fiber segment using fine forceps, as described previously. Then, the skinned fiber segment was mounted onto the muscle-station-skinned (Myotronic, Heidelberg, Germany) at the resting length (i.e., length just less than that eliciting measurable passive force), between the forceps and transducer (TR5S, Myotronic) provided with the station. Sarcomere length was then measured at the beginning by the laser diffractions provided with the station following the mechanisms described before (*64*), and there is no difference observed across different groups (all sarcomere length is around 2.5 μm). At the end of the experiment, the fiber type of each skinned single fiber was determined by exposing it to a solution at pSr 5.2 (see below). Type II fibers generated minimal force under these conditions, while type I fibers produced more than 80% of their maximum force (*42*, *43*, *65*). All fibers examined in this study were fast-twitch fibers unless otherwise stated.

All chemicals used for single fiber manipulations were from Sigma-Aldrich (St. Louis, MO, USA) unless otherwise stated. Two heavily Ca^2+^-buffered solutions were used for examining the properties of the contractile apparatus: “relaxing solution,” containing (in millimolar) 126 K^+^, 36 Na^+^, 1 free Mg^2+^ (10.3 total Mg^2+^), 90 Hepes, 50 EGTA, 8 adenosine 5′-triphosphate (ATP), and 10 creatine phosphate (pH 7.10), with pCa (= −log_10_[Ca^2+^]) > 9 and an osmolality of 295 ± 10 mosmol/kg H_2_O; and “maximal Ca^2+^-activating solution,” which was very similar but with 50 mM CaEGTA (at pCa ∼ 4.7) instead of EGTA, and with 8.1 total Mg^2+^ to keep the free [Mg^2+^] at 1 mM (*42*, *43*, *65*). For assessing fiber type as mentioned above, a strontium-based solution at pSr 5.2 was made similarly to “maximal Ca^2+^-activating solution” but with Sr^2+^ instead of Ca^2+^ and mixing this with “relaxing solution” at a ratio of 1:7. K^+^-HDTA solution was used to examine excitation-contraction coupling (membrane excitability) and SOCE properties; it was similar to relaxing solution but with HDTA replacing EGTA, and contained (in millimolar) 126 K^+^, 36 Na^+^, 1 free Mg^2+^ (8.5 total Mg^2+^), 90 Hepes, 50 HDTA, 0.05 EGTA, 8 ATP, and 10 creatine phosphate (pH 7.10), with the free [Ca^2+^] weakly buffered at pCa ∼ 7.1 and an osmolality of 295 ± 10 mosmol/kg H_2_O. In addition, a Na^+^-HDTA solution was made similarly to the K^+^-HDTA solution by substituting all K^+^ with Na^+^, which was achieved by adjusting the pH with NaOH instead of KOH. Na^+^-HDTA solution was used to depolarize the t-tubules in skinned muscle fibers.

### Single skinned fiber manipulation

#### Measurement of membrane excitability

Membrane excitability in skinned single fast-twitch fibers was assessed by measuring force responses triggered by depolarization. Upon mechanical skinning, the t-tubular system of the muscle fiber becomes sealed. When the skinned fiber segment is placed in a standard K^+^-HDTA solution, it can repolarize due to the activity of the NKA (Na^+^/K^+^ pump) in the t-system membrane, which establishes a high sodium and low potassium ion concentration within the sealed t-system (*44*). If the t-system is sufficiently well polarized, the voltage sensors (dihydropyridine receptors) in the t-system return to an activatable state (*66*), and if the skinned fiber is subsequently transferred into the zero potassium (Na^+^-HDTA) solution, the t-system is rapidly depolarized, activating the voltage sensors, which in turn trigger Ca^2+^ release from the SR and a resultant force response (*44*). Therefore, the magnitude of the depolarization-induced force response in a skinned fiber—expressed relative to its maximum Ca^2+^-activated force—serves as an indicator of the t-system membrane polarization and the fiber’s excitability. Our previous studies have demonstrated that skinned fast-twitch fibers from EDL muscle exhibit robust depolarization-induced responses, whereas slow-twitch fibers show considerably weaker responses (*42*, *67*). In this study, through the ionic substitution from K^+^ to Na^+^, we measured the depolarization-induced responses of fast-twitch fibers from all different groups.

#### Detection of SOCE properties in single skinned fibers

SOCE activity was assessed in single skinned fast-twitch fibers through staining Ca^2+^ in t-tubules and triggering the Ca^2+^ influx from t-tubules into cytosol by depleting the SR Ca^2+^ storage with caffeine as previously described (*15*, *68*–*70*). Briefly, intact fiber bundles were excised from EDL muscles (tendon to tendon) and were incubated in an extracellular mimicking physiological buffer containing (in millimolar) 145 NaCl, 3 KCl, 4 CaCl_2_, 2 MgCl_2_, and 10 Hepes (pH adjusted to 7.4 with NaOH). Cell impermeant Rhod-5N (Invitrogen, R14207) was added to the buffer to label the Ca^2+^ in t-tubules, and with its dissociation constant (*K*_D_) of 320 μM, Rhod-5N is well suited for detecting Ca^2+^ concentrations in the range of 100 μM to 1 mM. Following dye loading, single muscle fibers were isolated and skinned mechanically, thereby sealing the t-system as described above and trapping the dye within it. The dye-loaded fibers were then transferred to a custom-built chamber with a thin coverslip as a base and bathed in a standard K^+^-HDTA myoplasmic solution. The initial image was obtained to quantify the native T-tubule Ca^2+^ content of the fiber prior to experimental manipulation. Then, a 1 mM CaEGTA/EGTA was added to the solution to buffer-free [Ca^2+^] at ∼200 nM to ensure that the t-tubule and SR [Ca^2+^] is maintained at a higher physiological level. To deplete SR Ca^2+^ storage and activate SOCE, skinned fibers were then exposed to 30 mM caffeine (an agonist of RyRs) in a similar solution except where [Mg^2+^] was reduced to 75 μM to facilitate the rapid depletion of Ca^2+^ in the SR. In addition, 4 mM EGTA (no added Ca^2+^, yielding [Ca^2+^] < 1 nM) was included in this caffeine solution to minimize the muscle contractile movement during SR depletion.

Once SR Ca^2+^ stores were depleted, SOCE was activated in the skinned fibers, resulting in Ca^2+^ influx from the t-tubules into the cytosol. The incoming Ca^2+^ was then rapidly chelated by the 4 mM EGTA present in the bathing solution, leading to a loss of fluorescence in the dye-loaded t-tubules. This process was monitored using confocal microscopy by placing the custom-built coverslip chamber on an inverted Zeiss LSM880 microscope. Time-lapse images were captured every 5 s over a 100-s period using a ×60 water-immersion objective to track the fluorescence decay within the t-tubules. To confirm that the observed fluorescence decay reflected SOCE activity, the Ca^2+^ release–activated channel inhibitor GSK-7975A was applied to block Orai1 channels. Under these conditions, SOCE activity was markedly suppressed, and minimal Ca^2+^ fluorescence decay was detected over time (fig. S6). *F*_0_ was defined as the initial fluorescence intensity in the t-tubules prior to caffeine application. Fluorescence decay curves were generated for the first 60 s by normalizing Δ*F* (change in fluorescence) to *F*_0_ at each time point. A faster decay rate indicates higher SOCE activity in the respective fiber. The decay constant (*K*) was obtained based on the best-fit decay curve for each fiber and was plotted separately to quantify the rate of SOCE. After completion of SOCE measurements, caffeine was washed out and a Ca^2+^ loading buffer containing 200 nM free Ca^2+^ was added to the fiber to allow Ca^2+^ reuptake into the t-tubules via PMCA activity. The recovery of t-tubule fluorescence over time, reflecting Ca^2+^ reloading, was captured using the same imaging protocol. A best-fit exponential association curve was plotted by normalizing Δ*F* (change in fluorescence) to *F*_0_ (original fluorescence before SOCE measurement) at each time point, and the association rate constant (*K*_assoc_) was derived and used as an index of PMCA-mediated Ca^2+^ reuptake into the t-tubules for each group.

To directly address whether iPLA_2_β can activate Orai1 independently of STIM1, we also measured SOCE activity in both young WT and miPLA_2_βKO muscle fibers in the presence of the STIM1 inhibitor ML-9, which impedes the activation and aggregation of STIM1 molecules during SOCE activity, but has no inhibitory effect on Orai1 channels (*71*–*73*). The results demonstrate that inhibition of STIM1 significantly reduces SOCE activity in both WT and miPLA_2_βKO fibers; however, SOCE is not completely abolished (fig. S7). iPLA_2_βKO fibers exhibit a greater reduction in SOCE activity following ML-9 treatment, supporting a role for iPLA_2_β in modulating Orai1 channel activity, which may persist when STIM1 signaling is inhibited.

### Muscle fiber-type distributions and fiber size

Fiber-type composition (based on MHC expression) and fiber-type–specific CSA were quantified in the whole GTN muscles from WT and miPLA_2_βKO mice with and without denervation, as previously described (*74*). Briefly, muscles were pinned onto cork lined with Parafilm at their basal length and immediately frozen in cold isopentane. On the following day, the pinned GTN muscles were transferred to a cryostat set at −30°C (Leica CM3050, Germany). A mid-belly portion of each frozen muscle was then cut, mounted on cork using optimal cutting temperature embedding medium, and rapidly frozen in cold isopentane. Care was taken during tissue blocking to ensure that the muscle remained fully frozen. Next, serial transverse sections (10 μm) were obtained at −25°C using a rotary cryostat (Leica CM3050, Germany) and mounted on glass slides. Sections were fixed in cold acetone (−20°C) for 5 min, equilibrated to RT for 10 min, a hydrophobic pen was used to create a barrier around the section, and then washed once in phosphate-buffered saline (PBS) for 5 min. Sections were then incubated in blocking solution (BS) [5% normal horse serum in PBS with 1% Tween (PBST)] for 30 min at RT, followed by overnight incubation at 4°C with a primary antibody cocktail targeting MHC isoforms and laminin: anti-MHC I (BA-F8), anti-MHC IIa (SC-71), anti-MHC IIb (BF-F3) (Developmental Studies Hybridoma Bank, Iowa City, IA, USA), and anti-laminin (L9393, Sigma-Aldrich). Antibodies were diluted in BS at 1:250 for MHC and 1:500 for laminin. The following day, sections were washed three times (5 min each) in PBST and then incubated in BS for 30 min at RT with fluorescently conjugated secondary antibodies (Alexa Fluor, Thermo Fisher Scientific): goat anti-mouse immunoglobulin G2b (IgG2b; A21242; 1:250; excitation 647) for MHC I, goat anti-mouse IgG1 (A21127; 1:500; excitation 555) for MHC IIa, goat anti-mouse IgM (A21042; 1:500; excitation 488) for MHC IIb, and goat anti-rabbit IgG (H + L) (A31556B; 1:250; excitation 405) for laminin. After three additional PBST washes (5 min each), slides were mounted with ProLong Diamond Antifade Mountant (P36930; Thermo Fisher Scientific). Entire muscle cross-sections were imaged at 10× magnification using a Zeiss LSM710 confocal microscope with the Tile Scan function. All blocking, sectioning, staining, and imaging steps were completed within 1 week to maximize morphology preservation, image quality, and analytical reliability. Fiber size and type (MHC isoform expression) were quantified for all fibers in each section using Myovision 2.0 software (University of Kentucky) (*75*).

### SERCA activity assay

The SERCA ATPase enzyme activity was measured in GTN muscle homogenates at 37°C using a spectrophotometry assay as previously described (*1*, *3*, *39*). In brief, muscle samples were homogenized at a 1:10 ratio (w/v) in SERCA homogenizing buffer, containing (in millimolar) 250 sucrose, 5 Hepes, 0.2 phenylmethylsulfonyl fluoride, and 0.2% NaN_3_. Following centrifugation, 100 μg of protein from the supernatant was mixed with SERCA assay buffer containing (in millimolar) 200 KCl, 20 Hepes, 10 NaN_3_, 1 EGTA, 15 MgCl_2_, 5 ATP, and 10 phosphoenolpyruvate to prepare a 3-ml reaction mixture. Then, enzymes lactate dehydrogenase and pyruvate kinase (18 U/ml) and 1 mM Ca^2+^ ionophore A-23187 (C-7522; Sigma-Aldrich) were added into the mixture. This reaction mixture was then divided into aliquots and mixed with CaCl_2_ to produce eight calcium concentrations corresponding to pCa values ranging from 7.6 to 4.2, along with a Ca^2+^ free blank. The samples were then loaded into a prewarmed 37°C quartz plate. The reaction was initiated by adding 1 mM NADH (reduced form of nicotinamide adenine dinucleotide) into the mixture, and the absorbance was recorded kinetically with the following settings: temperature = 37°C, time = 30 min, λ = 340 nm, and shaking between readings. The SERCA activity was calculated using the formula

$$\text{Total ATPase rate}=\frac{\text{rate of}\;A_{340\text{nm}}\;\text{signal loss}\;}{\text{path length}\times 6.23\;{\text{mM}}^{-1}{\text{cm}}^{-1}}$$

### Measurement of cytosolic Ca^2+^ concentration

Cytosolic Ca^2+^ concentration was measured by extracting free Ca^2+^ from permeabilized muscle fiber bundles, following a protocol previously established by our laboratory (*1*, *39*). Briefly, 20 to 30 mg of GTN muscle was excised and finely teased apart along the striations in PBS to isolate smaller fiber bundles. The bundles were then transferred into a standard intracellular potassium-based K^+^-HDTA buffer containing saponin (30 μg/ml) to permeabilize the sarcolemma and 0.2% collagenase I to facilitate further separation of individual fibers. Permeabilization was carried out at 37°C for 2 hours. Following incubation, the samples were centrifuged, and the supernatant was collected and mixed with 1 μM Fura-2, a Ca^2+^-sensitive fluorescent dye (Invitrogen, F1200) (*76*). Fura-2 was precalibrated using a series of Ca^2+^ standard solutions (0 nM to 39 μM) to determine its minimum and maximum fluorescence at 380 nm ($F\frac{380}{\text{min}}$ and $F\frac{380}{\text{max}}$, respectively) (*77*). Fura-2 exhibits maximum fluorescence at 340 nm when bound to Ca^2+^, while fluorescence at 380 nm reflects its unbound state. The ratio of emissions at 340/380 nm (*R*) was used to calculate free Ca^2+^ concentration. During calibration, the minimum (*R*_min_) and maximum (*R*_max_) 340/380 ratios were also determined. The dye-loaded samples were placed into a 96-well quartz plate, and fluorescence spectra (300 to 400 nm) were acquired using a spectrophotometer (Molecular Devices, M2). The free Ca^2+^ concentration in each sample was calculated using the following equation

$${\left[{\text{Ca}}^{2+}\right]}_{\text{free}}=K_{d}^{\text{EGTA}}\times \frac{R-R_{\text{min}}}{R_{\text{max}}-R}\times \frac{F_{\text{max}}^{380}}{F_{\text{min}}^{380}}$$

where $K_{d}^{\text{EGTA}}$ = 189.1 nM (at pH 7.15 and 20°C). Fiber bundle volume was estimated based on tissue mass and the known density of mammalian skeletal muscle (1.06 g/cm^3^) (*78*). The calculated free Ca^2+^ concentration was then normalized to fiber bundle volume to determine the cytosolic [Ca^2+^] in each sample.

### Western blotting and antibody information

All Western blotting experiments were performed with tissues from GTN muscles as we described previously (*1*, *5*, *6*). In general, GTN muscle tissues were homogenized in radioimmunoprecipitation assay buffer containing (in millimolar): 50 tris (pH 7.4), 140 NaCl, 1 EDTA, 0.5 EGTA, 50 NaF, 1 NaO vanadate, 1% IGEPAL, and protease inhibitors. Total protein of homogenates was quantified with the Bio-Rad protein assay kit (Sigma-Aldrich, Poole, UK), and the same amount of protein was loaded and separated with SDS–polyacrylamide gel electrophoresis gels at certain percentages, i.e., 10 or 12%. The gel was then run at 200 V for 1 hour and wet-transferred onto 0.45-μm nitrocellulose membranes (Bio-Rad) with the conditions of 100 V, 30 min at 4°C, same as described before (*42*, *43*). After the transfer, total proteins in each lane was quantified using Ponceau staining (Sigma-Aldrich, no. P3504), the Ponceau on the membrane was removed with ddH_2_O washing, and the membrane was then blocked with 1% bovine serum albumin solution in Tris-Buffered Saline with Tween 20 (TBST) for at least 1 hour at RT. After the blocking, primary antibodies were added onto the membrane and incubated for overnight at 4°C. After the primary antibody incubation, the membrane was washed with blocking buffer and then exposed to the secondary antibody for another 30 to 60 min. After the secondary antibody, membrane was washed with TBST for the last time to clean the background. Protein bands were visualized and quantified using Gene tool system (SynGene-Frederick, MD, USA). The relative content of each protein measured using Western blot analysis was normalized to sample total protein content measured using Ponceau stain and densitometry of total Ponceau in that sample lane. Primary antibody information is as follows: iPLA_2_β (Abcam, no. ab278086), STIM1 (Sigma-Aldrich, no. AB9870), Orai1 (Abcam, no. ab86748), SERCA1 (DSHB, no. CAF2-5D2), SERCA2a (Cell Signaling, no. 4388S), CSQ1 + 2 (Abcam, no. ab3516), 4HNE (Abcam, no. ab46545), PMCA (Thermo Fisher Scientific, no. MA3-914), RyR (DSHB, no. 34C), NKAα1 (Cell Signaling, no. 3010), NKAα2 (Merck Millpore, no. 07-674), calstabin (FKBP12) (Abcam, no. ab2918), CaM (Cell Signaling, no. 4830S), CaMKII (Cell Signaling, no. 4436S), CaN (Cell Signaling, no. 2614S), NFAT (Cell Signaling, no. 5682), calpain 3 (Leica, no. CALP-2C4), PKA substrates (Cell Signaling, no. 9624S), cAMP ELISA kit (Enzo, ADI-900-067A), and MuRF1 (Santa Cruz, sc-398608 HRP).

### Lipid extraction and LC-MS/MS oxylipin analysis

We homogenized GTN muscle and prepared samples for lipid extraction and liquid chromatography–tandem mass spectrometry (LC-MS/MS) lipidomics analysis as we have previously described (*19*). Briefly, we homogenized tissue samples with ceramic beads in 1 ml of antioxidant buffer containing 100 μM diethylenetriaminepentaacetic acid and 100 μM butylated hydroxytoluene in PBS using a Bead Ruptor Elite for 30 s at 6 m/s, under cooled nitrogen gas (4°C). We spiked samples with 12-HETE-d8 (2.5 ng), 15-HETE-d8 (2.5 ng), 13-HODE-d4 (2.3 ng), and standards (Cayman Chemical) prior to homogenization. We then extracted lipids as described by Brown *et al.* (*19*). For oxylipin analysis, we separated lipids by LC using a gradient of 30 to 100% B over 20 min (A: water:Mob B 95:5 + 0.1% glacial acetic acid; B: acetonitrile:methanol 80:15 + 0.1% glacial acetic acid) on an Eclipse Plus C18 Column (Agilent) and analyzed them on a Sciex QTRAP 7500 LC-MS/MS system, with the following source conditions: TEM 475°C, IS −2500, GS1 40, GS2 60, CUR 40, and monitored MRM transitions as previously described (*19*). Details of the analytes were listed in table S1.

### Transcriptome sequencing analysis

Raw sequencing data, gene-level quantification, and differential expression analysis were processed and analyzed by the Discovery Bioinformatics Core at the Oklahoma Nathan Shock Center of Excellence in the Biology of Aging, as we reported previously (*79*). Sequencing was performed on an Illumina NovaSeq 6000 instrument with paired-end 150–base pair reads. Sequence reads were trimmed to remove possible adapter sequences and nucleotides with poor quality using Trimmomatic v.0.36. The trimmed reads were mapped to the *Mus musculus* GRCm39 (mm39) reference genome available on ENSEMBL using the STAR aligner v.2.5.2b. Unique gene hit counts were calculated using featureCounts from the Subread package v.1.5.2. Read-count normalization and differentially expressed analyses were performed using the edgeR package from Bioconductor. Expression values quantile-normalized with the voom function were analyzed for differential expression using the standard functions of the limma package. Moderate *t* test *P* values were adjusted for multiple testing using the FDR method. FDR (*q* value) < 0.05 and absolute log_2_ fold change above 1 were used as criteria to filter significantly differentiated genes. Gene Set Enrichment Analysis (GSEA) was conducted using specialized Bioconductor packages, including fgsea, ReactomePA, and viewPathway, to identify functionally related gene sets (e.g., Gene Ontology terms, KEGG, and Reactome pathways) that were significantly overrepresented among the differentially expressed genes. Each pathway GSEA edge set was used as basis of selection for genes included in the heatmaps showing the pathway activity variation among the four mice phenotypes. IPA (QIAGEN, Redwood City CA; www.qiagenbioinformatics.com/products/ingenuitypathway-analysis) was used for further discovery and interactive exploration of significantly affected statics and causal gene networks, pathways, disease, upstream regulators, and regulatory effects.

### Quantitative PCR array analysis of cAMP and calcium signaling genes

Gene expression profiling of cAMP and calcium signaling pathways was performed using the RT^2^ Profiler PCR Array (QIAGEN, Germantown, MD, USA), following the manufacturer’s instructions. Total RNA was isolated from GTN muscle using the RNeasy Mini Kit (QIAGEN), and RNA concentration and integrity were assessed with a NanoDrop spectrophotometer (Thermo Fisher Scientific). Complementary DNA (cDNA) was synthesized from 2 μg of total RNA using the High-Capacity cDNA Reverse Transcription Kit (Applied Biosystems, no. 2022-10013). The resulting cDNA was combined with SYBR Green qPCR Master Mix (Applied Biosystems, catalog no. A25742) and loaded onto a 384-well RT^2^ Profiler Array plate precoated with primers for 96 genes involved in cAMP and calcium signaling, including internal controls and housekeeping genes (QIAGEN, GeneGlobe ID: PAMM-066Z, no. 330231). Real-time qPCR was performed using a QuantStudio 7 Flex Real-Time PCR System with a 384-well block (Applied Biosystems) under standard cycling conditions: initial denaturation at 95°C for 2 min, followed by 40 cycles of 95°C for 5 s and 60°C for 30 s. Cycle threshold (Ct) values were extracted and analyzed using the QIAGEN GeneGlobe Data Analysis Center. Gene expression was normalized to the geometric mean of housekeeping genes (GAPDH, ACTB, and HPRT1), and differential expression was calculated using the 2^−ΔΔCt^ method. Genes with fold change >2 or <−2 and *P* < 0.05 were considered significantly regulated.

### Statistical analysis

All results are presented as mean values ± standard error of mean (SEM). Comparisons among different groups were performed with robust (nonparametric) two-way analysis of variance (ANOVA) with Tukey’s multiple comparisons adjustment or the Mann-Whitney exact test, with Bonferroni correction for multiple testing. The statistical analysis was undertaken by GraphPad Prism 10, and the statistical significance was set at *P* < 0.05.

## Supplementary Materials

### This PDF file includes:

Figs. S1 to S12 Table S1 Uncropped raw blots
