# Supplementary material for: iPLA2β: A novel store-operated calcium entry modulator contributing to muscle dysfunction during denervation
Source: Sci Adv. 2026 Jul 17;12(29):eaed1646. doi: 10.1126/sciadv.aed1646 (PMC13378579; doi:10.1126/sciadv.aed1646)
Supplement: Supplementary file 1 — Figs. S1 to S12 Table S1 Uncropped raw blots [file sciadv.aed1646_sm.pdf]

Science Advances
AAAS

Supplementary Materials for

iPLA$_2$β: A novel store-operated calcium entry modulator contributing to muscle dysfunction during denervation

Hongyang Xu *et al.*

Corresponding author: Hongyang Xu, yang-xu@omrf.org

Sci. Adv. **12**, eaed1646 (2026)
DOI: 10.1126/sciadv.aed1646

This PDF file includes:

Figs. S1 to S12
Table S1
Uncropped raw blots

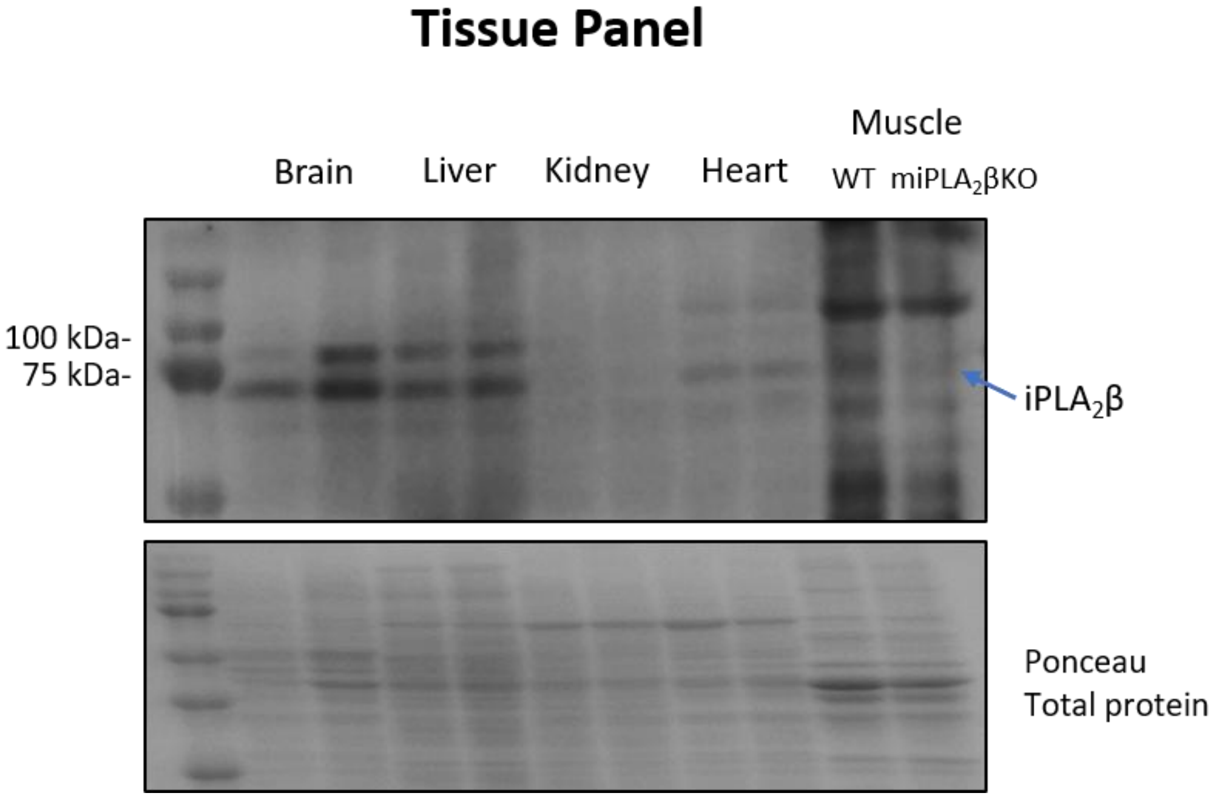

Supplementary Figure 1. **Western blot validation of tissue-specific iPLA$_2$β knockout.** Western blot analysis of iPLA$_2$β expression across multiple tissues from miPLA$_2$βKO mice, including brain, liver, kidney, heart, and gastrocnemius (GTN) muscle. The results confirm that iPLA$_2$β deletion is restricted only to the muscle tissue in miPLA$_2$βKO mice, with normal expression maintained in other organs.

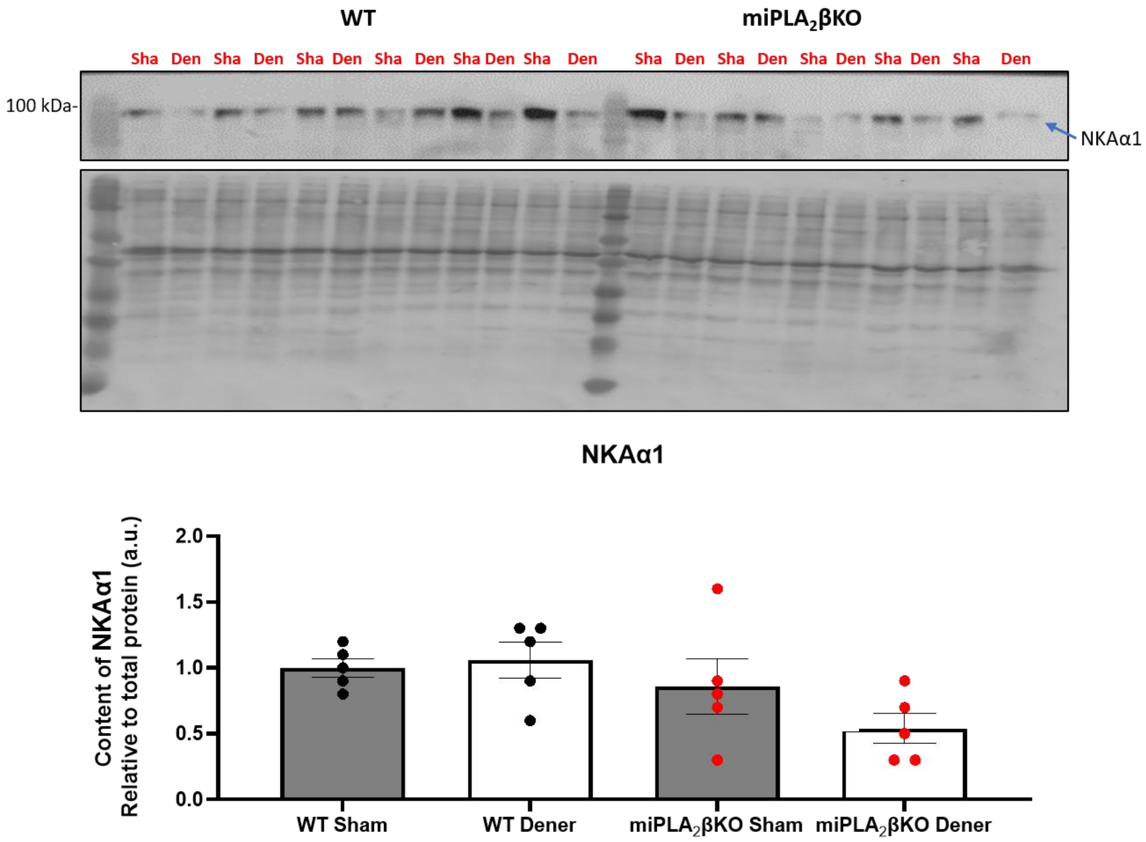

Supplementary Figure 2. Protein content of NKAα1. NKAα1, the ubiquitous isoform of Na^+/K^+-ATPase (NKA), is expressed in all tissue types but at lower levels in skeletal muscle compared to NKAα2. Representative western blot images and pooled quantification show that NKAα1 protein levels remain relatively stable across all experimental groups. *Significant difference between labelled groups ($P < 0.05$, two-way ANOVA). n=4–7 indicates number of animals. Data are presented as mean value ± standard deviation.

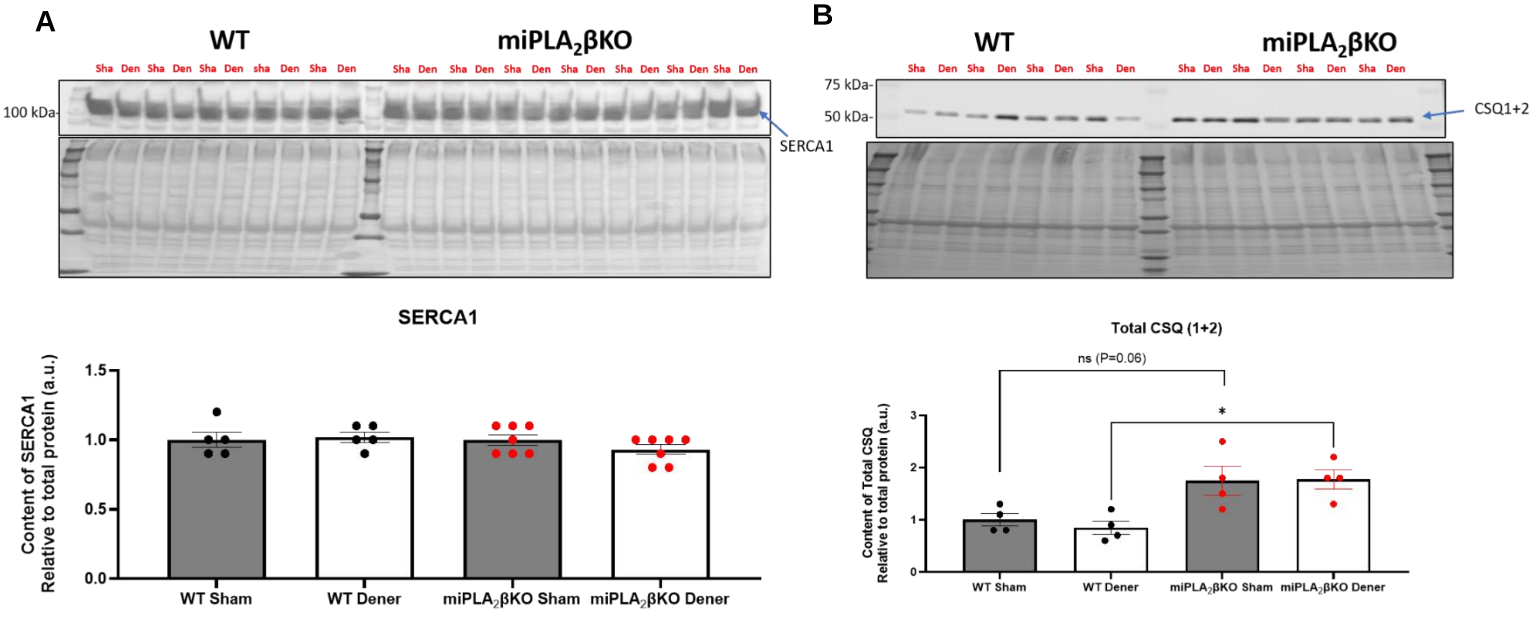

Supplementary Figure 3. Protein content of SERCA1 and calsequestrin (CSQ). SERCA1 is the predominant SERCA isoform in fast-twitch muscle, comprising the majority of SERCA expression in gastrocnemius (GTN) muscle. CSQ (fast isoform CSQ1 and slow isoform CSQ2) is the calcium storing protein in the SR lumen indicative of SR Ca^{2+} storing capacity. **(A)** Representative western blot images and pooled quantification show that SERCA1 protein levels remain quite stable across all experimental groups, confirming again that the impaired SERCA activity is not due to the protein content alterations. (B) Representative western blot images and pooled quantification show that the basic levels of CSQ protein are higher in the miPLA$_2$βKO muscles compared to wildtype muscles. *Significant difference between labelled groups ($P < 0.05$, two-way ANOVA). n=4–7 indicates number of animals. Data are presented as mean value ± standard deviation.

SOCE in denervated muscle with iPLA$_2$β inhibitor BEL

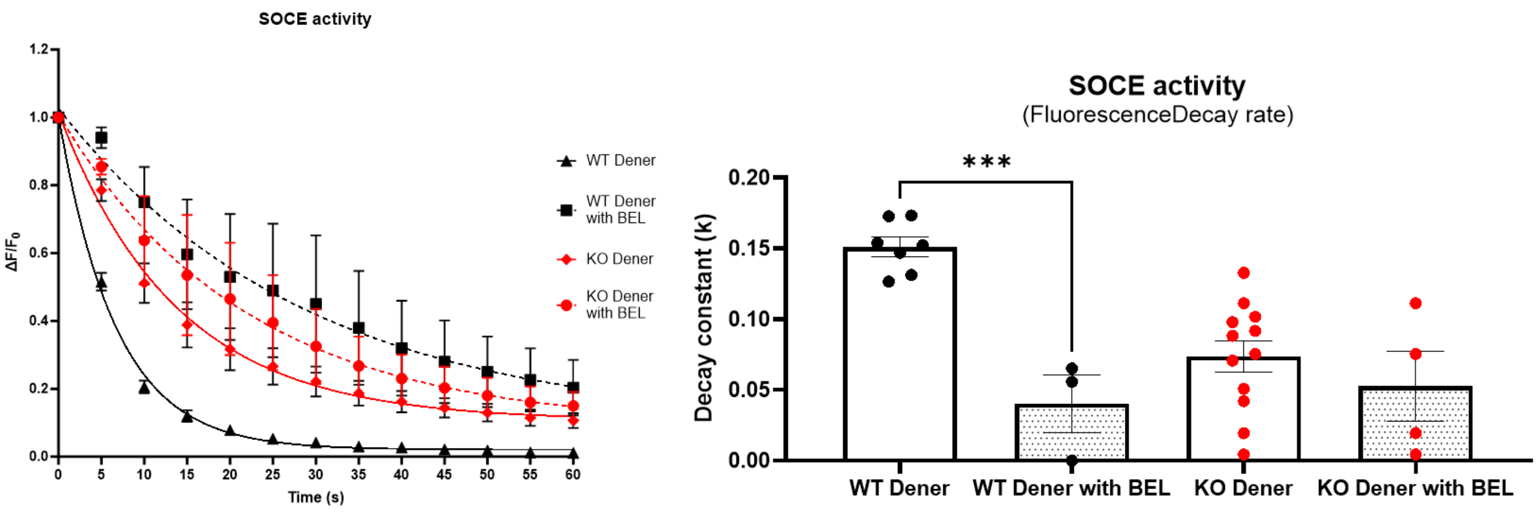

Supplementary Figure 4. SOCE activity in denervated muscles with and without iPLA$_2$β inhibition. To assess the role of iPLA$_2$β in SOCE in denervated muscles, the iPLA$_2$β inhibitor bromoenol lactone (BEL) was applied to denervated muscle fibers from both wildtype and m iPLA$_2$βKO mice. BEL treatment significantly reduced SOCE activity by approximately 75% in wildtype denervated muscles. In contrast, BEL had no significant effect on SOCE activity in m iPLA$_2$βKO denervated muscles, indicating that iPLA$_2$β is required for the enhanced SOCE observed following denervation. *P < 0.05, significant difference between labeled groups (two-way ANOVA). n = 3–12 fibers analyzed, obtained from N = 4 animals per group. Data are presented as mean ± SEM.

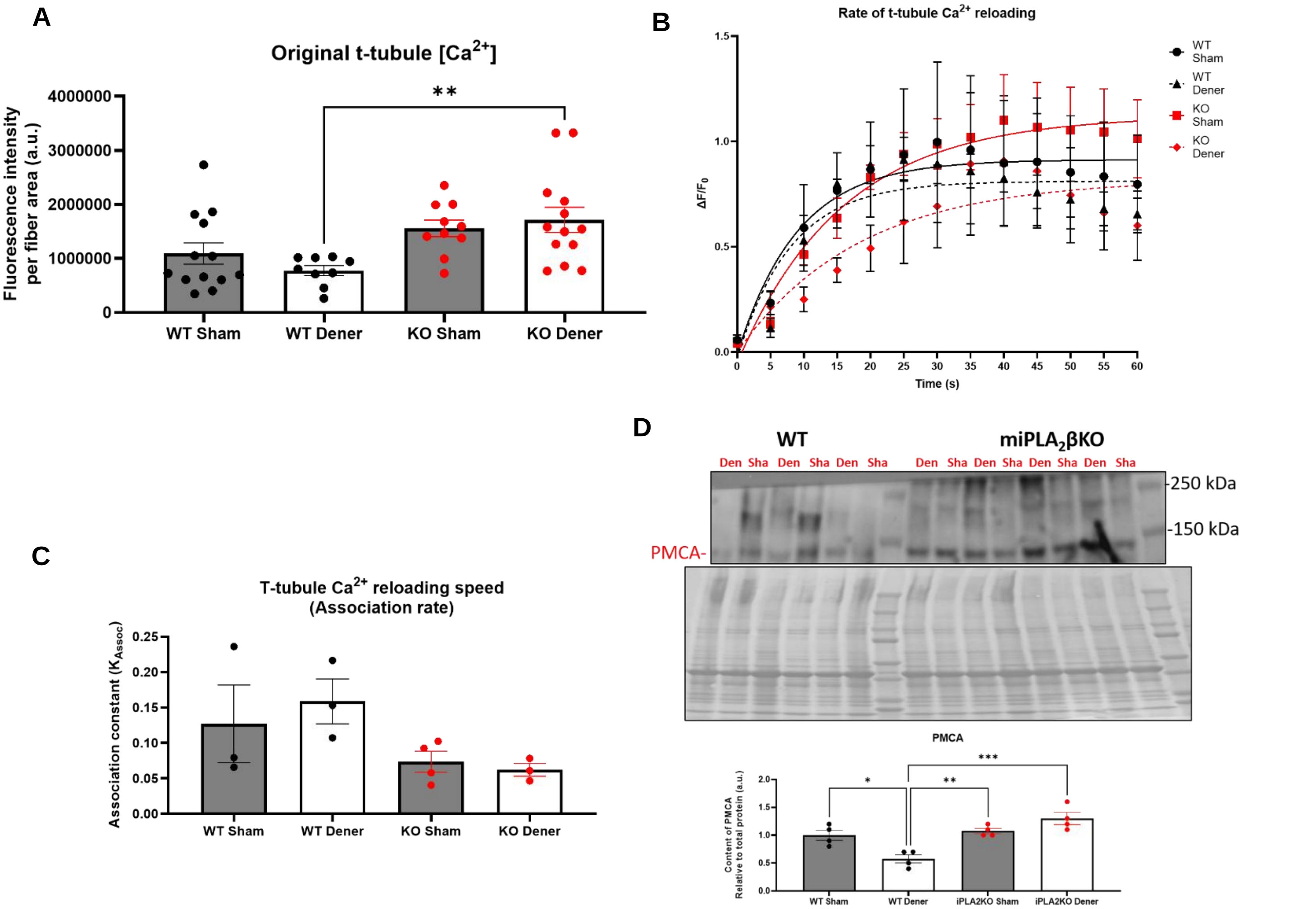

Supplementary Figure 5. Ca^{2+} content in t-tubules and the rate of t-tubule reloading with PMCA content. Before the start of SOCE measurement, the Ca^{2+} fluorescence was imaged in the t-tubules to show the initial Ca^{2+} content in t-tubules before Ca^{2+} loading across different groups. After completion of SOCE measurements, caffeine was washed out and a Ca^{2+} loading buffer containing 200 nM free Ca^{2+} was added to the fiber to allow Ca^{2+} reuptake into the t-tubules via plasma membrane Ca^{2+} ATPase (PMCA) activity to reload t-tubules with Ca^{2+}. **(A)** Fluorescence of the original Ca^{2+} content in t-tubules. **(B)** Best-fit exponential association curve showing recovery of t-tubule Ca^{2+} fluorescence over time (Ca^{2+} reloading). **(C)** Association rate constant (K_{assoc}) was derived and used as an index of PMCA-mediated Ca^{2+} reuptake into the t-tubules for each group. **(D)** Representative immunoblots (top) showing PMCA content (~130 kDa) in wildtype (WT) and miPLA$_2$βKO muscles under sham (Sha) or denervated (Den), with quantified data of PMCA content normalized to total protein (bottom). Denervation significantly decreased PMCA levels in both WT muscles, with a greater increase observed in denervated miPLA$_2$βKO muscles compared with WT denervated muscles. *P < 0.05, significant difference between labeled groups (two-way ANOVA). n = 3–13 fibers analyzed, obtained from N = 4 animals per group. Data are presented as mean ± SEM.

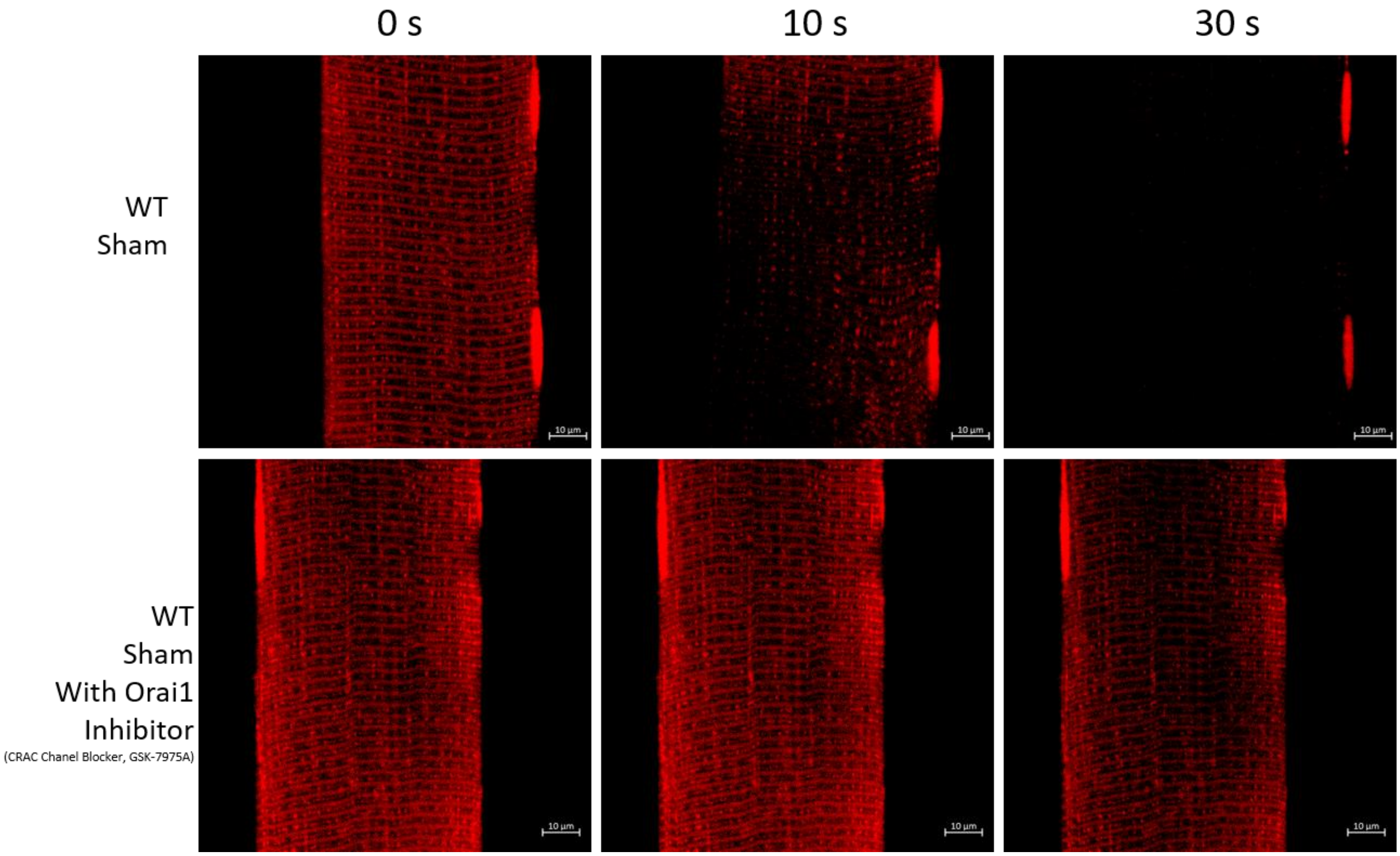

Supplementary Figure 6. Inhibition of SOCE activity by Orai1 inhibitor. Representative confocal images show SOCE activity in wildtype sham muscle fibers, with and without Orai1 inhibitor treatment. The fiber treated with the Orai1 inhibitor exhibits a marked reduction in SOCE activity, indicating effective suppression of Ca^{2+} influx through Orai1 channels.

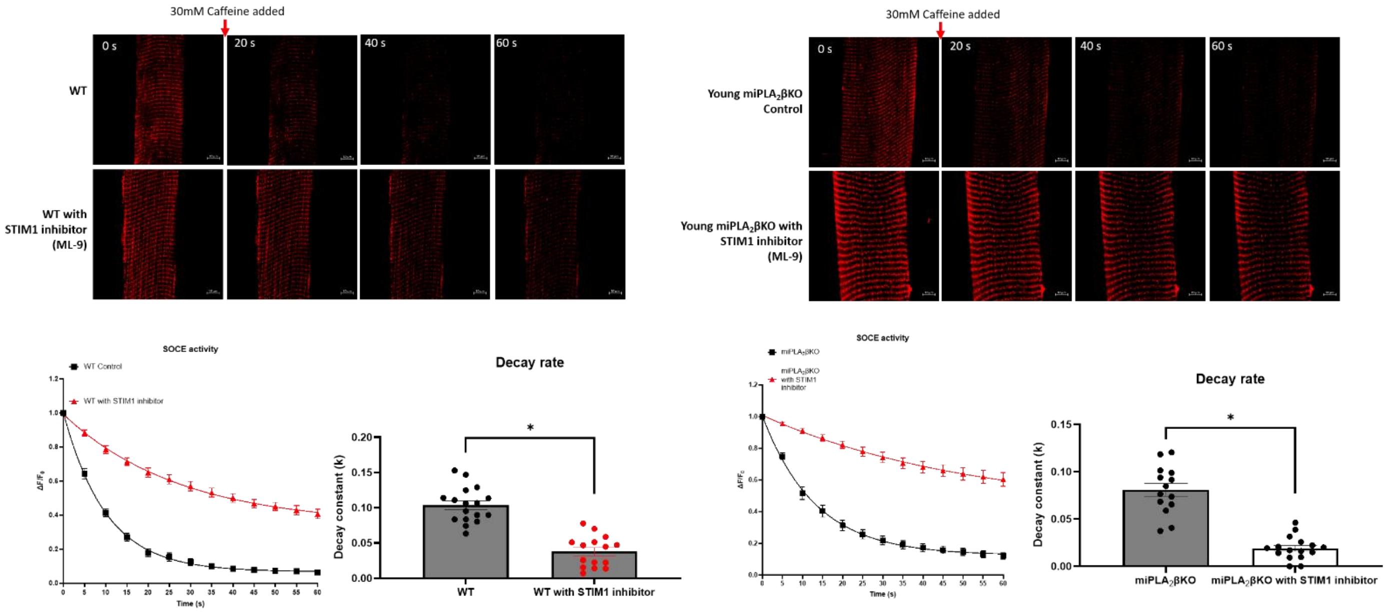

Supplementary Figure 7. Inhibition of SOCE activity by a STIM1 inhibitor. Representative confocal images illustrate SOCE activity in young wild-type and miPLA$_2$βKO muscle fibers in the presence or absence of the STIM1 inhibitor ML-9. In wild-type fibers, ML-9 treatment produced a marked reduction in SOCE activity (~63% decrease), although SOCE was not completely abolished. In miPLA$_2$βKO muscle fibers, ML-9 treatment resulted in a greater reduction in SOCE activity (~77% decrease), indicating that iPLA$_2$β plays a significant role in Orai1 channel activation during SOCE. Representative images with the best-fit fluorescence decay curves and the corresponding decay constants (k) are shown for **(A)** wild-type fibers and **(B)** miPLA$_2$βKO fibers. *$P < 0.05$, significant difference between labeled groups (unpaired t-test). n = 14–16 fibers analyzed, obtained from N = 4–6 animals per group. Data are presented as mean ± SEM.

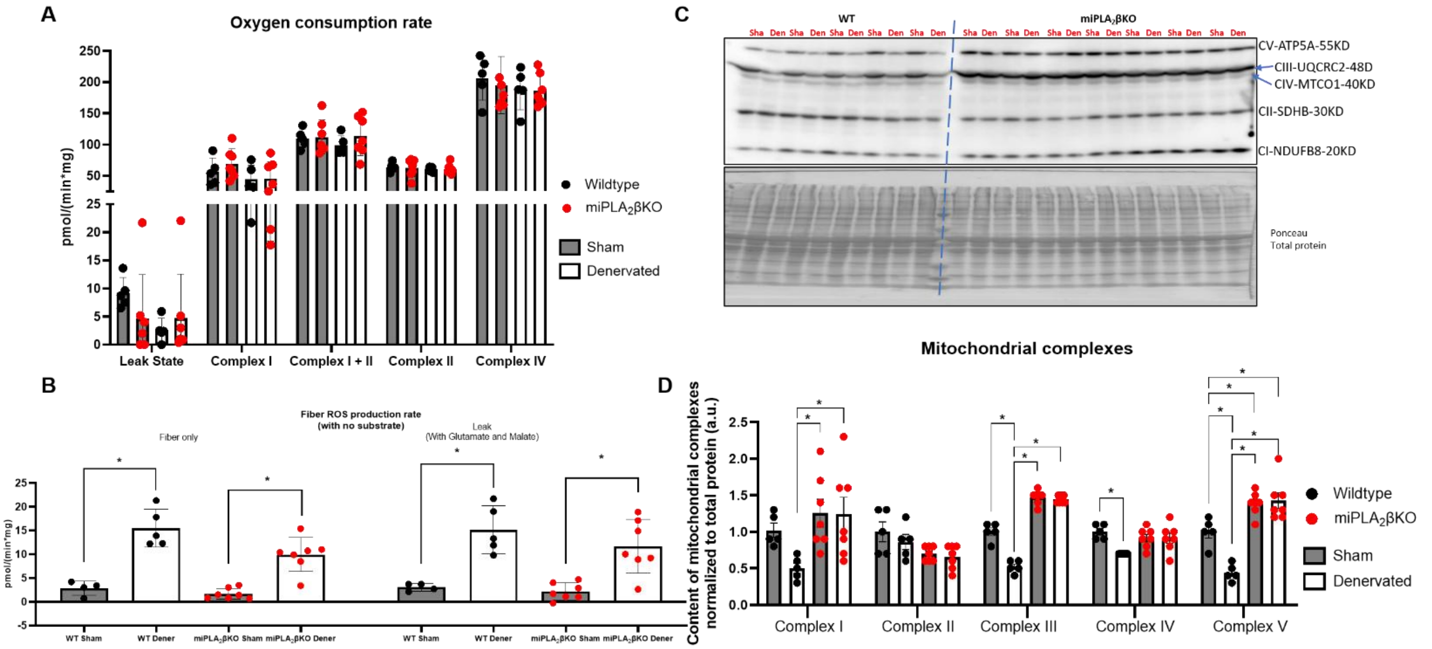

Supplementary Figure 8. Detection of mitochondrial function and content of different complexes. Respiratory functions are measured with Oxygraph-2k technique using permeabilized muscle fibers from GTN muscle. **(A)** Respiratory rate or OCR (for the rate of oxidation phosphorylation) of mitochondria in permeabilized muscle fibers. Substrates used for different complexes added sequentially. Leak state, 10 mM glutamate and 2 mM malate with no adenosine diphosphate (ADP); complex I, addition of 2.5 mM ADP; complex I + II, addition of 10 mM succinate; complex II, addition of 0.5 μM rotenone; complex IV, addition of 2 mM ascorbate and 0.5 mM TMPD added after 5 μM antimycin A. **(B)** ROS production rate of permeabilized muscle fibers with no substrate (Fiber only) or with glutamate and malate (Leak). **(C) & (D)** Representative Western blot images with pooled data for the relative content of mitochondrial complexes, CI-CV, in different groups. *Significant difference between labelled groups ($P < 0.05$, two-way ANOVA). n=4–7 indicates number of animals. Data are presented as mean value ± standard deviation.

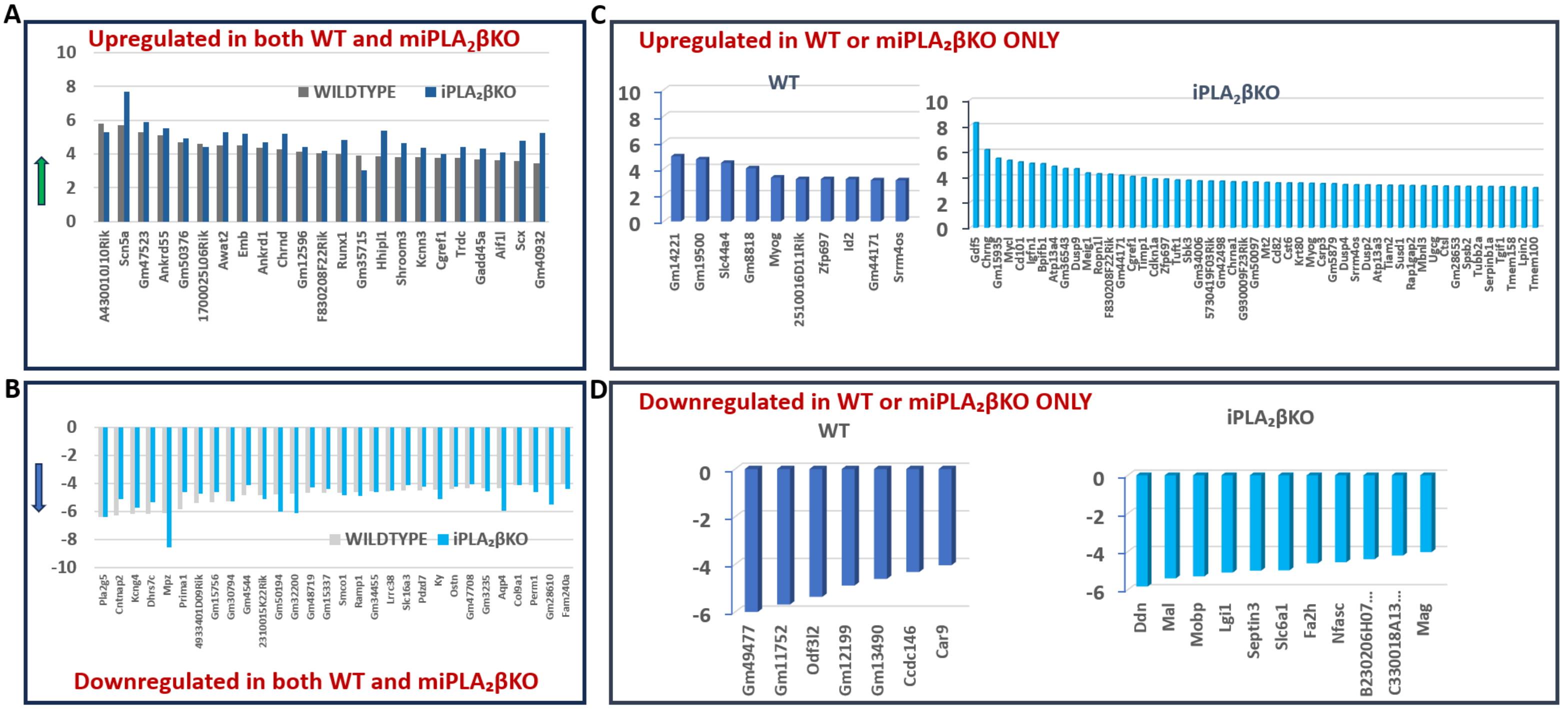

Supplementary Figure 9. Bulk RNA-seq IPA Analysis Reveals Genes Significantly Altered by Denervation in Wildtype vs miPLA$_2$βKO mice. Bar plots showing the log$_2$ fold change of the top differentially expressed genes following denervation. **(A)** Genes that are upregulated 3log$_2$ fold or more in both wildtype and miPLA$_2$βKO muscle after denervation. **(B)** Genes that are downregulated at least 4 log$_2$ fold in both wildtype and miPLA$_2$βKO mice. **(C) & (D)** Genes that are upregulated at least 3 log$_2$ fold or downregulated at least 4 log$_2$ fold in either WT or miPLA$_2$β KO alone. Differential expression analysis was performed using the moderated t-statistic method implemented in the limma package. P-values were adjusted for multiple testing using the False Discovery Rate (FDR) method. Genes were considered significantly differentially expressed if they exhibited a fold change > 2 (either up or down) and FDR < 0.05.

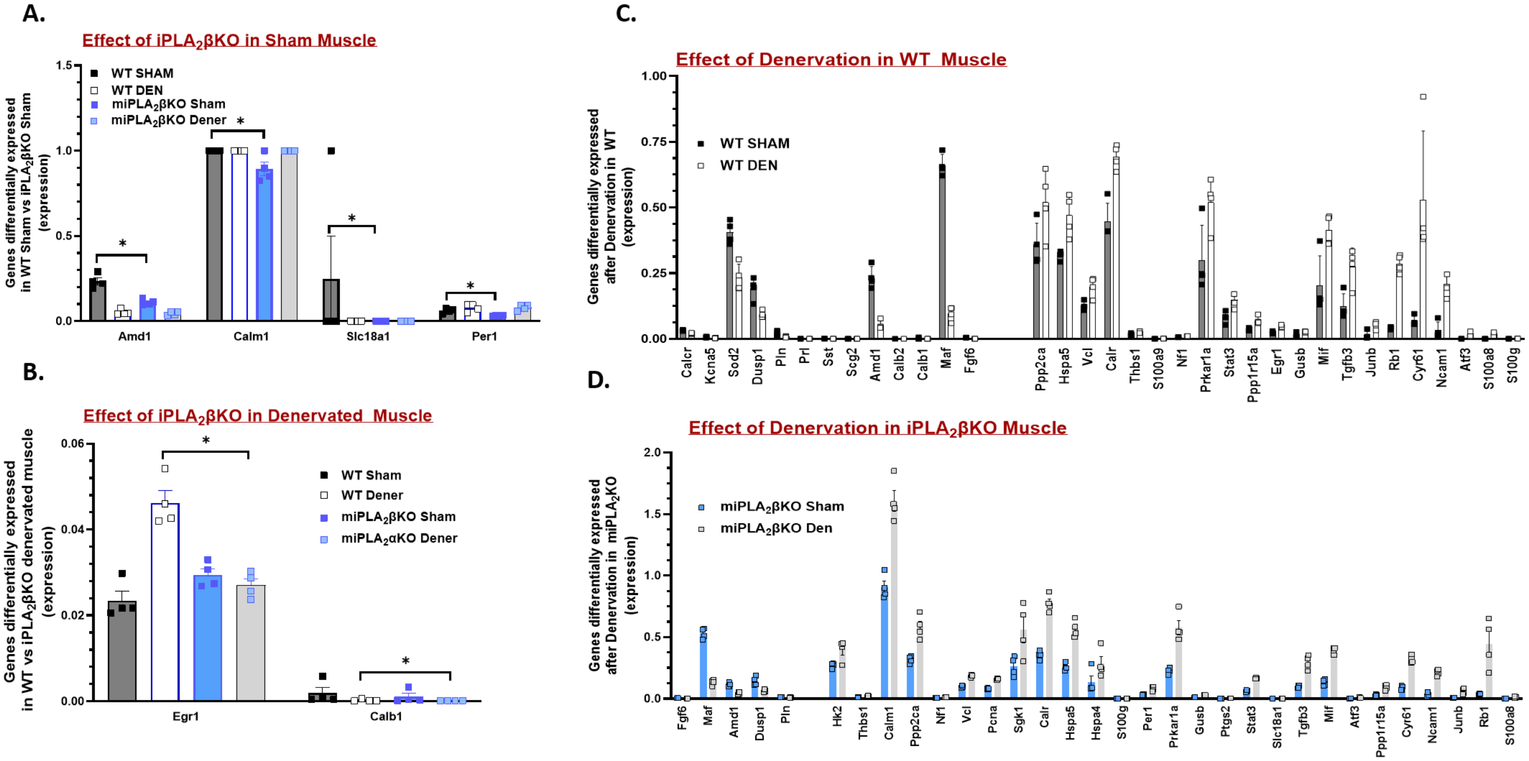

Supplementary Figure 10. Gene expression changes in cAMP and calcium related genes. Transcriptomic alterations in cAMP and calcium signaling pathways were analyzed using targeted signaling array. Each panel represents a different group comparison: **(A)** Comparison of sham muscles between WT and miPLA$_2$βKO mice; **(B)** Changes in denervated muscles between WT and miPLA$_2$βKO mice; **(C)** Changes in WT muscles by denervation; **(D)** Changes in miPLA$_2$βKO muscles by denervation. Bar plots showing PCR-measured ΔΔCt derived expression of the top differentially expressed genes across the four mouse groups. Quantitative real-time PCR was performed using a QuantStudio 7 Flex Real-Time PCR System with a 384-well block (Applied Biosystems) under standard cycling conditions Cycle threshold (Ct) values were extracted and analyzed using the Qiagen GeneGlobe Data Analysis Center. Gene expression was normalized to the geometric mean of housekeeping genes (GAPDH, ACTB, and HPRT1), and differential expression was calculated using the $2^{-ΔΔCt}$ method. Genes with fold change >2 or <−2 and $p < 0.05$ were considered significantly regulated.

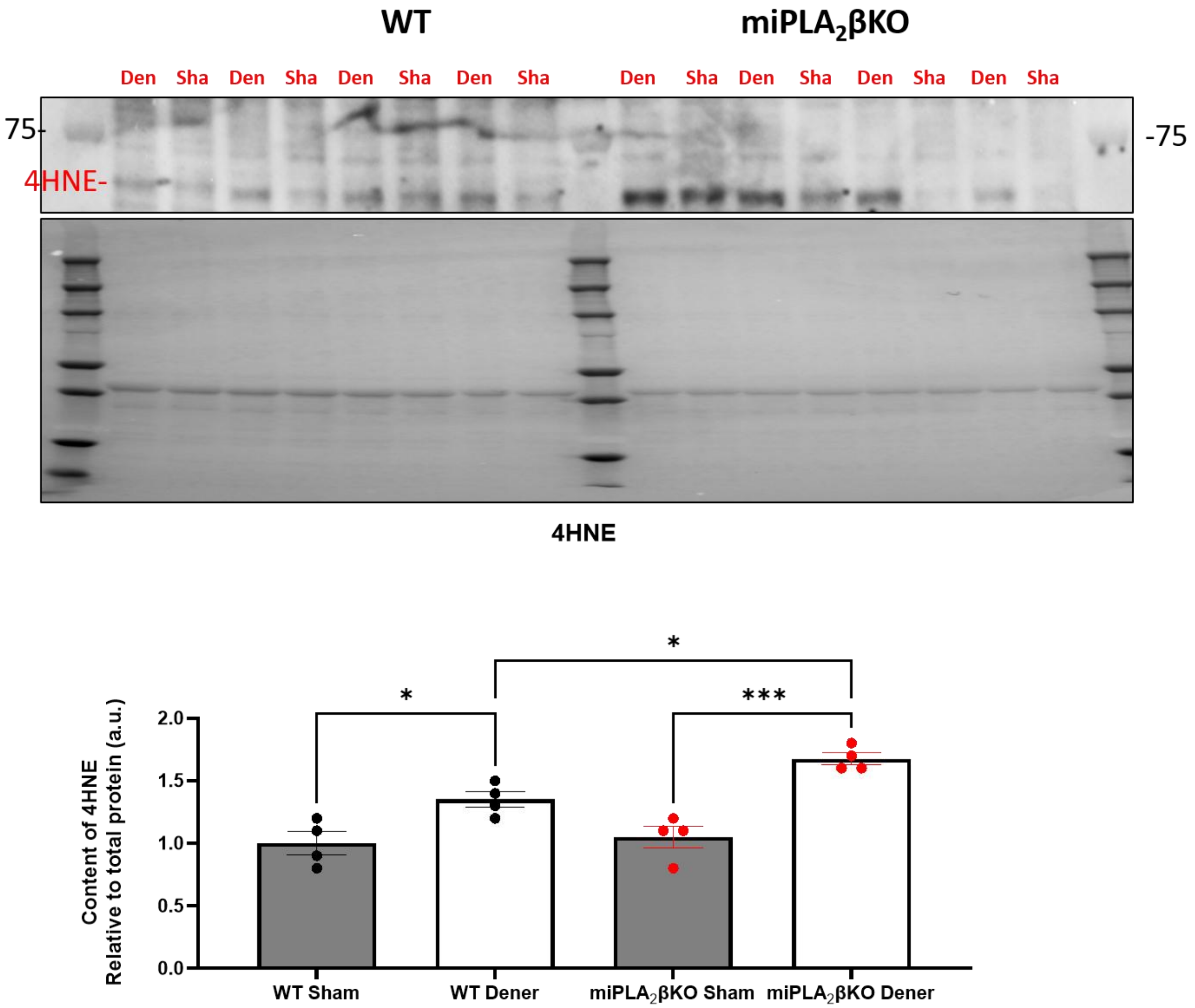

Supplementary Figure 11. Denervation increases protein 4-hydroxynonenal (4HNE) modification in WT and iPLA$_2$βKO muscle. Representative immunoblots showing 4HNE–modified proteins (~73 kDa) in wildtype (WT) and iPLA$_2$βKO muscles under sham (Sha) or denervated (Den) conditions are shown (top), with total protein staining used as a loading control (middle). Quantification of total 4HNE content normalized to total protein is shown below. Denervation significantly increased 4HNE levels in both WT and miPLA$_2$βKO muscles, with a greater increase observed in denervated miPLA$_2$βKO muscles compared with WT denervated muscles. Data are presented as mean ± SEM. n=4 indicate number of animals, *Significant difference between labelled groups ($P < 0.05$, two-way ANOVA).

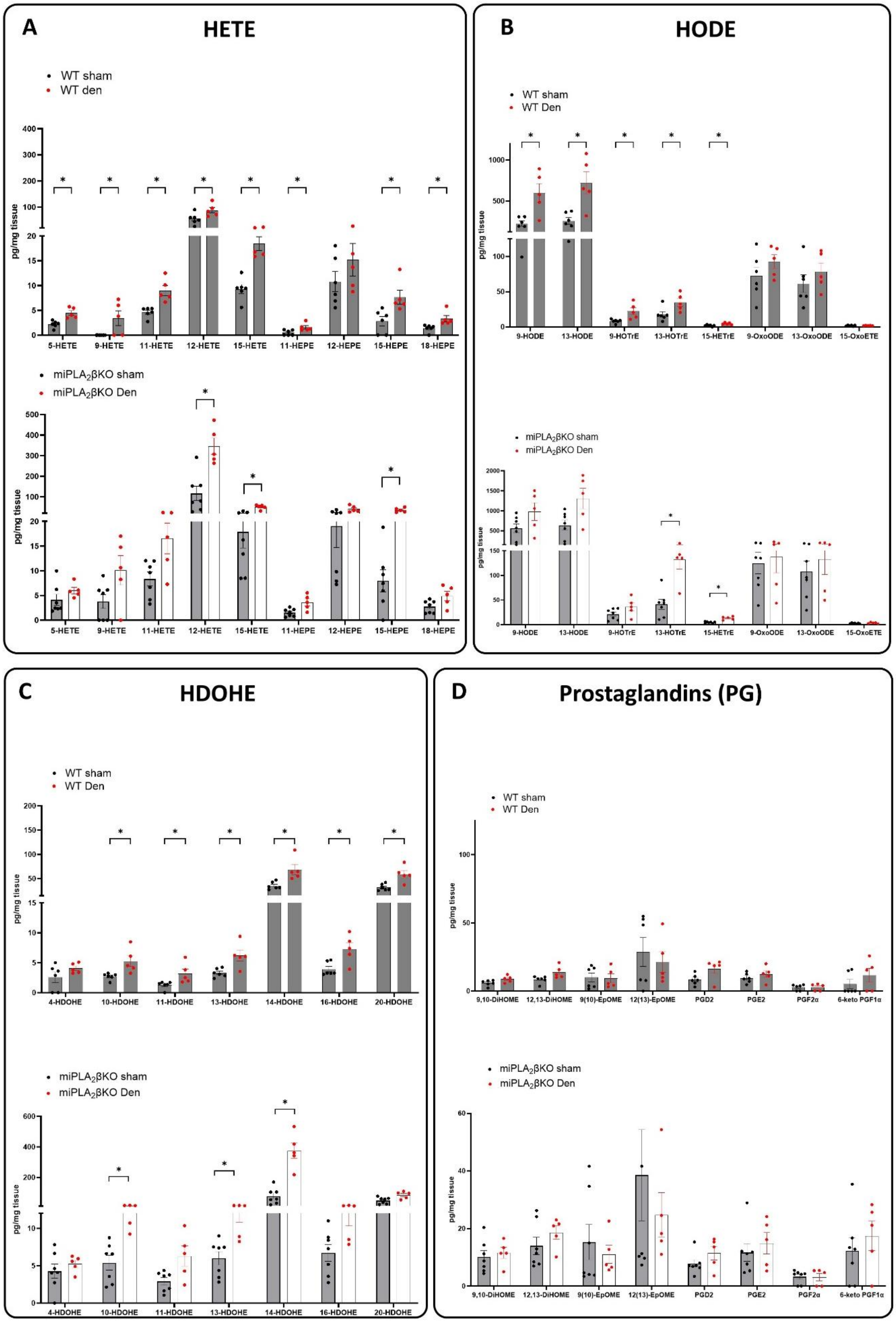

Supplementary Figure 12. Lipidomic analysis of oxylipin contents with comparison between sham and denervated muscles in wildtype and miPLA2βKO mice respectively. Quantification of oxylipin species in sham (black point) and denervated (red point) gastrocnemius muscle from wildtype (top bar graph of each panel) and miPLA2βKO (bottom bar graph of each panel) mice. Oxylipin classes analyzed include (A) HETEs, (B) HODEs, (C) HDOHEs, (D) PGs. *Significant difference between labelled groups ($P < 0.05$, unpaired t-test). n=5–7 mice. Data are presented as mean value ± SEM.

Oxylipins

Analyte	Internal Standard (IS)	Retention Time (RT; min)	Declustering potential (DP)	Collision energy (CE)	Molecular mass (g/mol)	Transition		Calibration range (nM)		R^{2} **
						Q1 (m/z)	Q3 (m/z)	lower[1]	upper	
5-HETE	5(S)-HETE-d8	14.4	-55	-19	320.5	319.20	115.2	1	2000	0.9974
8-HETE	5(S)-HETE-d8	14.1	-65	-18	320.5	319.20	155.201	3	1000	0.9988
9-HETE	5(S)-HETE-d8	14.27	-50	-20	320.5	319.20	167.2	1	1000	0.9975
11-HETE	12(S)-HETE-d8	13.91	-60	-19	320.5	319.20	167.202	0.3	1000	0.9989
12-HETE	12(S)-HETE-d8	14.11	-65	-18	320.5	319.20	179.2	1	2000	0.9977
15-HETE	15(S)-HETE-d8	13.65	-55	-18	320.5	319.20	219.2	1	2000	0.9981
20-HETE	20-HETE-d6	12.64	-85	-21	320.5	319.20	275.1	10	1000	0.9971
5-HEPE	5(S)-HETE-d8	13.17	-60	-20	318.5	317.20	115.1	1	1000	0.9987
8-HEPE	5(S)-HETE-d8	12.8	-65	-19	318.5	317.20	155.2	1	1000	0.9992
9-HEPE	5(S)-HETE-d8	12.99	-50	-18	318.5	317.50	167.2	1	1000	0.9991
11-HEPE	12(S)-HETE-d8	12.69	-50	-20	318.5	317.20	167.201	0.3	1000	0.9981
12-HEPE	12(S)-HETE-d8	12.91	-65	-18	318.5	317.20	179.2	1	1000	0.9985
15-HEPE	15(S)-HETE-d8	12.63	-65	-16	318.5	317.20	219.2	1	1000	0.9992
18-HEPE	15(S)-HETE-d8	12.25	-50	-15	318.5	317.20	259.2	1	1000	0.9996
4-HDOHE	5(S)-HETE-d8	14.66	-50	-17	344.5	343.20	101.1	1	2000	0.9984
7-HDOHE	5(S)-HETE-d8	14.2	-50	-21	344.5	343.20	141.2	10	1000	0.9975
8-HDOHE	5(S)-HETE-d8	14.31	-50	-19	344.5	343.20	189.2	3	1000	0.9985
10-HDOHE	12(S)-HETE-d8	13.99	-55	-21	344.5	343.20	153.201	1	1000	0.9994
11-HDOHE	12(S)-HETE-d8	14.14	-60	-18	344.5	343.20	121.1	1	1000	0.9988
13-HDOHE	12(S)-HETE-d8	13.87	-55	-19	344.5	343.20	193.1	0.3	1000	0.9995
14-HDOHE	15(S)-HETE-d8	13.99	-45	-17	344.5	343.20	205.2	1	1000	0.9996
16-HDOHE	15(S)-HETE-d8	13.73	-55	-17	344.5	343.20	233.201	0.3	1000	0.9996
17-HDOHE	20-HETE-d6	13.79	-70	-15	344.5	343.20	201.2	30	1000	0.9972
20-HDOHE	20-HETE-d6	13.47	-55	-17	344.5	343.20	241.201	1	1000	0.9982
9-HODE	13(S)-HODE-d4	13.34	-85	-23	296.5	295.20	171.1	3	2000	0.9957
13-HODE	13(S)-HODE-d4	13.28	-85	-23	296.5	295.20	195.2	3	2000	0.9970
9-HOTrE	13(S)-HODE-d4	12	-60	-20	294.4	293.20	171.2	1	2000	0.9990
13-HOTrE	13(S)-HODE-d4	12.2	-70	-22	294.4	293.20	195.101	3	2000	0.9987
5-HETrE	13(S)-HODE-d4	15.49	-70	-19	322.5	321.20	115.1	0.3	2000	0.9990
15-HETrE	13(S)-HODE-d4	14.29	-70	-21	322.5	321.20	221.2	1	2000	0.9978
9-OxoODE	13(S)-HODE-d4	14	-85	-23	294.4	293.20	185.1	3	1000	0.9958
13-OxoODE	13(S)-HODE-d4	13.72	-85	-25	294.4	293.20	195.1	10	1000	0.9922
5-OxoETE	5(S)-HETE-d8	15.06	-65	-20	318.5	317.20	273.2	3	1000	0.9947
12-OxoETE	5(S)-HETE-d8	14.36	-75	-20	318.5	317.20	153.1	3	1000	0.9957
15-OxoETE	5(S)-HETE-d8	14	-60	-22	318.5	317.20	113.1	1	1000	0.9986
9,10-DiHOME	Leukotriene B4-d4	10.9	-80	-29	314.5	313.20	201.2	0.3	1000	0.9971
12,13-DiHOME	Leukotriene B4-d4	10.62	-80	-28	314.5	313.20	183.2	0.3	1000	0.9976
5,6-DiHETrE	15(S)-HETE-d8	12.64	-75	-24	338.5	337.20	145.1	0.3	1000	0.9989
8,9-DiHETrE	15(S)-HETE-d8	12.14	-70	-25	338.5	337.20	127.1	1	1000	0.9989
11,12-DiHETrE	15(S)-HETE-d8	11.79	-65	-26	338.5	337.20	167.1	0.3	1000	0.9994
14,15-DiHETrE	15(S)-HETE-d8	11.45	-65	-25	338.5	337.20	207.1	0.1	1000	0.9994
5,6-DiHETE	Leukotriene B4-d4	11.2	-60	-23	336.5	335.20	115.2	100	1000	0.9936
5,15-DiHETE	Leukotriene B4-d4	9.92	-60	-21	336.5	335.30	115.2	1	1000	0.9980
8,15-DiHETE	Leukotriene B4-d4	9.63	-65	-22	336.5	335.20	235.2	3	1000	0.9964
14,15-DiHETE	Leukotriene B4-d4	10.35	-65	-23	336.5	335.30	207.2	3	1000	0.9971
17,18-DiHETE	Leukotriene B4-d4	9.97	-65	-24	336.5	335.30	247.2	1	1000	0.9978
RvE1	Resolvin D1-d5	3.21	-65	-22	350.5	349.30	195.1	3	1000	0.9978
RvD1	Resolvin D1-d5	7.47	-55	-23	376.5	375.50	215.1	1	1000	0.9975
RvD2	Resolvin D1-d5	6.8	-65	-21	376.5	375.20	141.2	10	1000	0.9961
RvD3	Resolvin D1-d5	6.49	-65	-24	376.5	375.20	147.1	0.3	1000	0.9990
RvD5	Resolvin D1-d5	10.09	-65	-22	360.5	359.20	199.1	1	1000	0.9984
LTB3	Leukotriene B4-d4	11.5	-65	-22	338.5	337.20	195.2	3	1000	0.9943
LTB4	Leukotriene B4-d4	10.22	-70	-23	336.5	335.20	195.1	1	1000	0.9981
20-carboxy LTB4	Leukotriene B4-d4	3.24	-80	-25	366.5	365.20	347.2	10	1000	0.9964
20-hydroxy LTB4	Leukotriene B4-d4	3.55	-80	-25	352.5	351.20	195.2	1	1000	0.9975
6-trans LTB4	Leukotriene B4-d4	9.89	-65	-23	336.5	335.20	195.101	1	1000	0.9969
LXA4	Resolvin D1-d5	7.32	-55	-19	352.5	351.20	115.2	1	1000	0.9997
Mar-01	Leukotriene B4-d4	10.1	-60	-23	360.5	359.50	250.2	1	1000	0.9953
7,17-DiHDPA	Leukotriene B4-d4	10.38	-65	-20	362.5	361.50	263.3	3	1000	0.9967
9(10)-EpOME	11(12)-EET (EpETrE) -d11	14.86	-80	-21	296.5	295.30	171.1	0.3	1000	0.9976

12(13)-EpOME	11(12)-EET (EpETrE) -d11	14.74	-80	-19	296.5	295.30	195.2	3	1000	0.9959
5(6)-EET	11(12)-EET (EpETrE) -d11	15.37	-60	-16	320.5	319.20	191.1	3	1000	0.9893
8(9)-EET	11(12)-EET (EpETrE) -d11	15.15	-60	-15	320.5	319.30	167.201	1	1000	0.9968
11(12)-EET	11(12)-EET (EpETrE) -d11	15.15	-60	-18	320.5	319.30	167.2	1	1000	0.9970
14(15)-EET	11(12)-EET (EpETrE) -d11	14.84	-65	-18	320.5	319.20	219.3	3	1000	0.9977
8(9)-EpETE	11(12)-EET (EpETrE) -d11	14.2	-70	-18	318.5	317.20	127.2	10	1000	0.9966
11(12)-EpETE	11(12)-EET (EpETrE) -d11	14.12	-70	-15	318.5	317.20	167.2	1	1000	0.9954
14(15)-EpETE	11(12)-EET (EpETrE) -d11	14.04	-70	-18	318.5	317.20	207.2	1	1000	0.9904
17(18)-EpETE	11(12)-EET (EpETrE) -d11	13.7	-75	-16	318.5	317.20	215.2	1	1000	0.9977
7(8)-EpDPA	11(12)-EET (EpETrE) -d11	15.2	-60	-16	344.5	343.20	113.1	3	1000	0.9957
10(11)-EpDPA	11(12)-EET (EpETrE) -d11	15.08	-65	-15	344.5	343.20	153.2	0.3	1000	0.9970
13(14)-EpDPA	11(12)-EET (EpETrE) -d11	15.02	-70	-15	344.5	343.20	193.2	1	1000	0.9966
16(17)-EpDPA	11(12)-EET (EpETrE) -d11	14.97	-55	-16	344.5	343.20	233.2	1	1000	0.9984
19(20)-EpDPA	11(12)-EET (EpETrE) -d11	14.71	-70	-18	344.5	343.20	241.2	3	1000	0.9969
PGD1	Prostaglandin D2-d4	6.65	-55	-16	354.5	353.30	317.202	0.3	1000	0.9912
PGD2	Prostaglandin D2-d4	6.61	-50	-22	352.5	351.20	271.302	0.3	1000	0.9936
PGD3	Prostaglandin D2-d4	5.26	-50	-17	350.5	349.30	269.201	1	1000	0.9939
PGE1	Prostaglandin E2-d4	6.53	-60	-18	354.5	353.30	317.2	0.3	1000	0.9980
PGE2	Prostaglandin E2-d4	6.2	-60	-19	352.5	351.20	271.3	0.3	1000	0.9981
PGE3	Prostaglandin E2-d4	4.86	-60	-17	350.5	349.30	269.2	1	1000	0.9992
PGB2	Prostaglandin D2-d4	8.82	-60	-24	334.5	333.30	175.1	0.3	600	0.9953
13,14-dihydro-15-keto PGE2	Prostaglandin E2-d4	7.33	-55	-19	352.5	351.20	235.2	3	1000	0.9979
13,14-dihydro-15-keto PGD2	Prostaglandin D2-d4	8.16	-50	-25	352.5	351.50	207.2	0.3	1000	0.9943
13,14-dihydro-15-keto PF2α	Prostaglandin F2α-d4	7.43	-55	-23	352.5	353.50	113.001	10	1000	0.9955
11β-PGE2	Prostaglandin E2-d4	6.38	-55	-23	352.5	351.20	271.2	0.3	1000	0.9984
6-keto PGE1	Prostaglandin E2-d4	3.22	-55	-23	368.5	367.20	143.1	1	1000	0.9995
8-iso PGE2	Prostaglandin E2-d4	5.94	-55	-21	352.5	351.20	271.001	1	1000	0.9995
15-deoxy-Δ12,14-PGJ2	20-HETE-d6	12.44	-65	-18	316.4	315.20	271.2	1	1000	0.9972
8-iso-15-keto PGF2α	Prostaglandin F2α-d4	5.37	-50	-23	354.5	351.20	289.2	1	1000	0.9983
PGF2α	Prostaglandin F2α-d4	5.89	-85	-24	354.5	353.20	309.2	1	1000	0.9940
6-keto PGF1α	Prostaglandin F2α-d4	3.3	-75	-26	370.5	369.30	163.2	10	1000	0.9984
TXB2	Thromboxane B2-d4	4.83	-60	-22	370.5	369.20	169.1	1	1000	0.9990
11-dehydro TXB2	11-dehydro Thromboxane B2-d4	6.24	-60	-20	368.5	367.20	305.2	1	1000	0.9992
13(S)-HODE-d4	N/A	13.22	-60	-25	300.2	299.2	198.1	N/A	N/A	N/A
5(S)-HETE-d8	N/A	14.32	-55	-19	328.2	327.2	116.1	N/A	N/A	N/A
12(S)-HETE-d8	N/A	14.02	-60	-20	328.2	327.2	184.1	N/A	N/A	N/A
15(S)-HETE-d8	N/A	13.55	-65	-22	328.2	327.2	226.1	N/A	N/A	N/A
20-HETE-d6	N/A	12.6	-70	-21	326.2	325.2	281.1	N/A	N/A	N/A
Leukotriene B4-d4	N/A	10.17	-65	-21	340.2	339.2	197.1	N/A	N/A	N/A
Resolvin D1-d5	N/A	7.41	-75	-18	381.2	380.2	141.1	N/A	N/A	N/A
Prostaglandin E2-d4	N/A	6.16	-60	-23	356.2	355.2	275.101	N/A	N/A	N/A
Prostaglandin D2-d4	N/A	6.58	-55	-23	356.2	355.2	275.1	N/A	N/A	N/A
Prostaglandin F2α-d4	N/A	5.86	-80	-24	358.2	357.2	313.2	N/A	N/A	N/A
Thromboxane B2-d4	N/A	4.79	-55	-22	374.2	373.2	173.1	N/A	N/A	N/A
11-dehydro Thromboxane B2-d4	N/A	6.21	-55	-21	372.2	371.2	309.2	N/A	N/A	N/A
11(12)-EET (EpETrE) -d11	N/A	15.09	-65	-18	331.2	330.2	167.1	N/A	N/A	N/A

Supplementary Table 1. Details of the oxylipin assay analytes. * for 100 nM, ** weighting 1/x2

Uncropped blots:

Fig. 1B

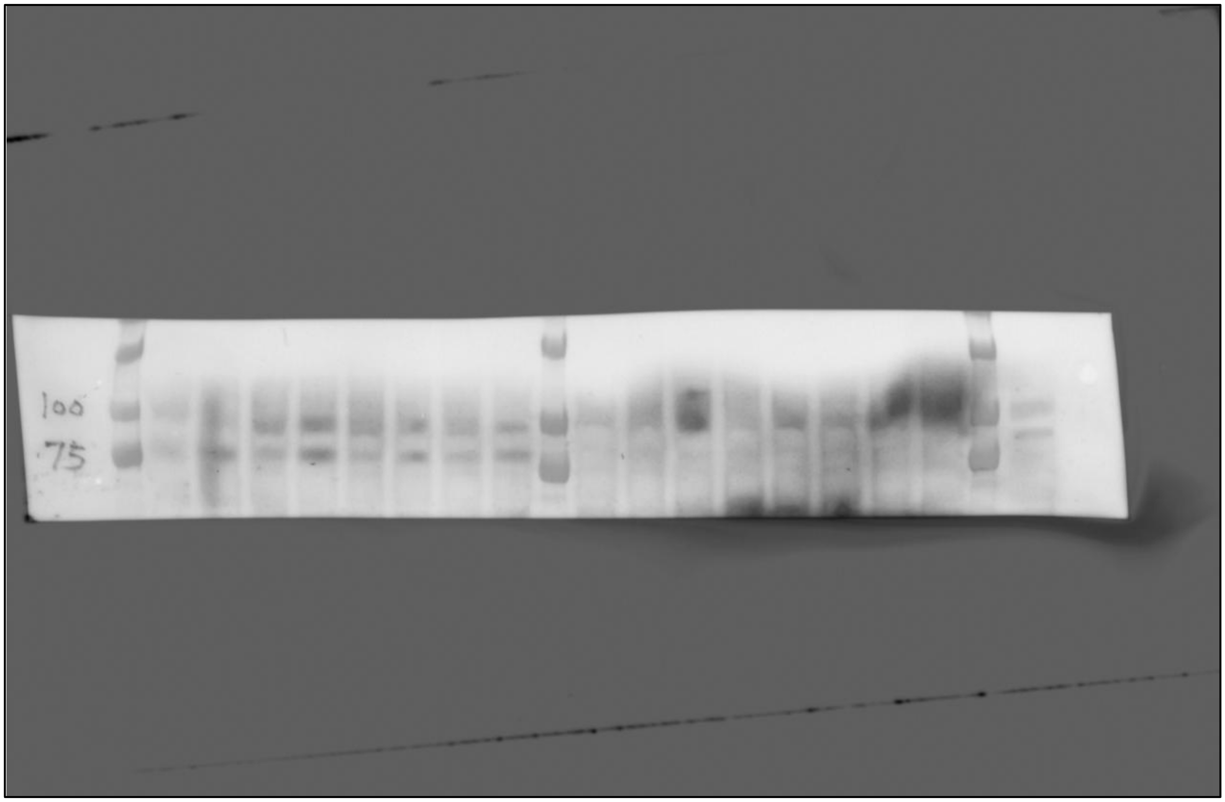

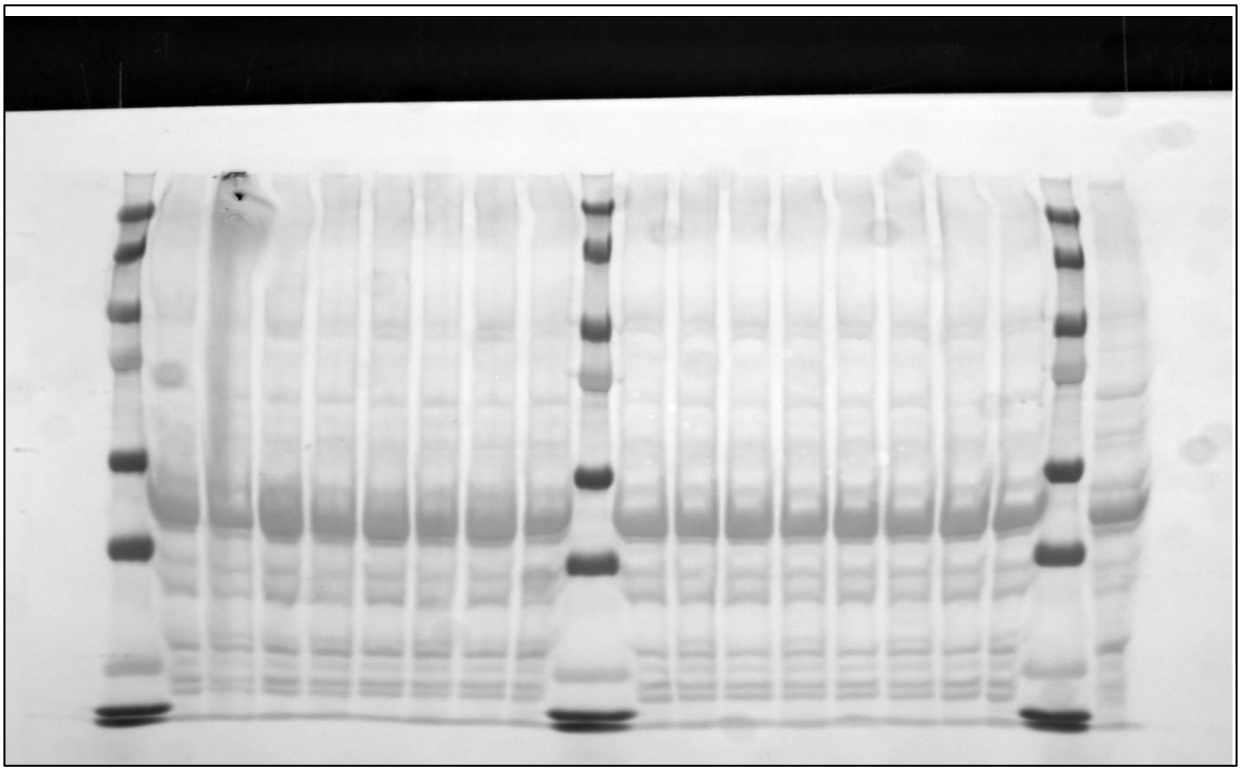

Fig. 3D

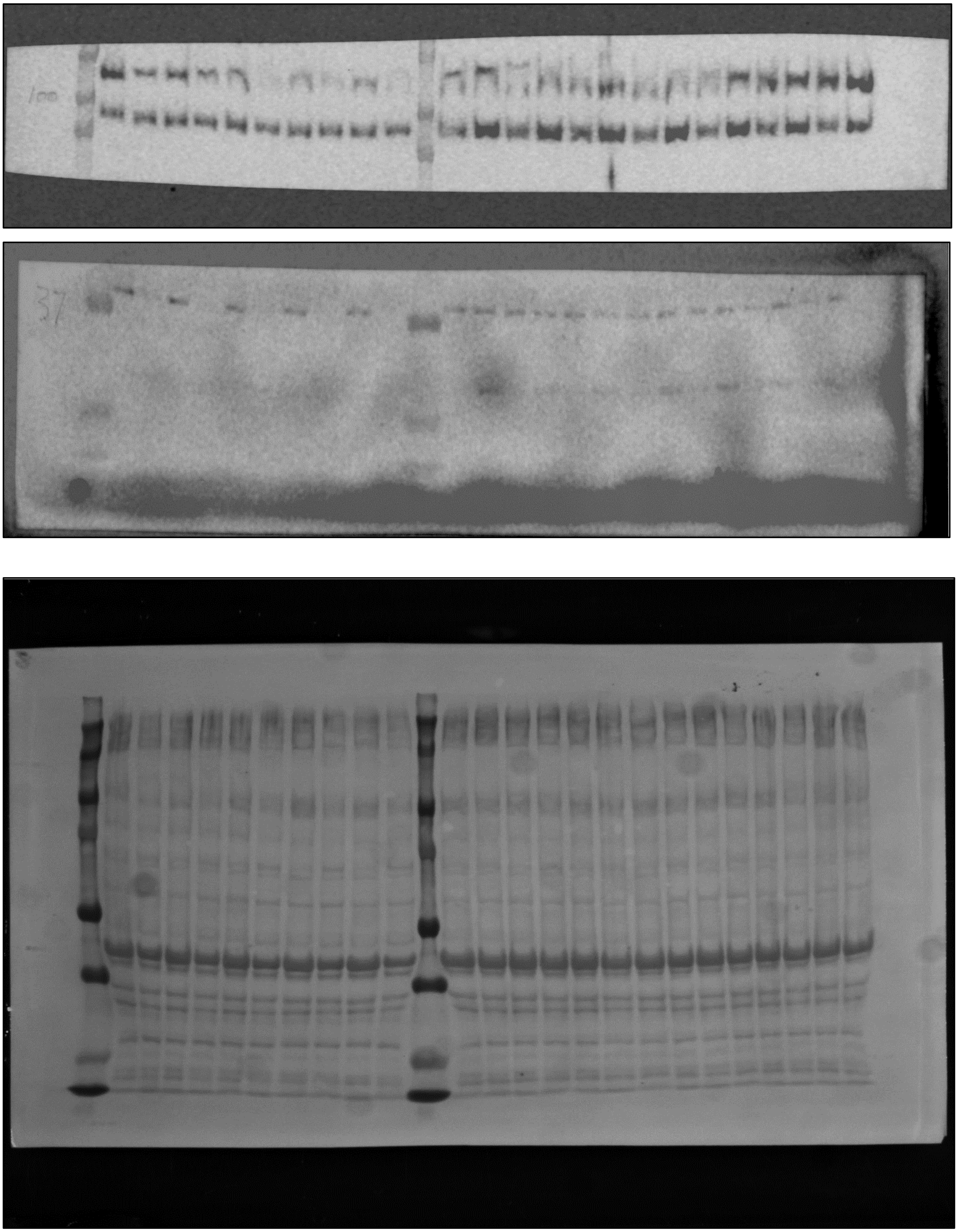

Fig. 3H

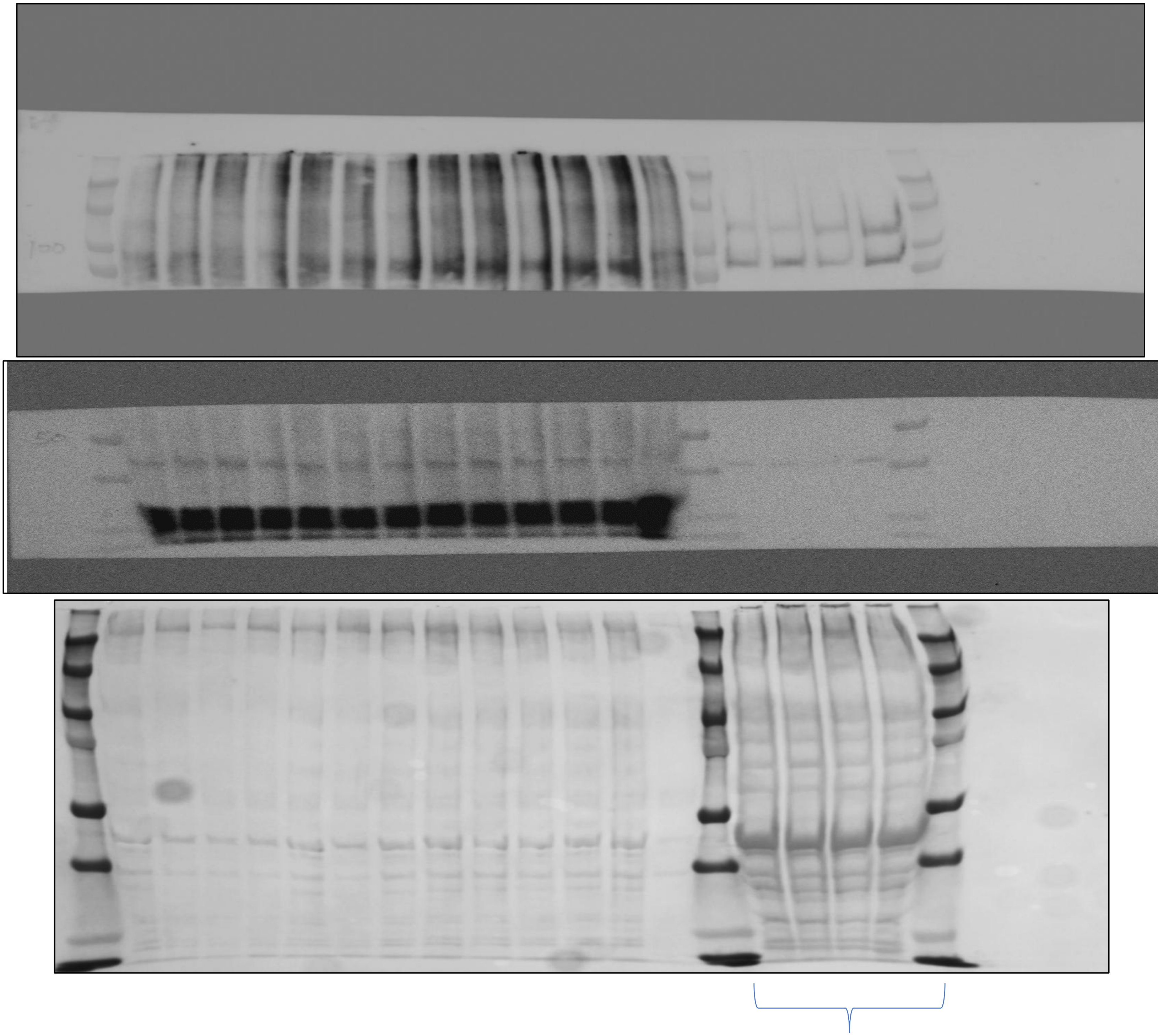

This part of blot was generated for a separate experiment and was not included in the present study.

Fig. 4D

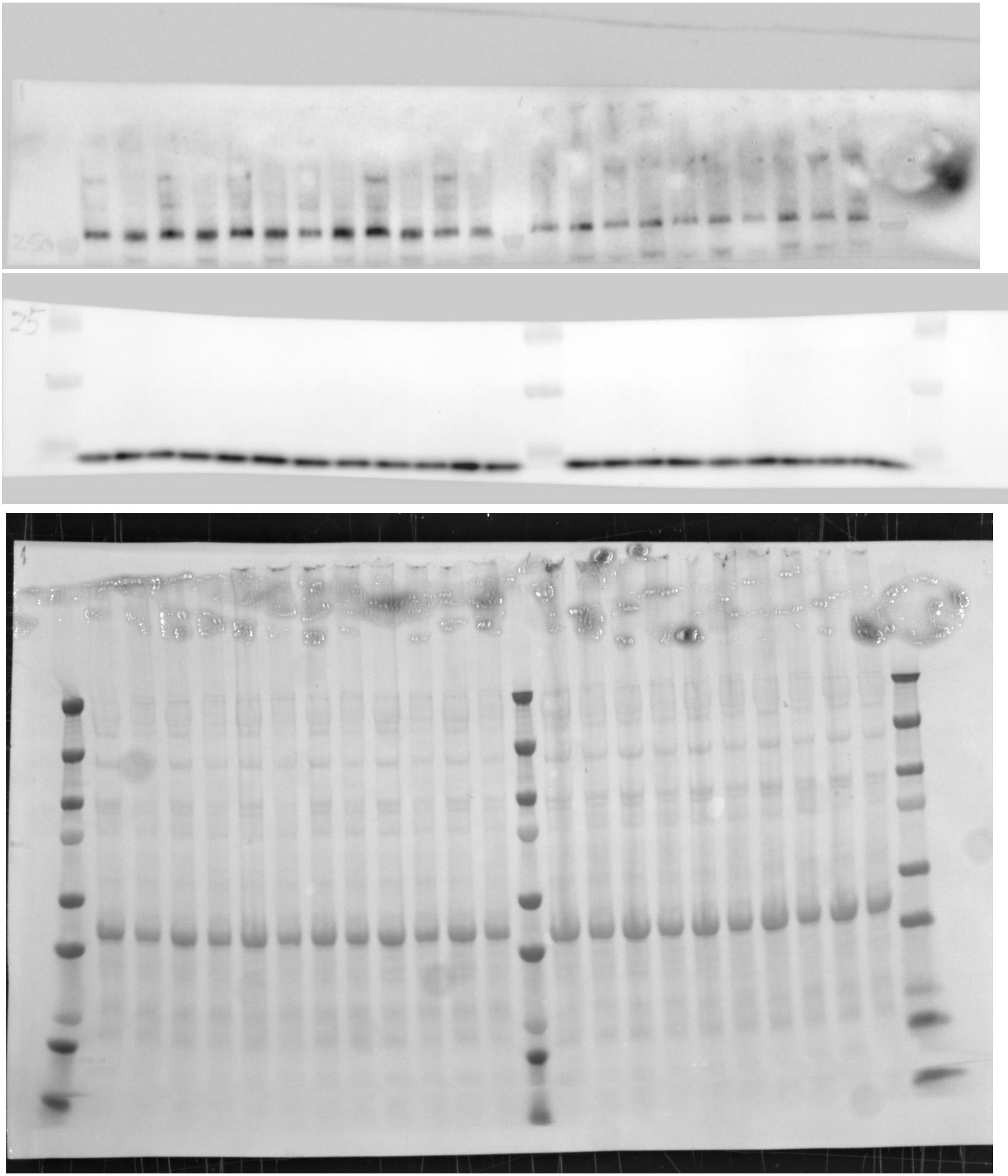

Fig. 5C

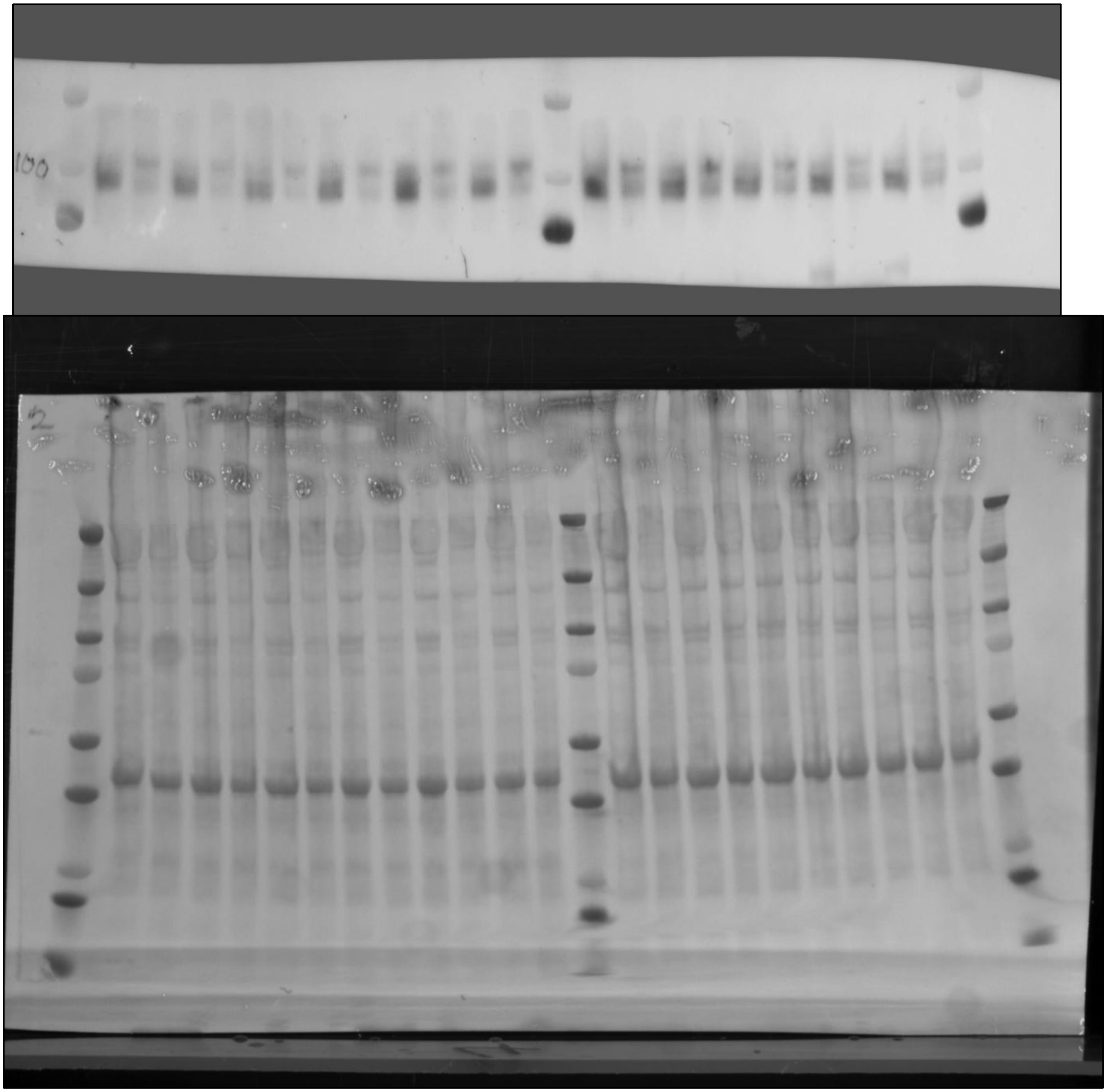

Fig. 6A

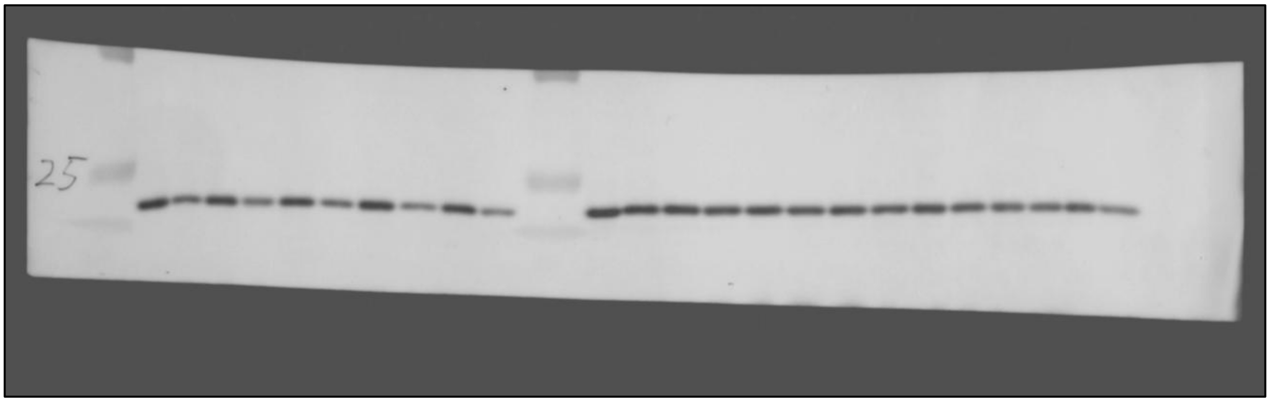

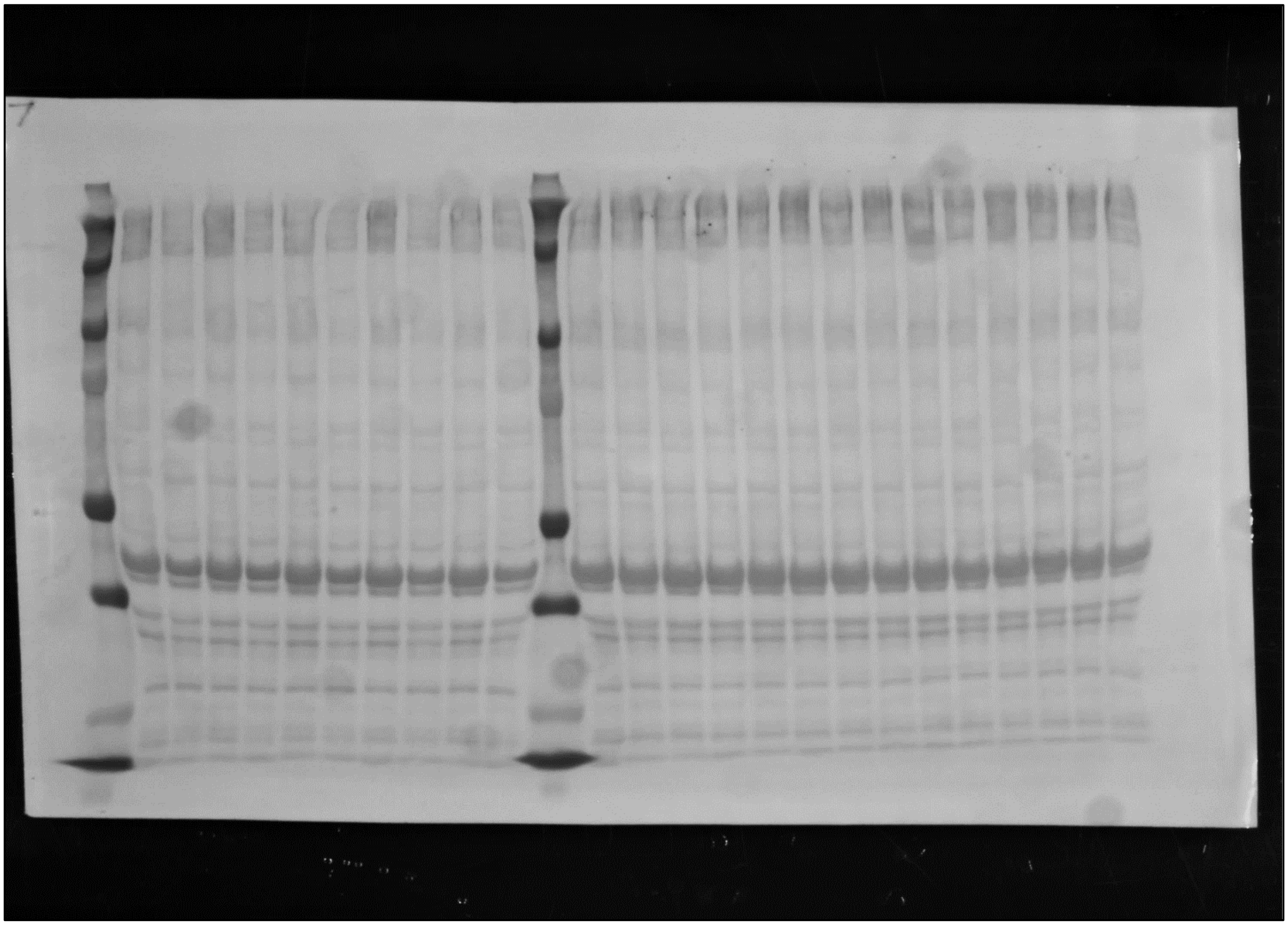

Fig. 6B

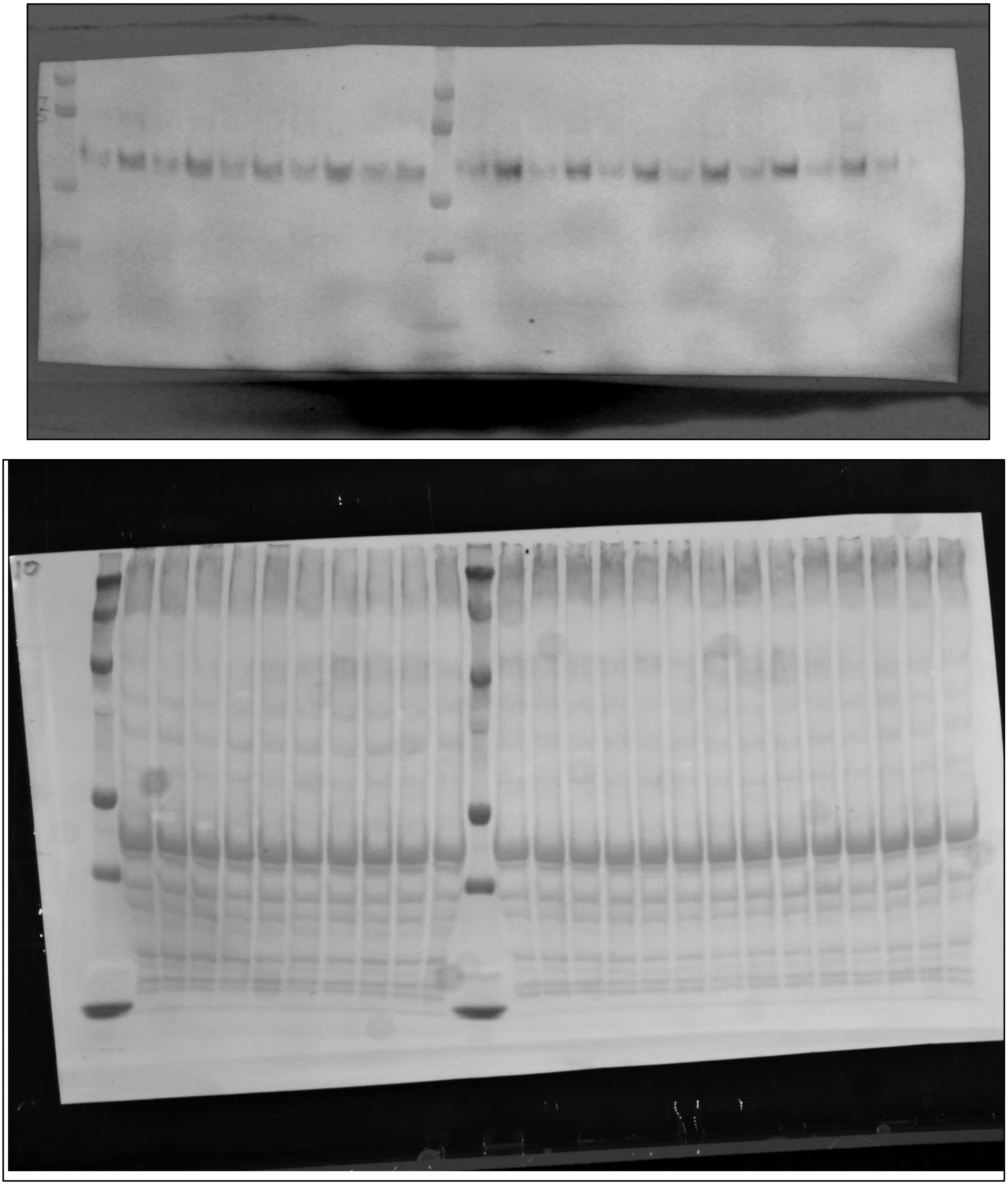

Fig. 6C

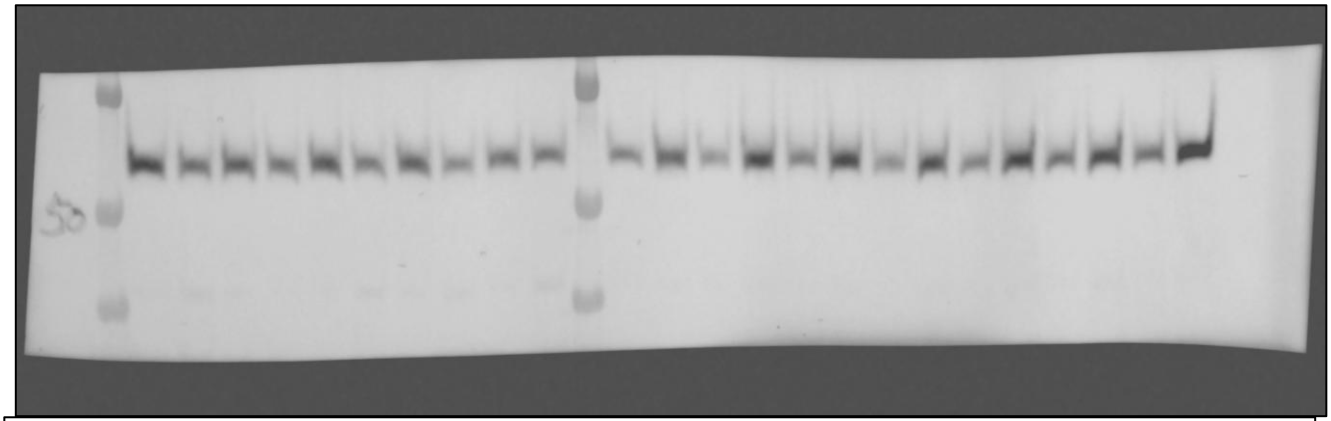

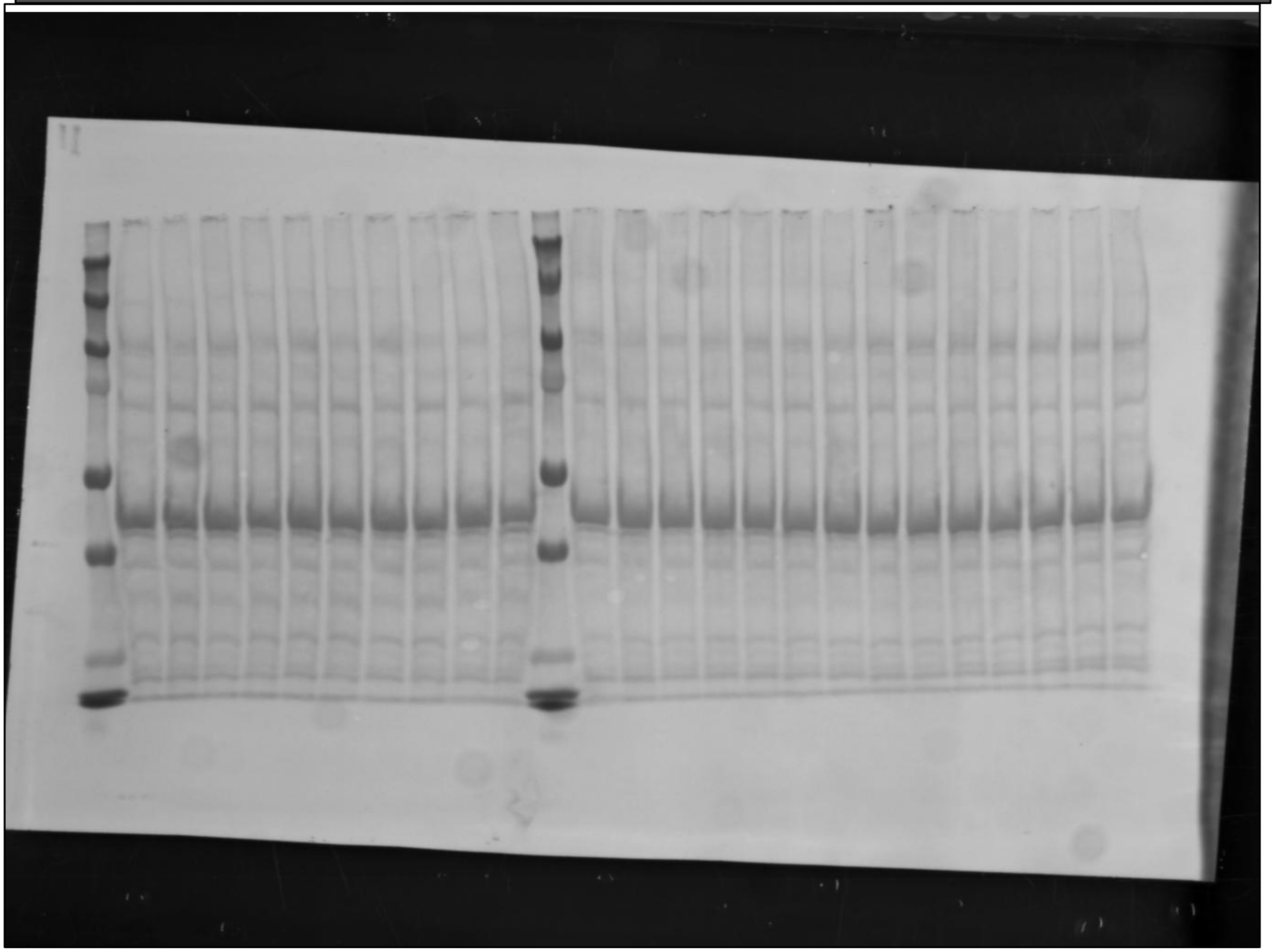

Fig. 6D

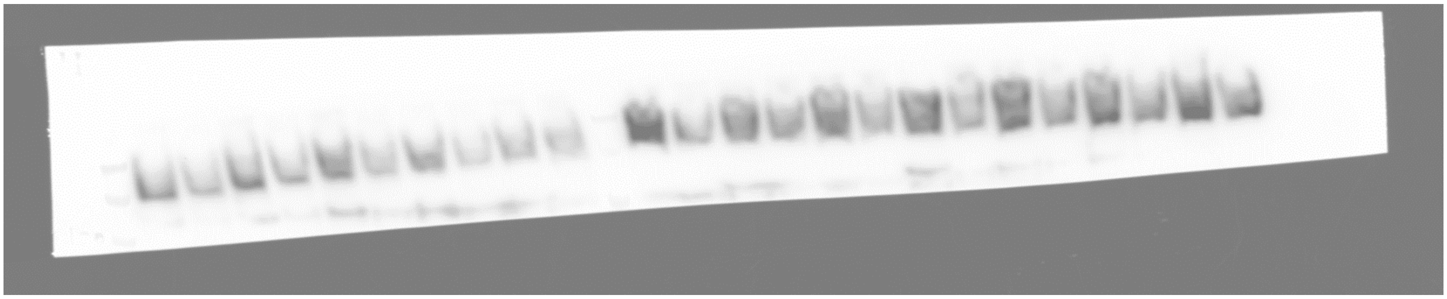

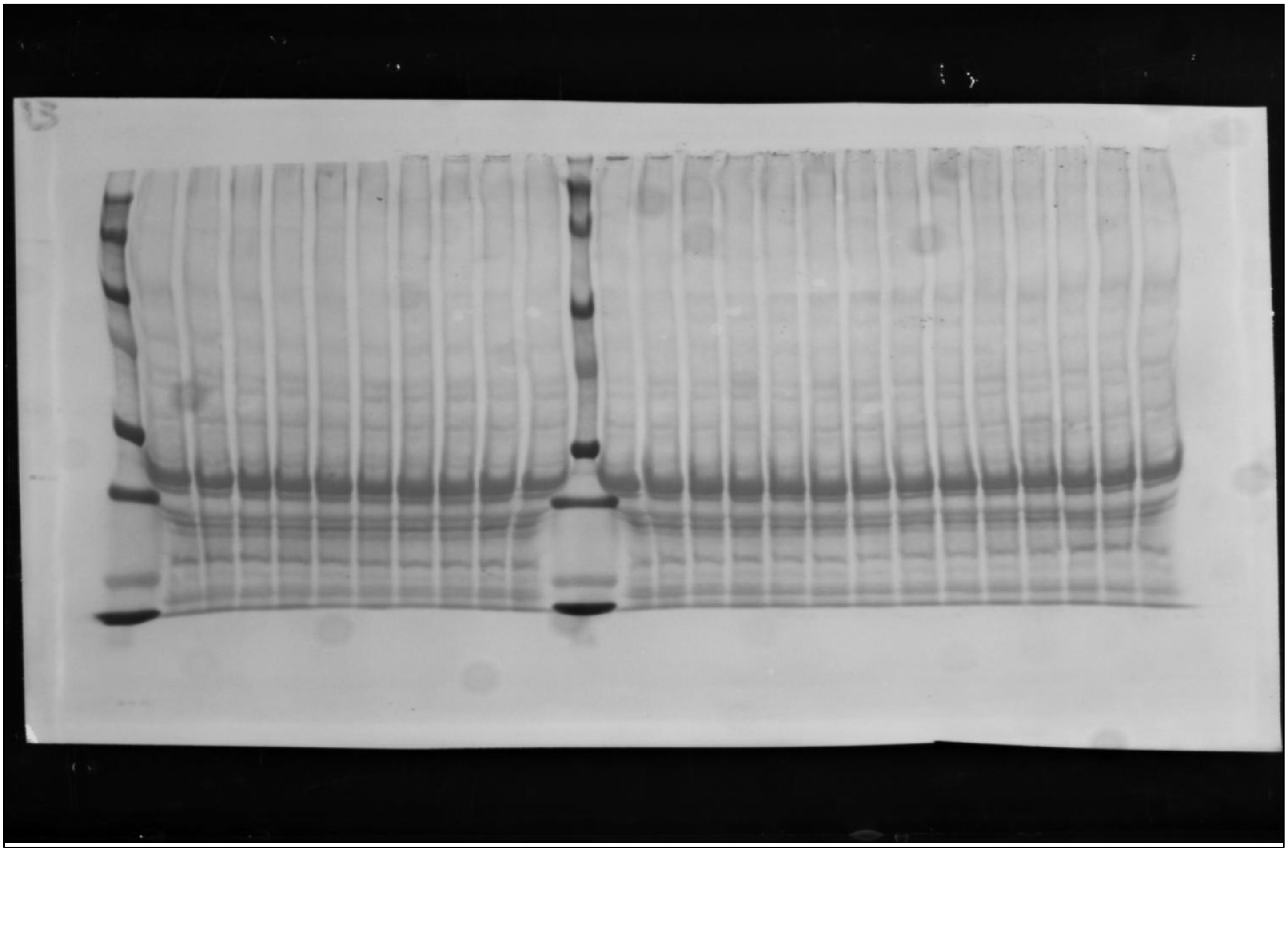

Fig. 7B

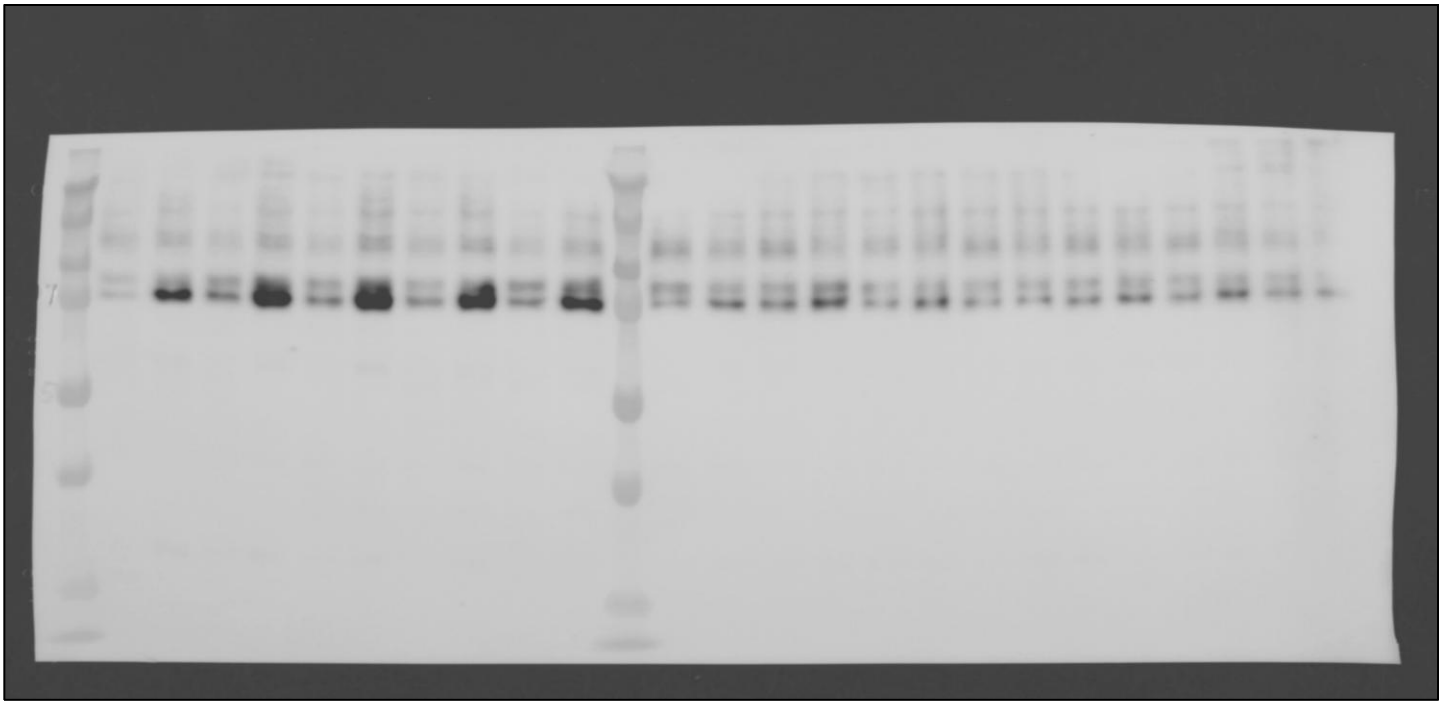

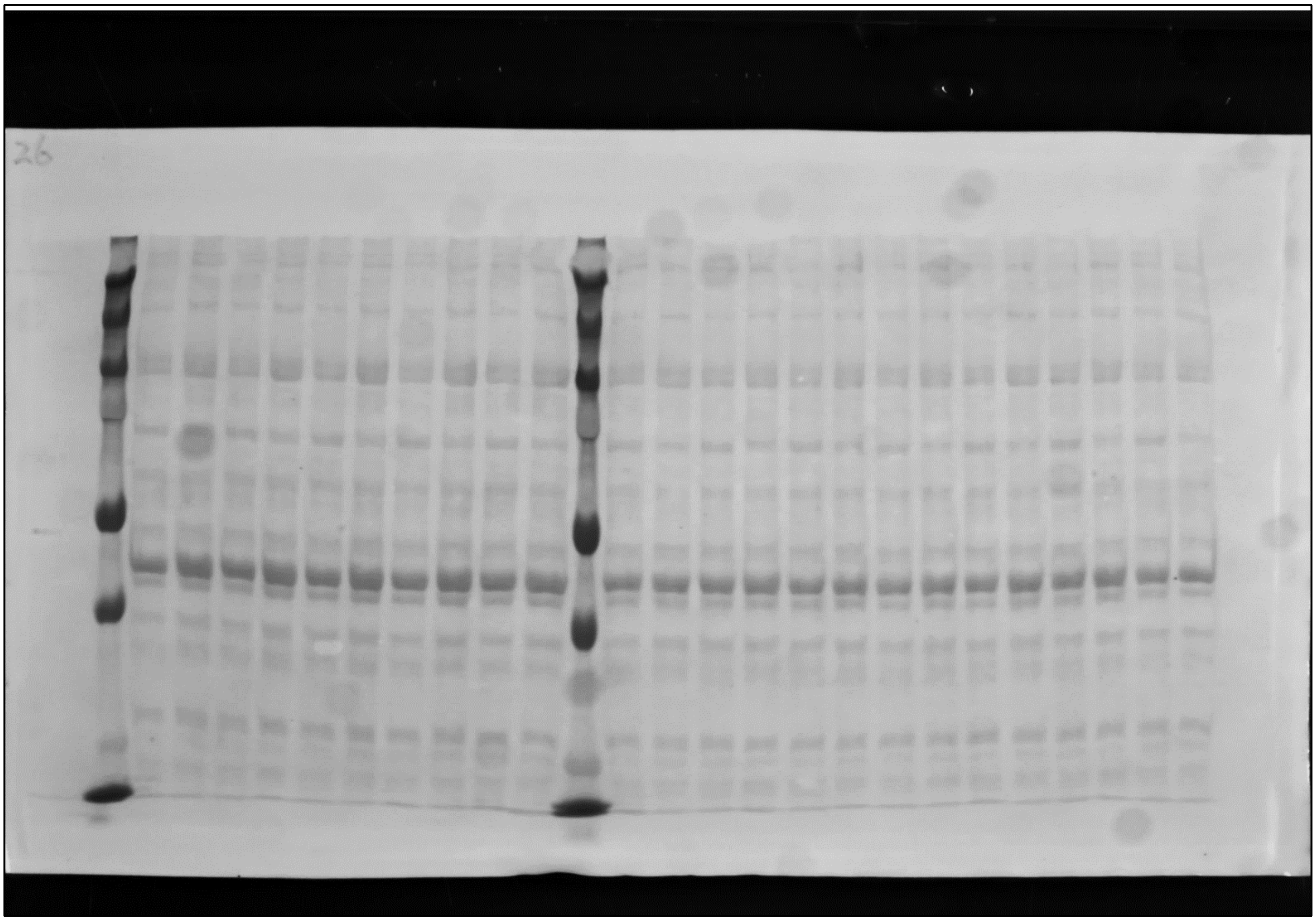

Fig. 7C

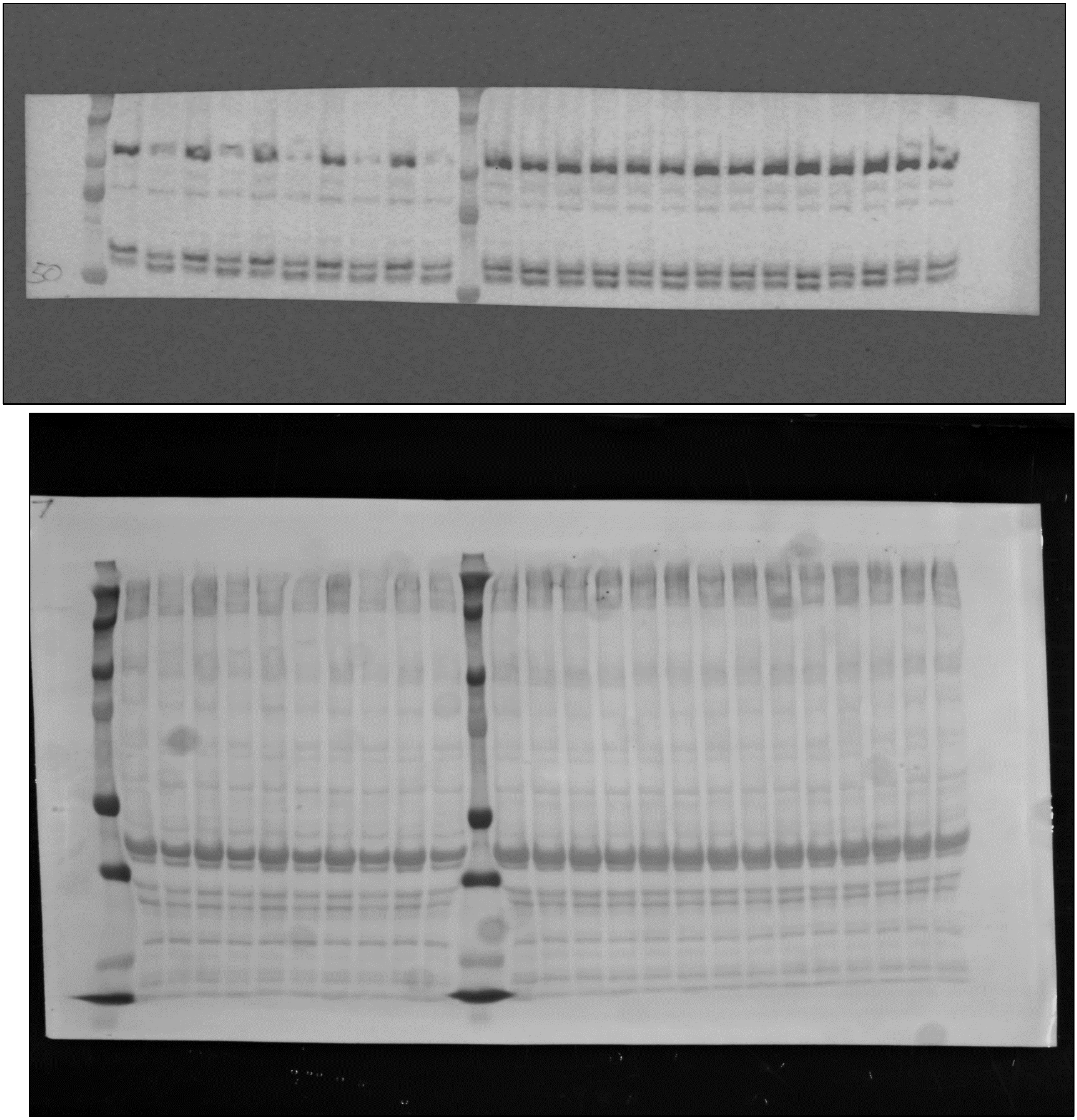

Fig. 7D

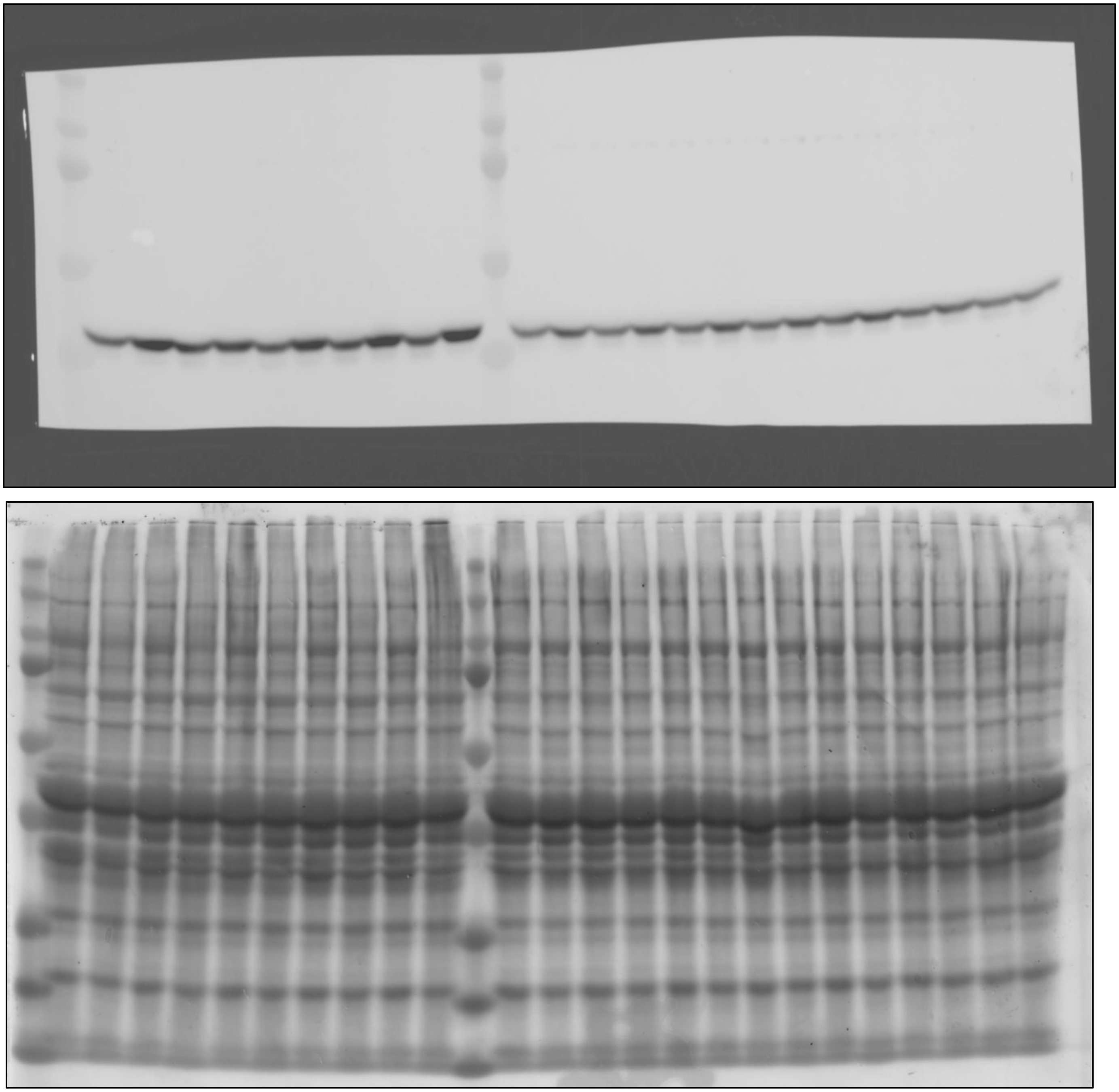

SFig. 1

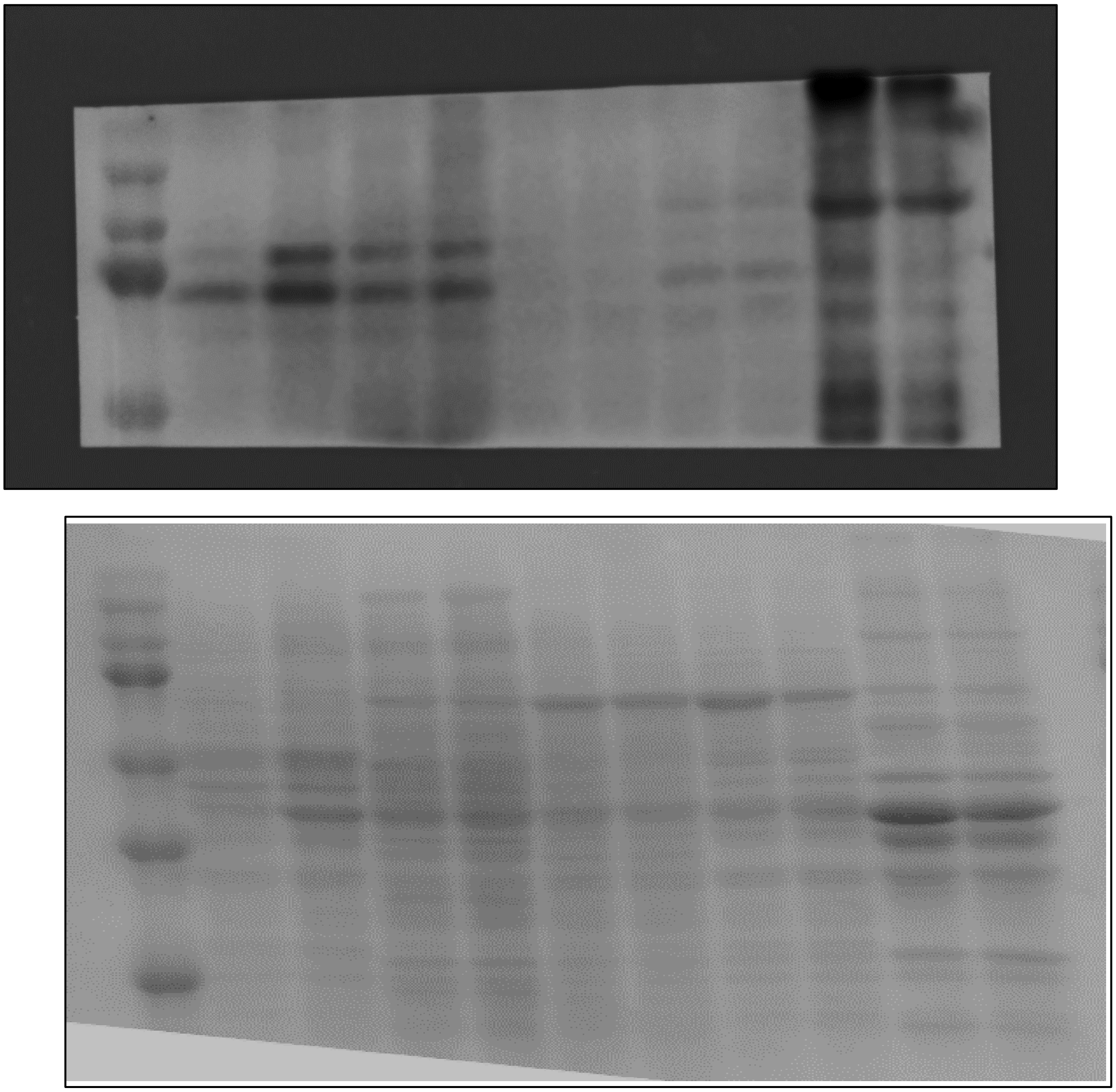

SFig. 2

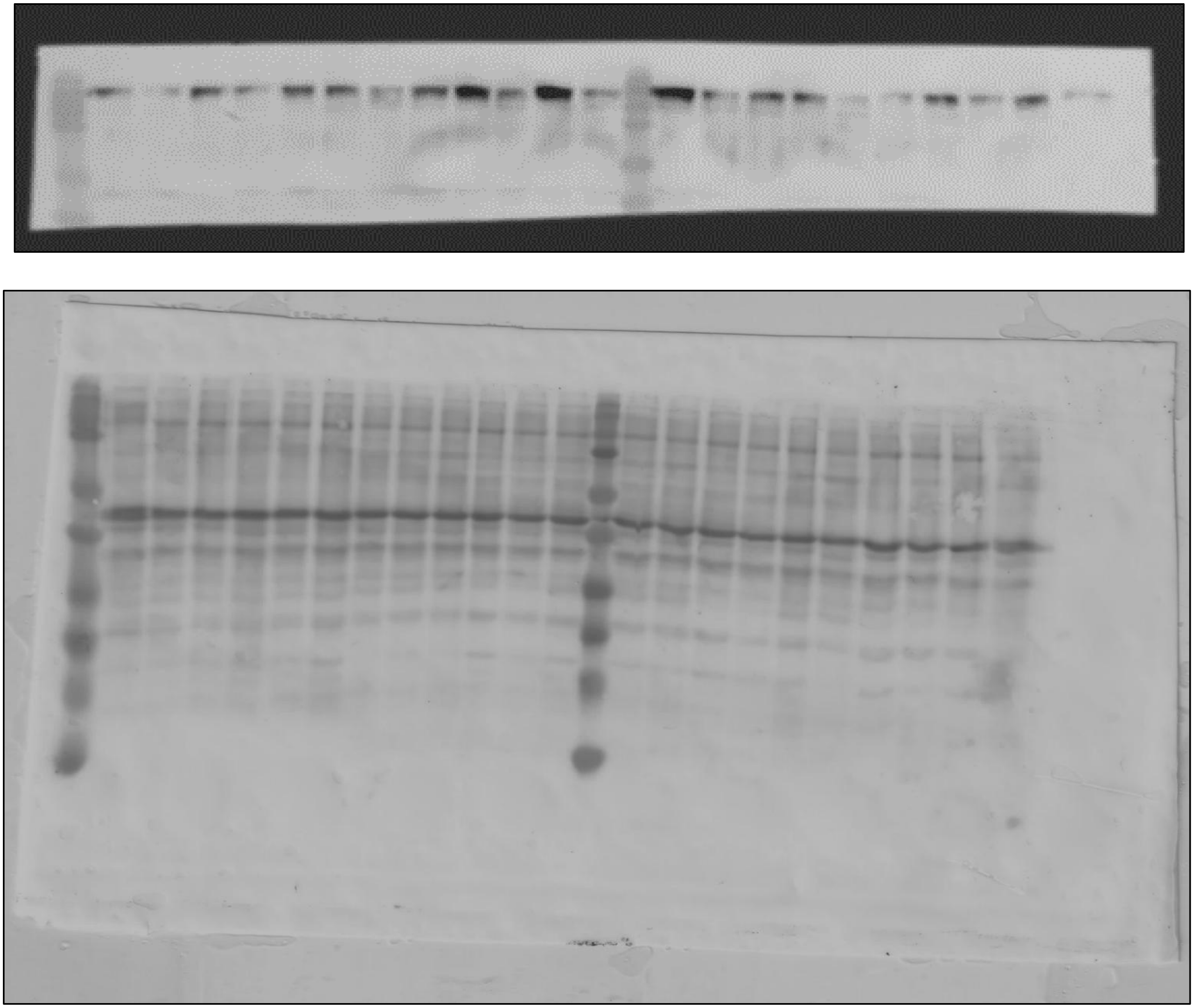

SFig. 3A

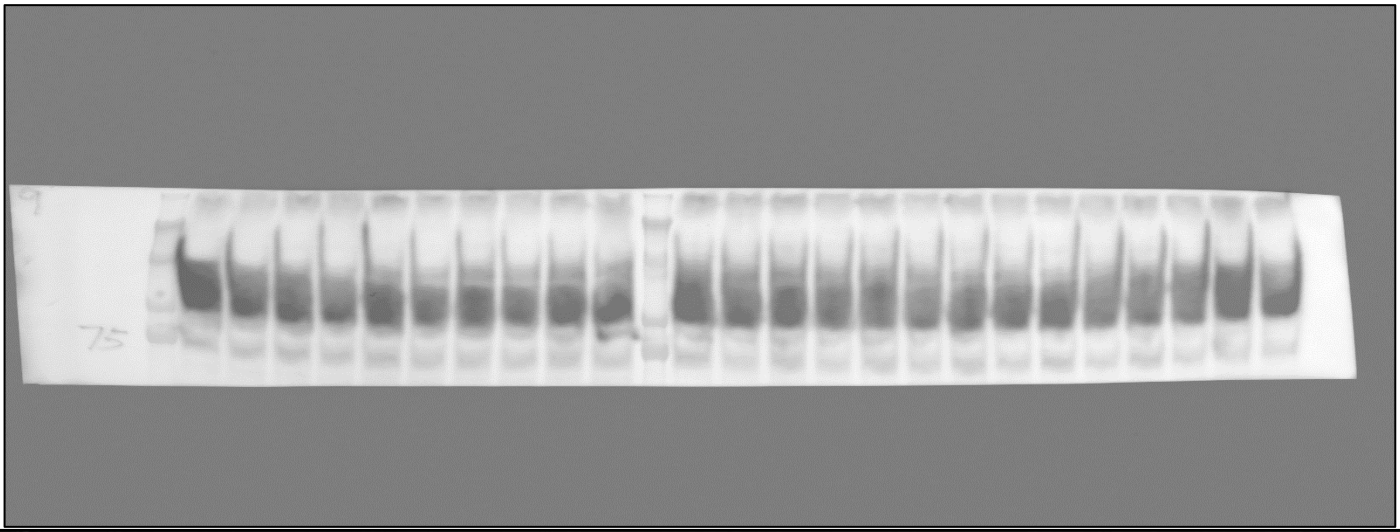

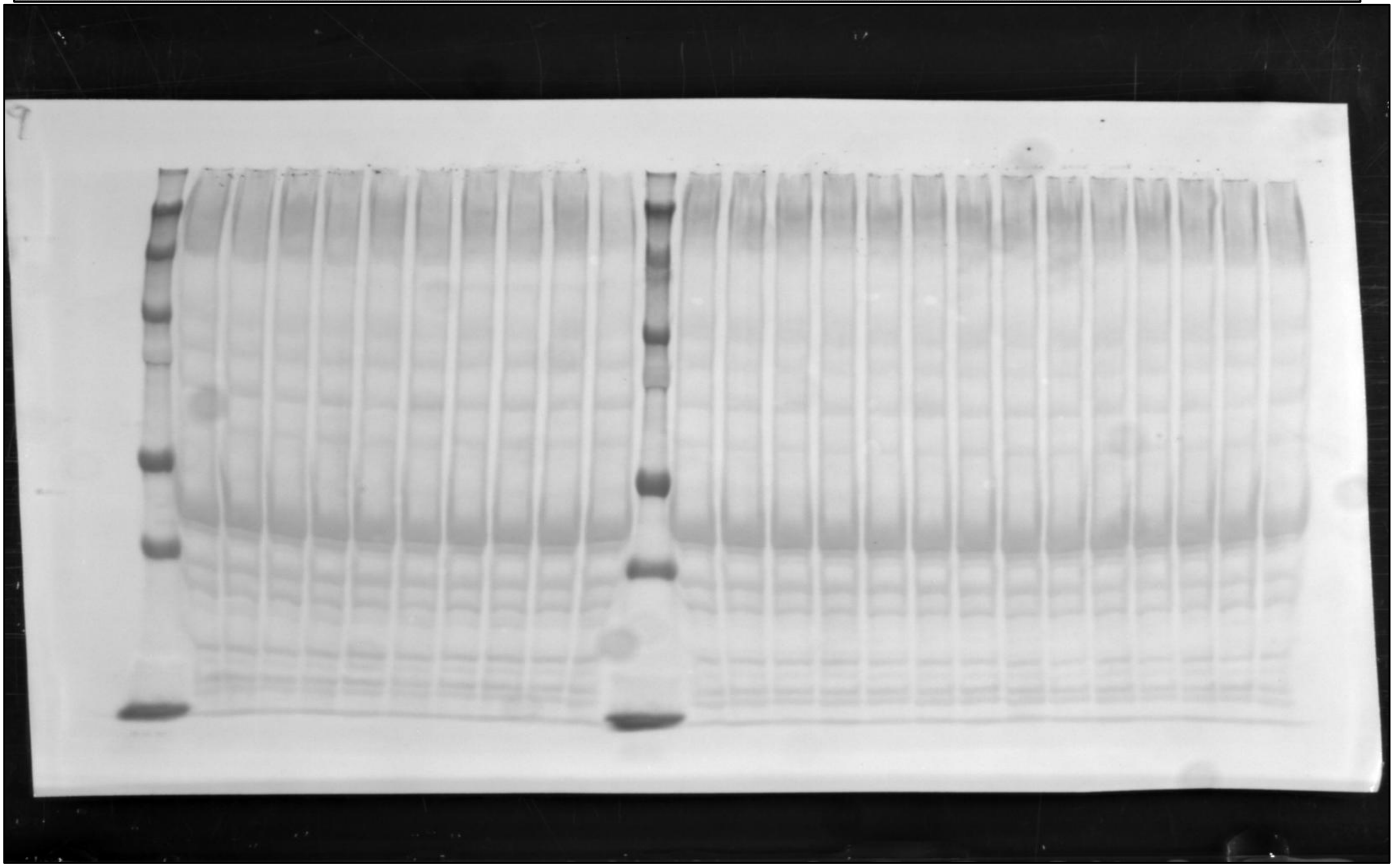

SFig. 3B

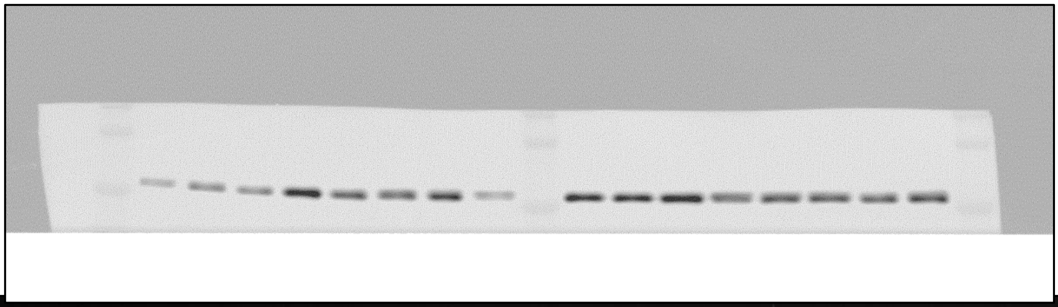

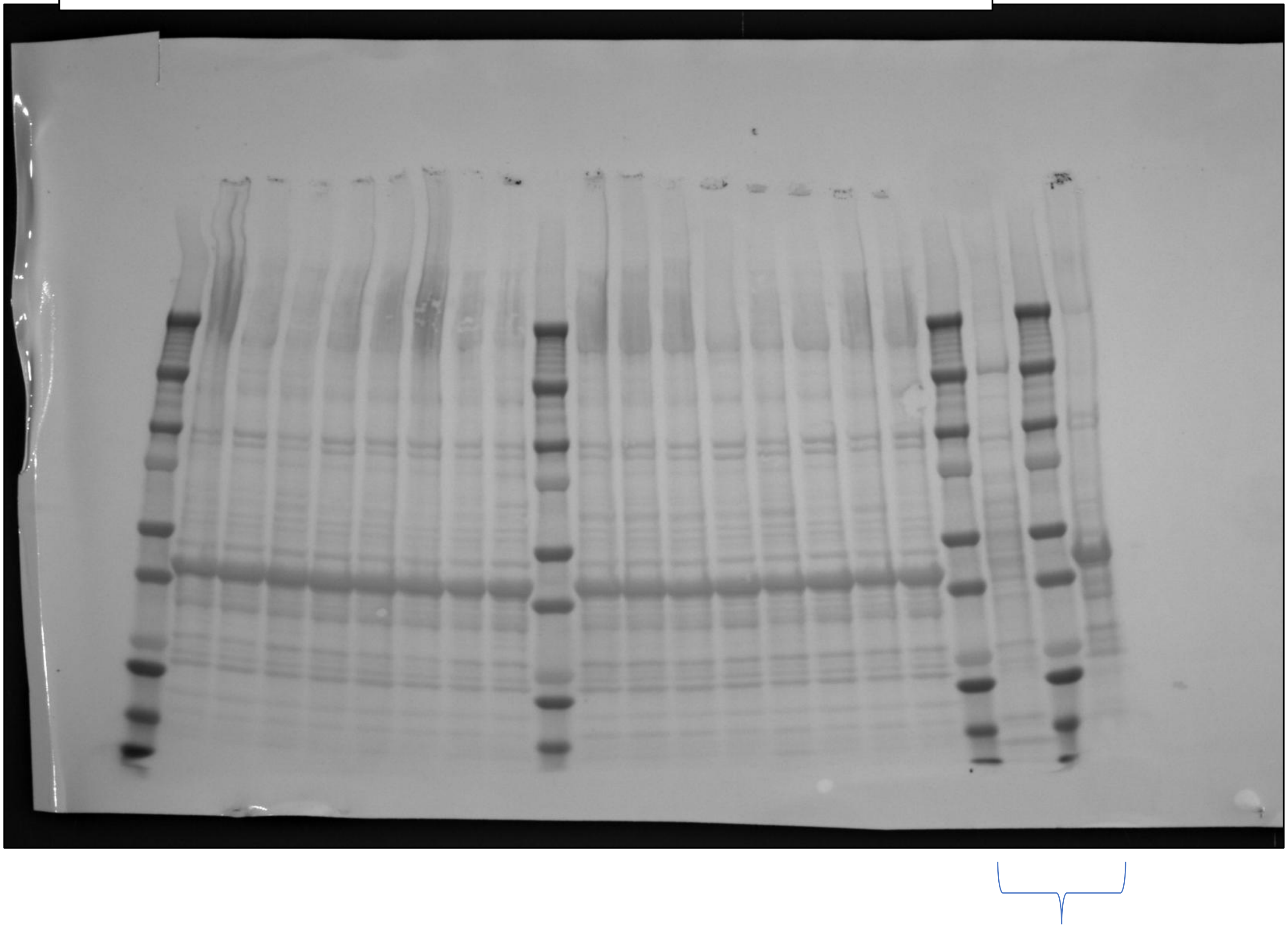

This part of blot was generated for a separate experiment and was not included in the present study.

SFig. 5D

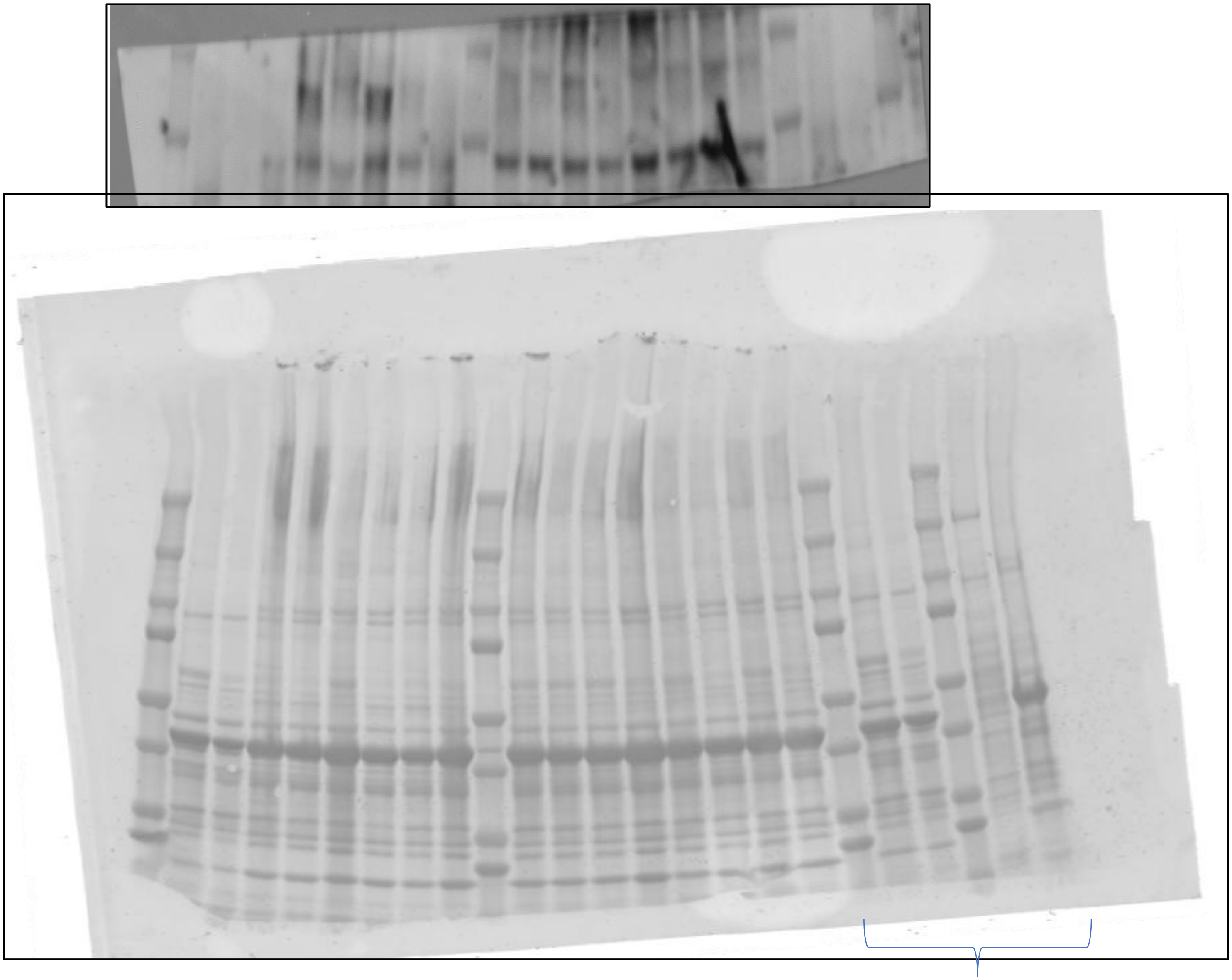

This part of blot was generated for a separate experiment and was not included in the present study.

SFig. 8C

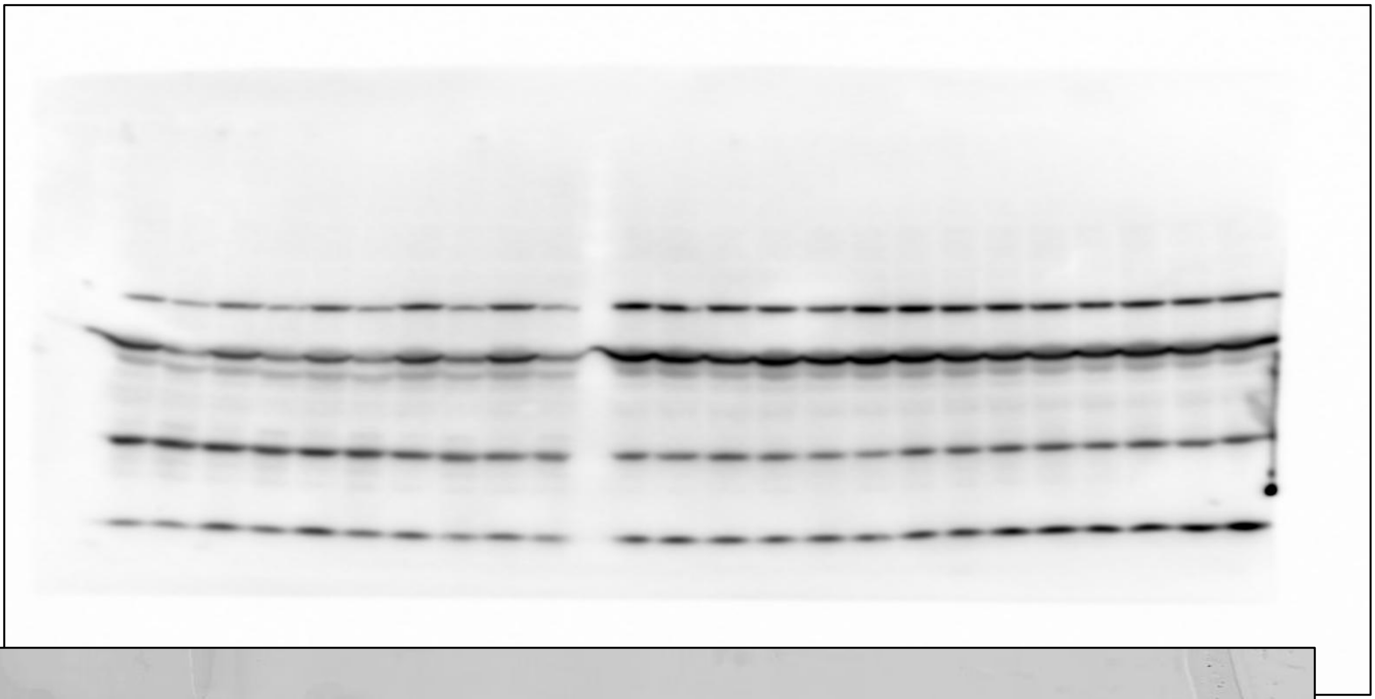

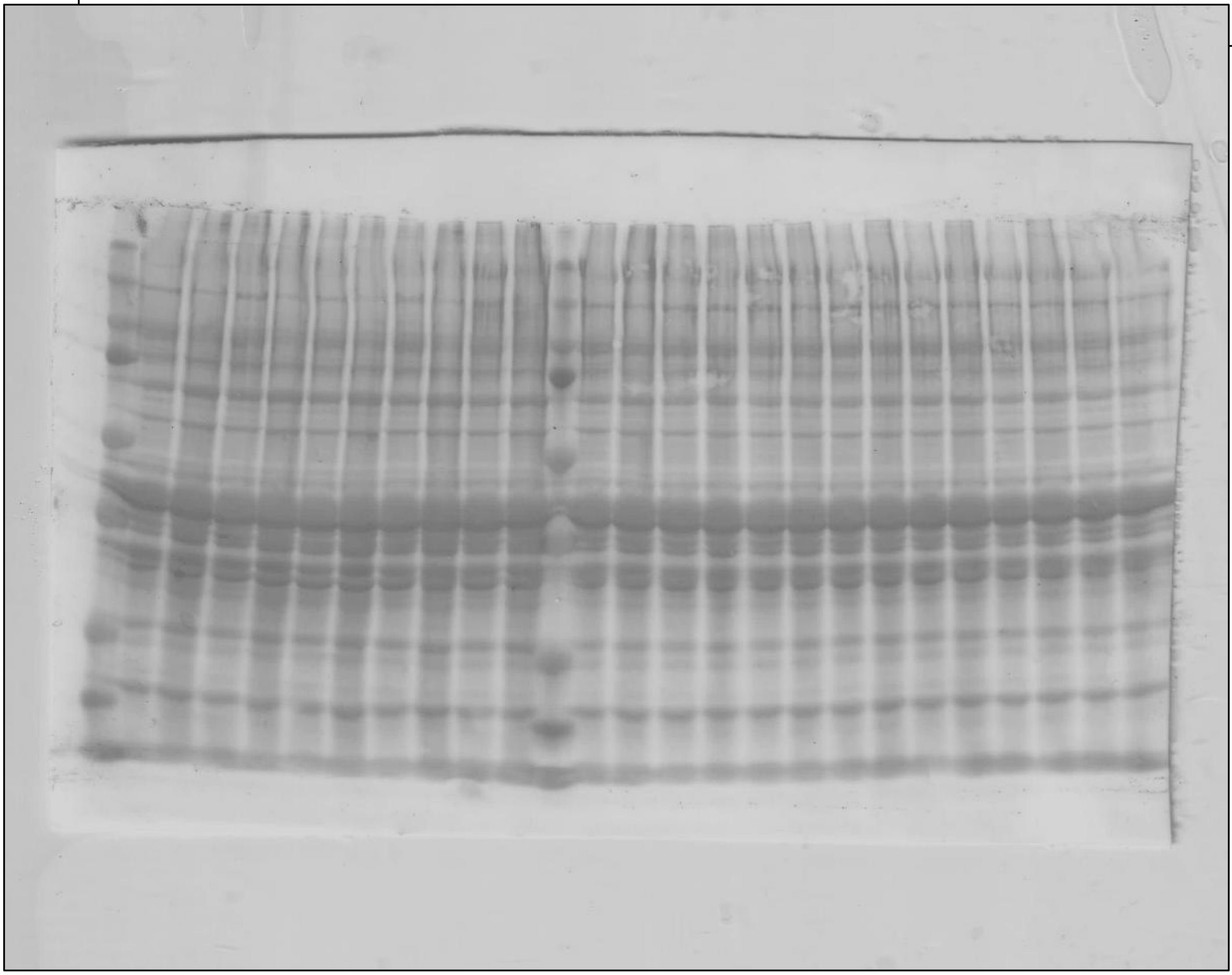

SFig. 11

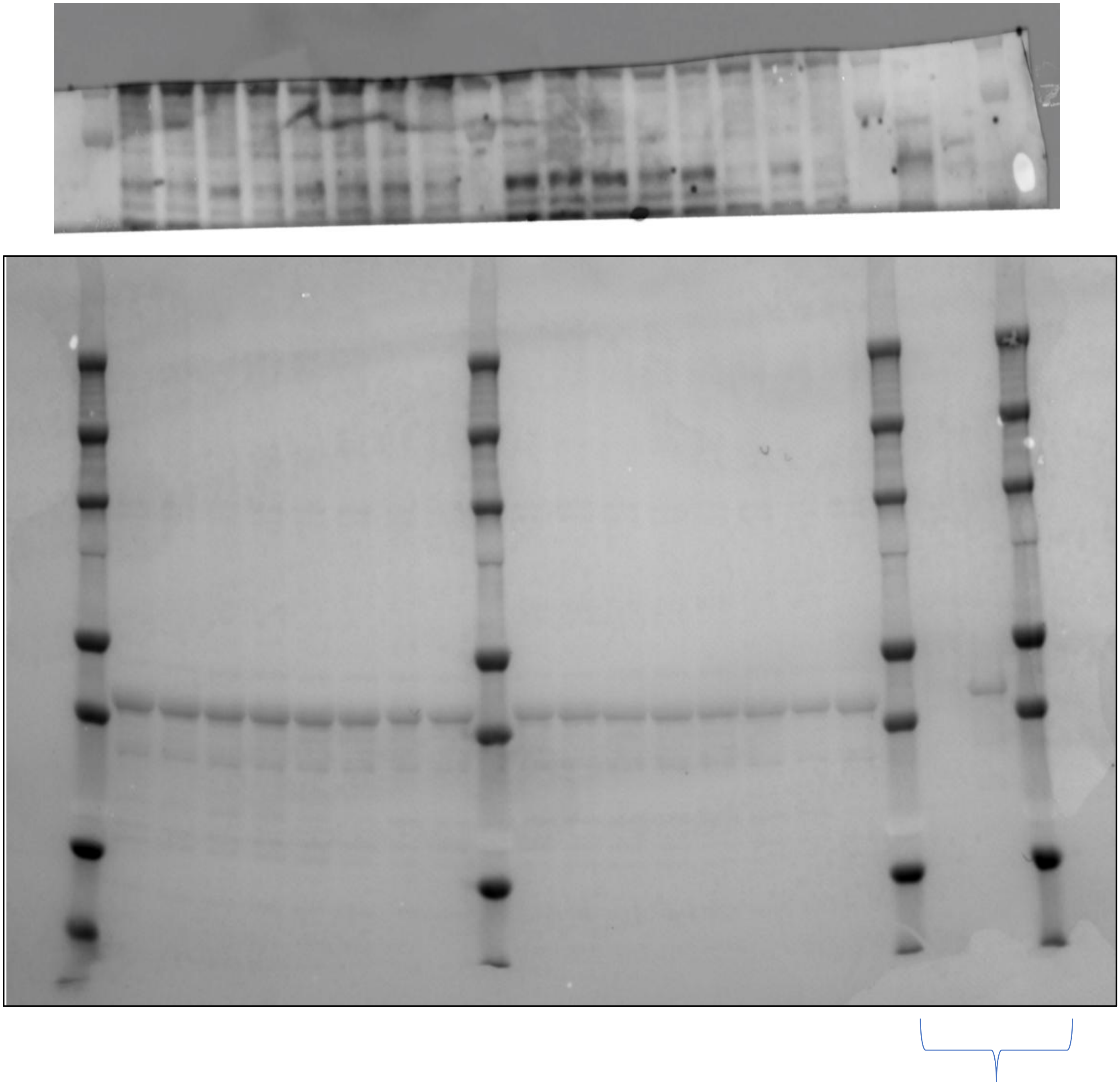

This part of blot was generated for a separate experiment and was not included in the present study.
